# Validating objective and scalable speech markers of depression across two independent psychiatric cohorts

**DOI:** 10.1186/s12991-026-00691-0

**Published:** 2026-08-20

**Authors:** Felix Menne, Felix Dörr, Johannes Tröger, Alexandra König, Julia Schräder, Diana Immel, René Hurlemann, Simon Barton, Lisa Wagels

**Affiliations:** 1 ki:elements GmbH, Bleichstr. 27, Saarbrücken, 66111 Germany; 2 Cobtek (Cognition-Behaviour- Technology) Lab, University Côte d’azur, Nice, France; 3 Clinique Gériatrique du Cerveau et du Mouvement, Université Côte d’Azur, Centre Hospitalier et Universitaire, Centre Mémoire de Ressources et de Recherche, Nice, France; 4 Institute for Translational Neuroscience and Clinical Psychology, RWTH Aachen University, Aachen, Germany; 5 JARA-Translational Brain Medicine, Aachen, Germany; 6 Center for Computational Life Science, RWTH Aachen University, Aachen, Germany; 7 Department of Psychiatry & Psychotherapy, School of Medicine & Health Sciences, Carl von Ossietzky University of Oldenburg, Oldenburg, Germany; 8 Karl Jaspers Clinic, Bad Zwischenahn, Germany

**Keywords:** Major depressive disorder, Depression, Speech markers, Machine learning, Validation study

## Abstract

**Background:**

Using speech as objective markers for major depressive disorder (MDD) has shown promise, yet their generalizability across clinical settings remains largely unvalidated.

**Objective:**

This study aimed to validate previously identified speech markers of depressive symptoms in an independent clinical cohort, thereby assessing their reproducibility and robustness for cross-site application.

**Methods:**

Speech data from two independent psychiatric cohorts (RWTH Aachen and University of Oldenburg, Germany) were analyzed, comprising 135 participants (71 healthy controls, 64 MDD patients). Participants completed a positive and a negative storytelling task, over 80 temporal, lexical, and spectral speech features were extracted from the acoustic signal. Statistical analyses assessed group differences and correlations with Beck Depression Inventory (BDI-II) scores. Machine learning models trained on the Aachen data were tested on the Oldenburg cohort.

**Results:**

Several temporal and spectral speech features, including utterance duration, pause duration, and MFCCs, were consistently associated with MDD diagnosis and symptom severity across both cohorts. Machine learning models trained on Aachen data achieved a classification accuracy (ROC-AUC) of 0.63 on the Oldenburg sample, demonstrating above-chance but modest transfer performance. Voice quality features (shimmer, jitter) showed more variable associations: partial correlations indicated some significant effects (e.g., shimmer and jitter during positive storytelling), whereas moderation analyses revealed interaction effects, particularly for shimmer and jitter in negative storytelling, where MDD patients exhibited higher values in the Aachen cohort but lower values in the Oldenburg cohort compared to healthy controls.

**Conclusions:**

The study indicates that temporal and spectral markers of speech are relatively robust across independent clinical samples, whereas voice quality markers (shimmer, jitter) show site-dependent inconsistencies, acting as technical artifacts of varying recording conditions rather than robust biomarkers. While current speech-based classifiers remain less accurate than established self-report measures, their integration with clinical scores offers a more balanced trade-off between sensitivity and specificity. Future work should prioritize systematic evaluation across elicitation tasks, languages, and longitudinal settings to delineate which speech features are transferable and which are task-specific.

**Supplementary Information:**

The online version contains supplementary material available at 10.1186/s12991-026-00691-0.

## Introduction

Psychiatric diagnosis and monitoring, particularly in the context of Major Depressive Disorder (MDD), continue to face critical limitations stemming from the subjectivity of traditional clinical assessments [1]. Standard methods, such as clinician interviews and patient self-reports, rely heavily on qualitative judgments and subjective interpretations [2] which may be biased [3, 4]. This may lead to potential compromising diagnostic accuracy and reducing inter-rater reliability. Furthermore, MDD is a multifaceted and varied condition [5], characterized by over a thousand distinct symptom combinations and hundreds of possibilities to fulfill the diagnostic criteria [6, 7]. These challenges persist despite well-established diagnostic criteria, such as those in the DSM-V, and underscore the pressing need for objective, quantifiable tools that can complement clinical judgment and improve the reliability of psychiatric evaluations [8, 9]. The integration of such tools is especially vital in the context of long-term care, where depressive symptoms may fluctuate between clinical visits [10, 11]. Without regular monitoring, symptom relapse can go undetected until it escalates into a full episode, often delaying intervention. In this light, automated and scalable tools for continuous and remote assessment could offer clinicians a more responsive approach to managing depression [12, 13].

In recent years, the integration of digital phenotyping and computational methods into mental health research has opened new pathways for enhancing psychiatric diagnostics and care [14]. Among these, automated speech analysis has emerged as a particularly promising method for capturing behavioral and physiological markers associated with psychiatric disorders such as MDD and schizophrenia [15–18]. Speech, as a naturally produced, non-invasive, and ubiquitous form of human behavior, reflects a range of cognitive and emotional states. It is modulated by underlying neurobiological processes, many of which are disrupted in mood disorders [19]. Consequently, speech analysis can serve as a passive, low-burden tool for identifying early signs of symptom recurrence [20]. This makes it highly suitable for remote monitoring applications [21], where frequent in-person evaluations are impractical. For example, digital health tools leveraging speech could alert clinicians to subtle but clinically significant changes in a patient’s condition, enabling timely interventions and relapse prevention. Such capabilities are particularly relevant in stepped-care models, telepsychiatry settings, and other frameworks aiming to extend care beyond the clinic [22]. Furthermore, widely used measures like the Beck Depression Inventory (BDI-II), the Montgomery‑Åsberg Depression Rating Scale (MADRS) and Hamilton Depression Rating Scale (HDRS) are inherently subjective. This reliance on self-reported and clinician ratings can introduce bias and variability into clinical trial outcomes, complicating objective assessments of drug efficacy [23]. In contrast, speech-based analysis offers a more granular, objective, and scalable alternative [24]. However, while these computational approaches provide quantifiable markers, it is important to acknowledge that Artificial Intelligence (AI) and Machine Learning (ML) models are not inherently free from bias. Such systems can incorporate diverse biases [25], for example if the training data are not representative of the target population’s diversity in terms of demographics, recording environments, or clinical manifestations. Nevertheless, when developed responsibly, these tools may augment or even enhance traditional assessments by providing real-time data that is less prone to the inter-rater variability of human observation.

Previous studies have demonstrated that speech characteristics in individuals with depression often deviate from typical patterns observed in healthy individuals. Commonly reported features include reduced pitch variability, slower speech rate, increased pause frequency, and decreased loudness, all of which contribute to the well-documented perception of depressive speech as flat, monotonic, and slowed [15, 19, 26, 27]. These acoustic markers are thought to be linked to the psychomotor retardation and affective flattening that frequently accompany depressive episodes. Linguistic markers of MDD further reflect these changes through altered communication patterns, though findings vary significantly based on task and context [28]. While patients often demonstrate a higher frequency of words related to sadness [29] and a “dichotomous” cognitive style characterized by the use of absolutist words [30], recent evidence suggests these patterns are accompanied by structural shifts, such as shorter sentence lengths and an increased reliance on impersonal pronouns and past-tense verbs [31].

In our prior research [15], conducted with a cohort of 44 individuals diagnosed with MDD and 52 healthy controls (HC) at Rheinisch-Westfälische Technische Hochschule (RWTH) University Hospital Aachen, Germany, we identified a set of speech-derived features such as jitter, shimmer, and loudness, that showed low to moderate positive correlations with the severity of depressive symptoms. Jitter and shimmer reflect micro-perturbations in vocal-fold vibration and are perceived as changes in voice quality, whereas pitch or loudness variability generally reflect slower modulations perceived as speech prosody and intonation. These acoustic perturbations are thought to reflect a lack of physiological arousal and reduced laryngeal muscle coordination, mechanisms often linked to psychomotor retardation, which result in more irregular vocal fold vibration and diminished vocal effort [32]. Furthermore, we found that patients with MDD exhibited significantly reduced pitch and loudness variability, increased temporal slowing, and lower lexical richness (all *p* < 0.02), while a 10-feature SVM model achieved high diagnostic accuracy (AUC = 0.93) to differentiate between MDD and HC, comparable to BDI-II-based classification. These findings supported the feasibility of using automated speech analysis as an objective proxy for clinical symptomatology.

However, a central challenge in digital mental health research is validating findings across independent populations and clinical settings [33]. While many initial studies demonstrate promising results within a single cohort, their generalizability often remains uncertain. These effects arise from technical variability, such as ambient noise and microphone sensitivity, and sample variability, including differences in age, sex, and disorder severity between cohorts. This issue is further complicated when different speech tasks are used across studies, making it problematic to draw broadly generalizable conclusions [34]. Consequently, ML approaches frequently yield low classification accuracy when tested on external cohorts that differ in task or context [35, 36]. Without cross-cohort validation, there is a risk that identified markers are site-specific or confounded by uncontrolled variables such as recording conditions, local diagnostic practices (e.g., using varying clinical assessments and questionnaires), or demographic differences. Clinical translation requires speech-based features that are robust, replicable, interpretable, and consistent across diverse settings. Variability in study designs, tasks, and populations has often hindered comparability of speech markers [37]. Evidence that certain features remain significant across different clinical sites is a critical step toward generalizable speech-based markers of MDD.

The present study addresses this gap by validating the speech markers identified in a sample of MDD patients and HC assessed at one clinical site (Aachen, Germany) in an independent population of MDD and HC participants recruited and examined at another clinic (Oldenburg, Germany).

To evaluate the robustness and generalizability of speech-based biomarkers across independent clinical settings, we tested the following exploratory and predictive hypotheses:

H1: We hypothesize that individuals with MDD will exhibit significantly altered speech patterns compared to healthy controls across an exploratory set of acoustic, spectral, and lexical markers (Table 1). While we expect results to align with previously observed signatures of psychomotor retardation, such as increased temporal slowing and reduced vocal variability, this study utilizes a broad, data-driven approach to identify the most robust markers across the extended feature space.

**Table 1:** Categories of analyzed categories with associated speech features

Feature group	Feature	Variable Name	Definition	Explanation
**Energy**				
	Shimmer	apq3_shimmer	Three-point period amplitude perturbation quotient (short-term amplitude variability in vocal fold vibrations)	Reflects the ‘stability’ or ‘noise’ in voice volume. High shimmer makes a voice sound crackly, harsh, or hoarse
	Shimmer	apq5_shimmer	Five-point period amplitude perturbation quotient	Reflects the ‘stability’ or ‘noise’ in voice volume. High shimmer makes a voice sound crackly, harsh, or hoarse
	Shimmer	apq11_shimmer	Eleven-point period amplitude perturbation quotient	Reflects the ‘stability’ or ‘noise’ in voice volume. High shimmer makes a voice sound crackly, harsh, or hoarse
	Shimmer	dda_shimmer	Dynamic Decline Amplitude Shimmer (average absolute differences between the amplitudes of consecutive periods)	Reflects the ‘stability’ or ‘noise’ in voice volume. High shimmer makes a voice sound crackly, harsh, or hoarse
	Harmonics-to-Noise Ratio	hnr_mean	Mean Harmonic-to-Noise Ratio (HNR) in decibels, calculated using the cepstral analysis of the acoustic signal	The ratio of ‘pure’ voice to ‘breathiness’ or noise. Lower values indicate a more breathy or raspy voice quality
	Harmonics-to-Noise Ratio	hnr_sd	Standard deviation of hnr_mean	The ratio of ‘pure’ voice to ‘breathiness’ or noise. Lower values indicate a more breathy or raspy voice quality
	Shimmer	local_shimmer	Cycle-to-cycle variability in amplitude (as percentage of the average amplitude)	Reflects the ‘stability’ or ‘noise’ in voice volume. High shimmer makes a voice sound crackly, harsh, or hoarse
	Average Loudness	loudness_mean	Mean speech loudness	The overall volume level of the speech
	Loudness Variability	loudness_sd	Standard deviation of loudness_mean	The range of volume changes. Low variability leads to a ‘flat’ or ‘monotonous’ volume, typical of a subdued voice
	Loudness Peak Rate	rate_loudness_peaks	Frequency of loudness peaks in a given time period	How often the speaker emphasizes words with volume bursts. A lower rate suggests a less energetic or ‘flatter’ delivery
	Shimmer	shimmer_local_dB_mean	Cycle-to-cycle variability in amplitude in decibels	Reflects the ‘stability’ or ‘noise’ in voice volume. High shimmer makes a voice sound crackly, harsh, or hoarse
	Shimmer	shimmer_local_dB_sd	Standard deviation of difference of shimmer_local_dB_mean	Reflects the ‘stability’ or ‘noise’ in voice volume. High shimmer makes a voice sound crackly, harsh, or hoarse
**Frequency**				
	Jitter	ddp_jitter	Dynamic Decline Perturbation in jitter (average absolute differences between the fundamental frequencies of consecutive periods)	Reflects micro-instability in vocal pitch. High jitter makes a voice sound shaky or ‘unsteady’
	Pitch Range	f0_range	Range of the fundamental frequency (F0) of vocal fold vibrations	The distance between the highest and lowest notes spoken. A narrow range sounds ‘robotic’ or monotonic
	Formant Bandwidth (F1)	f1_bandwidth_mean	Mean bandwidth of F1 formant	Related to vocal tract resonance. Narrower variations often correlate with ‘mumbled’ speech where articulators move less
	Formant Bandwidth (F1)	f1_bandwidth_sd	Standard deviation of f1_bandwidth_mean	Related to vocal tract resonance. Narrower variations often correlate with ‘mumbled’ speech where articulators move less
	Formant Frequency (F1)	f1_frequency_mean	Mean frequency of F1 formant	Indicates the ‘shape’ of the vocal tract. Changes here reflect how vowels are articulated (e.g., clear vs. mumbled)
	Formant Frequency (F1)	f1_frequency_sd	Standard deviation of f1_frequency_mean	Indicates the ‘shape’ of the vocal tract. Changes here reflect how vowels are articulated (e.g., clear vs. mumbled)
	Formant Bandwidth (F2)	f2_bandwidth_mean	Mean bandwidth of F2 formant	Related to vocal tract resonance. Narrower variations often correlate with ‘mumbled’ speech where articulators move less
	Formant Bandwidth (F2)	f2_bandwidth_sd	Standard deviation of f2_bandwidth_mean	Related to vocal tract resonance. Narrower variations often correlate with ‘mumbled’ speech where articulators move less
	Formant Frequency (F2)	f2_frequency_mean	Mean frequency of F2 formant	Indicates the ‘shape’ of the vocal tract. Changes here reflect how vowels are articulated (e.g., clear vs. mumbled)
	Formant Frequency (F2)	f2_frequency_sd	Standard deviation of f2_frequency_mean	Indicates the ‘shape’ of the vocal tract. Changes here reflect how vowels are articulated (e.g., clear vs. mumbled)
	Formant Bandwidth (F3)	f3_bandwidth_mean	Mean bandwidth of F3 formant	Related to vocal tract resonance. Narrower variations often correlate with ‘mumbled’ speech where articulators move less
	Formant Bandwidth (F3)	f3_bandwidth_sd	Standard deviation of f3_bandwidth_mean	Related to vocal tract resonance. Narrower variations often correlate with ‘mumbled’ speech where articulators move less
	Formant Frequency (F3)	f3_frequency_mean	Mean frequency of F3 formant	Indicates the ‘shape’ of the vocal tract. Changes here reflect how vowels are articulated (e.g., clear vs. mumbled)
	Formant Frequency (F3)	f3_frequency_sd	Standard deviation of f3_frequency_mean	Indicates the ‘shape’ of the vocal tract. Changes here reflect how vowels are articulated (e.g., clear vs. mumbled)
	Jitter	jitter_local_mean	Deviations in individual consecutive F0 period lengths (perceived as uneven or irregular voice)	Reflects micro-instability in vocal pitch. High jitter makes a voice sound shaky or ‘unsteady’
	Jitter	jitter_local_sd	Standard deviation of jitter_local_mean	Reflects micro-instability in vocal pitch. High jitter makes a voice sound shaky or ‘unsteady’
	Jitter	local_absolute_jitter	Cycle-to-cycle variability in the fundamental frequency of vocal fold vibrations	Reflects micro-instability in vocal pitch. High jitter makes a voice sound shaky or ‘unsteady’
	Pitch Variability	pitch_coefficient_of_variation	Coefficient of variation of pitch	How much the voice ‘sings’ or changes pitch. Low variability sounds monotonic
	Pitch Statistics	pitch_first_quartile	First quartile of pitch	Various measures (min, max, percentiles) capturing the distributional characteristics of the speaker’s vocal pitch
	Pitch Statistics	pitch_kurtosis	Degree of peakedness or flatness of the fundamental frequency (F0) distribution	Various measures (min, max, percentiles) capturing the distributional characteristics of the speaker’s vocal pitch
	Pitch Statistics	pitch_linear_regression_mse	Mean squared error of a linear regression model applied to fundamental frequency (F0) values	Various measures (min, max, percentiles) capturing the distributional characteristics of the speaker’s vocal pitch
	Pitch Statistics	pitch_linear_regression_offset	Intercept of a linear regression model applied to fundamental frequency (F0) values	Various measures (min, max, percentiles) capturing the distributional characteristics of the speaker’s vocal pitch
	Pitch Statistics	pitch_linear_regression_slope	Slope coefficient of a linear regression model applied to the fundamental frequency (F0) values	Various measures (min, max, percentiles) capturing the distributional characteristics of the speaker’s vocal pitch
	Pitch Statistics	pitch_max	Maximum pitch	Various measures (min, max, percentiles) capturing the distributional characteristics of the speaker’s vocal pitch
	Average Pitch (F0)	pitch_mean	Mean pitch	The average highness or lowness of the voice. Note: F0 is the physical frequency, while pitch is what we perceive
	Pitch Statistics	pitch_min	Minimum pitch	Various measures (min, max, percentiles) capturing the distributional characteristics of the speaker’s vocal pitch
	Pitch Statistics	pitch_percentile_1	First percentile of pitch	Various measures (min, max, percentiles) capturing the distributional characteristics of the speaker’s vocal pitch
	Pitch Statistics	pitch_percentile_20	20th percentile of pitch	Various measures (min, max, percentiles) capturing the distributional characteristics of the speaker’s vocal pitch
	Pitch Statistics	pitch_percentile_20_80_range	Range of 20th to 80th percentile of pitch	Various measures (min, max, percentiles) capturing the distributional characteristics of the speaker’s vocal pitch
	Pitch Statistics	pitch_percentile_80	80th percentile of pitch	Various measures (min, max, percentiles) capturing the distributional characteristics of the speaker’s vocal pitch
	Pitch Statistics	pitch_percentile_99	99th percentile of pitch	Various measures (min, max, percentiles) capturing the distributional characteristics of the speaker’s vocal pitch
	Pitch Statistics	pitch_percentile_1_99_range	Range of 1st to 99th percentile of pitch	Various measures (min, max, percentiles) capturing the distributional characteristics of the speaker’s vocal pitch
	Pitch Statistics	pitch_q2_q1_range	Range of 1st to 2nd quartile of pitch	Various measures (min, max, percentiles) capturing the distributional characteristics of the speaker’s vocal pitch
	Pitch Statistics	pitch_q3_q1_range	Range of 1st to 3rd quartile of pitch	Various measures (min, max, percentiles) capturing the distributional characteristics of the speaker’s vocal pitch
	Pitch Statistics	pitch_q3_q2_range	Range of 2nd to 3rd quartile of pitch	Various measures (min, max, percentiles) capturing the distributional characteristics of the speaker’s vocal pitch
	Pitch Range	pitch_range	Range of pitch	The distance between the highest and lowest notes spoken. A narrow range sounds ‘robotic’ or monotonic
	Pitch Statistics	pitch_second_quartile	Second quartile of pitch	Various measures (min, max, percentiles) capturing the distributional characteristics of the speaker’s vocal pitch
	Pitch Statistics	pitch_skewness	Asymmetry of the fundamental frequency (F0) distribution	Various measures (min, max, percentiles) capturing the distributional characteristics of the speaker’s vocal pitch
	Pitch Variability	pitch_std	Standard deviation of pitch	How much the voice ‘sings’ or changes pitch. Low variability sounds monotonic
	Pitch Statistics	pitch_third_quartile	Third quartile of pitch	Various measures (min, max, percentiles) capturing the distributional characteristics of the speaker’s vocal pitch
	Jitter	ppq5_jitter	Five-point period perturbation quotient, quantifying the cycle-to-cycle variability in the fundamental frequency (F0) of vocal fold vibrations	Reflects micro-instability in vocal pitch. High jitter makes a voice sound shaky or ‘unsteady’
	Jitter	rap_jitter	Relative Average Perturbation jitter, to quantify the cycle-to-cycle variation in the fundamental frequency (F0) of vocal fold vibrations	Reflects micro-instability in vocal pitch. High jitter makes a voice sound shaky or ‘unsteady’
	Vocal Tremor	vocal_tremor	Vocal tremor, measuring the intensity of low-frequency F0 modulation, defined as the peak magnitude of F0 modulation within the 1.5–15 Hz band	A rhythmic ‘shaking’ in the voice, often heard when a speaker is strained or physically fatigued
**Lexical Richness**				
	Lexical Diversity Indices	brunets_index	Brunet’s Index, measure of lexical diversity	Mathematical measures of how varied the vocabulary is. Higher values mean a more complex and varied use of words
	Lexical Diversity Indices	honore_stat	Honoré’s statistic, measure to assess the lexical richness or diversity	Mathematical measures of how varied the vocabulary is. Higher values mean a more complex and varied use of words
	Word Repetitions	number_consecutive_repetitions	Number of consecutive repetitions	Counting how often the same words are repeated consecutively, which can indicate rumination or processing delays
	Type-Token Ratio	type_token_ratio	Proportion of unique words (types) to total words (tokens) in a text or speech sample	The ratio of unique words to total words. A low ratio indicates repetitive speech or a limited vocabulary
	Word Count	word_count	Number of words used	The total number of words spoken in the response
	Word Commonality	word_frequency_mean	Mean occurrence rate of each word token, calculated as the mean frequency of all tokens across the utterance	How common or rare the chosen words are in general language. Depressed speech often uses more common, less specific words
	Word Commonality	word_frequency_range	Range of word_frequency_mean	How common or rare the chosen words are in general language. Depressed speech often uses more common, less specific words
	Word Commonality	word_frequency_sd	Standard deviation of word_frequency_mean	How common or rare the chosen words are in general language. Depressed speech often uses more common, less specific words
**Sentiment**				
	Sentiment Analysis	mean_sentiment	Average emotional valence of the sentences (indicating if the whole answer was in general more positive, neutral, or negative)	Measures the emotional ‘tone’ of the words used (positive, neutral, or negative)
		negative_sentence_ratio	Number of sentences that are labeled as negative in relation to all sentences	
		neutral_sentence_ratio	Number of sentences that are labeled as neutral in relation to all sentences	
		positive_sentence_ratio	Number of sentences that are labeled as positive in relation to all sentences	
**Spectral**				
	Alpha Ratio	alpha_ratio_mean	Mean of the ratio of the summed energy from 50–1000 Hz and 1–5 kHz	A measure of ‘brightness’ in the voice. A higher ratio sounds clearer and more energized
	Alpha Ratio	alpha_ratio_sd	Standard deviation of alpha_ratio_mean	A measure of ‘brightness’ in the voice. A higher ratio sounds clearer and more energized
	MFCCs	average_mfccs_1	Average of the Mel-Frequency-Cepstral-Coefficient 1 (Measurement to capture fundamental frequency characteristics of human speech)	Captures the ‘timbre’ or broad ‘texture’ of the voice. It’s the standard way computers distinguish different voice qualities
	MFCCs	average_mfccs_2	Average of the Mel-Frequency-Cepstral-Coefficient 2	Captures the ‘timbre’ or broad ‘texture’ of the voice. It’s the standard way computers distinguish different voice qualities
	MFCCs	average_mfccs_3	Average of the Mel-Frequency-Cepstral-Coefficient 3	Captures the ‘timbre’ or broad ‘texture’ of the voice. It’s the standard way computers distinguish different voice qualities
	MFCCs	average_mfccs_4	Average of the Mel-Frequency-Cepstral-Coefficient 4	Captures the ‘timbre’ or broad ‘texture’ of the voice. It’s the standard way computers distinguish different voice qualities
	Formant Energy (F1)	f1_relative_energy_mean	Average relative energy of the first formant (F1), reflecting the prominence of F1 resonance	The prominence of specific speech resonances. Lower energy variability indicates a more ‘monotonous’ or ‘dull’ resonance
	Formant Energy (F1)	f1_relative_energy_sd	Standard deviation of f1_relative_energy_mean	The prominence of specific speech resonances. Lower energy variability indicates a more ‘monotonous’ or ‘dull’ resonance
	Formant Energy (F2)	f2_relative_energy_mean	Average relative energy of the second formant (F2), reflecting the prominence of F2 resonance	The prominence of specific speech resonances. Lower energy variability indicates a more ‘monotonous’ or ‘dull’ resonance
	Formant Energy (F2)	f2_relative_energy_sd	Standard deviation of f2_relative_energy_mean	The prominence of specific speech resonances. Lower energy variability indicates a more ‘monotonous’ or ‘dull’ resonance
	Formant Energy (F3)	f3_relative_energy_mean	Average relative energy of the third formant (F3), reflecting the prominence of F3 resonance	The prominence of specific speech resonances. Lower energy variability indicates a more ‘monotonous’ or ‘dull’ resonance
	Formant Energy (F3)	f3_relative_energy_sd	Standard deviation of f3_relative_energy_mean	The prominence of specific speech resonances. Lower energy variability indicates a more ‘monotonous’ or ‘dull’ resonance
	Harmonic Differences	h1_a3_harmonic_difference_mean	Mean of the ratio of energy of the first F0 harmonic (H1) to the energy of the highest harmonic in the third formant range (A3)	Indicates voice quality features like ‘breathiness’ or ‘tension’ based on the relationship between vocal harmonics
	Harmonic Differences	h1_a3_harmonic_difference_sd	Standard deviation of h1_a3_harmonic_difference_mean	Indicates voice quality features like ‘breathiness’ or ‘tension’ based on the relationship between vocal harmonics
	Harmonic Differences	h1_h2_harmonic_difference_mean	Mean of the ratio of energy of the first F0 harmonic (h1) to the energy of the highest harmonic in the second formant range (H2)	Indicates voice quality features like ‘breathiness’ or ‘tension’ based on the relationship between vocal harmonics
	Harmonic Differences	h1_h2_harmonic_difference_sd	Standard deviation of h1_h2_harmonic_difference_mean	Indicates voice quality features like ‘breathiness’ or ‘tension’ based on the relationship between vocal harmonics
	Hammarberg Index	hammarberg_index_mean	Ratio of energy between higher (2–5 kHz) and lower (0.5–2 kHz) frequency bands. Parameter to assess the spectral balance of a voice	Measures the drop-off in energy at high frequencies. A high index can sound ‘thin’ or ‘weak’
	Hammarberg Index	hammarberg_index_sd	Standard deviation of Hammarberg Index	Measures the drop-off in energy at high frequencies. A high index can sound ‘thin’ or ‘weak’
	Spectral Slope	spectral_slope_0_500_mean	Mean of the linear regression slope of the logarithmic power spectrum within the two given bands	The ‘tilt’ of the voice energy. A steeper slope often sounds more ‘mellow’ or ‘muffled’
	Spectral Slope	spectral_slope_0_500_sd	Standard deviation of spectral_slope_0_500_mean	The ‘tilt’ of the voice energy. A steeper slope often sounds more ‘mellow’ or ‘muffled’
	Spectral Slope	spectral_slope_500_1500_mean	Mean of the linear regression slope of the logarithmic power spectrum within the two given bands	The ‘tilt’ of the voice energy. A steeper slope often sounds more ‘mellow’ or ‘muffled’
	Spectral Slope	spectral_slope_500_1500_sd	Standard deviation of Slope V500-1500	The ‘tilt’ of the voice energy. A steeper slope often sounds more ‘mellow’ or ‘muffled’
**Syntactic Complexity**				
	Subordinate Clauses	mean_number_subordinate_clauses	Number of subordinate clauses used	Measures sentence complexity. Fewer clauses suggest simpler, more ‘choppy’ sentence structures
	Verb Phrase Complexity	proportion_verb_phrase_with_objects	Proportion of phrases with the verb being directed towards an object (e.g. She ate a delicious meal)	Measures how verbs are used. Simplified verb phrases can be a sign of reduced cognitive effort during speech
	Verb Phrase Complexity	proportion_verb_phrase_with_subjects	Proportion of phrases with the verb being performed by the subject (e.g. She slept peacefully)	Measures how verbs are used. Simplified verb phrases can be a sign of reduced cognitive effort during speech
	Verb Phrase Complexity	verb_phrase_with_aux_and_vp_rate	Ratio of verb phrases that include both auxiliary verbs and main verbs (e.g. She has finished her homework)	Measures how verbs are used. Simplified verb phrases can be a sign of reduced cognitive effort during speech
	Verb Phrase Complexity	verb_phrase_with_aux_rate	Ratio of verb phrases that include an auxiliary verb	Measures how verbs are used. Simplified verb phrases can be a sign of reduced cognitive effort during speech
**Temporal**				
	Total Duration	duration	Length of audio recording	The full length of the audio recording
	Voice-Activity Patterns	length_continuously_unvoiced_regions_mean	Mean of continuously unvoiced regions	Measures the physical segments where vocal folds are vibrating (voiced) versus just air passing (unvoiced, like ‘s’ or ‘h’)
	Voice-Activity Patterns	length_continuously_unvoiced_regions_sd	Standard deviation of length_continuously_unvoiced_regions_mean	Measures the physical segments where vocal folds are vibrating (voiced) versus just air passing (unvoiced, like ‘s’ or ‘h’)
	Voice-Activity Patterns	length_continuously_voiced_regions_mean	Mean of continuously voiced regions	Measures the physical segments where vocal folds are vibrating (voiced) versus just air passing (unvoiced, like ‘s’ or ‘h’)
	Voice-Activity Patterns	length_continuously_voiced_regions_sd	Standard deviation of length_continuously_voiced_regions_mean	Measures the physical segments where vocal folds are vibrating (voiced) versus just air passing (unvoiced, like ‘s’ or ‘h’)
	Pause Frequency	number_of_pauses	Number of pauses in between speech segments based on speech intervals. Pauses were identified via Silero VAD, utilizing a 0.5 speech probability threshold and a minimum silence duration of 100ms. ^54^	How often the speaker stops. Frequent pauses make speech sound hesitant or fragmented
	Pause Length	pause_durations_mean	Mean length of pauses	The actual time spent in silence
	Pause Length	pause_durations_sd	Standard deviation of pause duration	The actual time spent in silence
	Pause Length	pause_durations_sum	Sum of pause lengths over whole utterance	The actual time spent in silence
	Pause Frequency	pause_rate	Frequency of pauses within a spoken utterance	How often the speaker stops. Frequent pauses make speech sound hesitant or fragmented
	Speech-to-Silence Ratio	speech_ratio	Ratio of utterance to duration	Percentage of the total time spent actually talking. A low ratio means the recording is dominated by silence
	Utterance Length	utterance_durations_mean	Mean length of utterances	The length of continuous speech segments between pauses. Shorter segments indicate ‘staccato’ or effortful speech
	Utterance Length	utterance_durations_sd	Standard deviation of utterance durations	The length of continuous speech segments between pauses. Shorter segments indicate ‘staccato’ or effortful speech
	Utterance Length	utterance_durations_sum	Sum of utterance durations	The length of continuous speech segments between pauses. Shorter segments indicate ‘staccato’ or effortful speech
**Word Types**				
	Parts of Speech Proportion	adjective_rate	Relative frequency (ratio to total words spoken) of adjectives used	The proportion of different word types (e.g., more nouns vs. more verbs). Changes can reflect shifted focus or cognitive style
		adposition_rate	Ratio of adpositions used	
		adverb_rate	Ratio of adverbs used	
		conjunction_rate	Ratio of conjunctions used	
		determiner_rate	Ratio of determiners (articles, demonstratives, possessives) used	
		inflected_verb_rate	Ratio of inflected verbs used	
		noun_rate	Ratio of nouns used	
		pronoun_rate	Ratio of pronouns used	
		proper_noun_rate	Ratio of proper nouns used	
		verb_rate	Ratio of verbs used	

H2: We hypothesize that the identified speech features will show a significant and consistent relationship with depression severity (BDI-II scores) across the pooled sample. By controlling for ‘site’ as a covariate, we aim to demonstrate that these associations reflect underlying pathology rather than technical or environmental variability between recording locations.

H3: We hypothesize that a machine learning model trained on the Aachen cohort will successfully generalize to the independent Oldenburg cohort, yielding classification performance (ROC-AUC) above the chance level of 0.5. This would demonstrate that the learned speech representations are robust to site-specific differences in participant demographics and recording conditions.

## Methods

### Participants

#### Cohort 1 (Aachen sample)

Participants were involved in a study focused on exploring unconscious emotional conflicts in individuals diagnosed with MDD [38]. The study was approved by the ethics committee of the University Hospital Aachen, Germany (Ethik-Kommission an der Medizinischen Fakultät der RWTH Aachen; reference number EK 045 − 19). It was preregistered at the open science framework (https://osf.io/37xd2/*).* All study activities followed the principles of the Declaration of Helsinki. Written informed consent was secured from every participant before the study began. Each participant received a payment of 45 Euros.

Patients with a diagnosis of MDD were recruited from the Department of Psychiatry, Psychotherapy, and Psychosomatics at RWTH Aachen University in Germany. The cohort included inpatients as well as former inpatients who agreed to participate in other studies. Most patients (67.5%) were currently treated with anti-depressants. Among the patients, 22.5% were treated with selective serotonin reuptake inhibitors (SSRI), 17.5% with serotonin-norepinephrine reuptake inhibitors (SNRI), and 15.0% with noradrenergic and specific serotonergic antidepressants (NaSSA). Additionally, 12.5% received a combination of multiple antidepressant classes, most commonly NaSSA + SSRI (10.0%) and NaSSA + SNRI (2.5%). The patient cohort was free from benzodiazepines, anxiolytics, and other sedative medications at the time of the study. A control group of healthy individuals (HCs), matched by age and gender, was assembled through local advertisements and flyers distributed in the Aachen area. Demographic details were gathered via a questionnaire.

Inclusion criteria required participants to either have a diagnosis of MDD based on DSM-V criteria or meet the criteria for HC. To be classified as HC, clinical interview data and questionnaire scores were assessed. Potential HC showing signs of clinically significant psychiatric or somatic conditions, as well as any conditions that might confound speech production or cognitive performance, were excluded from further participation and advised to seek appropriate care. This included, but was not limited to, developmental or functional disorders affecting voice and speech, such as reading disabilities, speech delays, or localized vocal cord issues. Additional requirements for all participants (HC and MDD) included being 18–55 years old, fluent in German, and right-handed. Exclusion criteria for all participants included the inability to comply with the study protocol, the presence of metallic implants or foreign bodies, and any chronic or acute illness or psychiatric disorder (according to DSM-V) that may affect cognitive functioning.

#### Cohort 2 (Oldenburg sample)

In the second cohort, participants took part in the so-called PsyApp study examining the utility of speech analysis to detect symptom severity in a psychiatric sample. Outpatients and inpatients with MDD were recruited from the Department of Psychiatry, University of Oldenburg, Germany. All patients underwent treatment as usual. In this cohort, 27.3% of the patients were currently treated with antidepressants. Among the patients, 4.5% were treated with SSRI, 13.6% with SNRI, and 4.5% NaSSA. Additionally, 4.5% received a combination of multiple antidepressant classes (NaSSA + SNRI). The patient cohort was free from benzodiazepines, anxiolytics, and other sedative medications at the time of the study. Recruitment of the HC was carried out through posted notices, social media, and public press releases from the University of Oldenburg.

The study was approved by the ethics committee of the School of Medicine and Health Sciences, Carl von Ossietzky University of Oldenburg, Germany (Ethikkommission der Fakultät für Medizin- und Gesundheitswissenschaften, University of Oldenburg, Germany, ethical vote number 2023-066). All study activities followed the principles of the Declaration of Helsinki. Written informed consent was secured from every participant before the study began. Healthy controls received a payment of 100 Euros for their participation in the study.

Participants were required to be 18–65 years old, able to give written consent, have sufficient German language skills, and be diagnosed with MDD. Exclusion criteria included lack of consent capacity, acute suicidality or psychosis, being a non-native German speaker or having significant hearing or speech problems, benzodiazepine use, severe or unstable physical illnesses, cognitive, linguistic, or neurological deficits, a history likely to affect brain anatomy, need for incompatible treatments or medications, current substance abuse or dependence, or a diagnosis characterized by emotional blunting (e.g., psychosis).

### Clinical assessment

In both cohorts, diagnoses and all clinical assessments were conducted by trained psychologists and psychiatrists. Diagnoses were based on the DSM-V [39]. The severity of depressive symptoms was assessed based on self-reports using the BDI-II [40].

### Procedure

The data of the Aachen cohort were collected over a period of 18 months at the Department of Psychiatry, Psychotherapy, Psychosomatics, Medical Faculty RWTH Aachen University. The participants underwent a series of neuropsychological tests and psychopathological rating scales before performing two tasks in a 3-tesla MRI scanner while simultaneously recording electroencephalography, concluding with the speech task. For a detailed description on the findings on MRI and speech paradigms refer to Schräder et al. 2024 [38] and Menne et al. 2024 [15].

The Oldenburg data were collected over a period of 18 months at the Department of Psychiatry, University of Oldenburg, Germany. After the extensive clinical assessment, participants conducted speech tasks as described below as well as physiological measures (EEG, heart rate variability).

### Speech assessment and processing

At both sites, recordings took place in a consistent, quiet clinical room where the tablet was placed on a desk in front of the participant. While the general positioning was standardized, the exact distance from the participant’s mouth to the internal microphone was not strictly fixed, allowing for minimal natural variations consistent with real-world speech capture settings. In the Aachen cohort, speech was captured using an Apple iPad Pro 2020 (4th Generation). Participants were prompted to narrate a positive and a negative event in their lives, a paradigm used in previous studies to elicit emotion in speech [15, 41, 42]. This task was chosen specifically because it encourages spontaneous, emotionally rich speech linked to mood and affective state. Furthermore, the standardized instructions (“Can you tell me in one minute about a positive/negative event in your life?”; German original: “Bitte erzählen Sie mir über ein positives Ereignis in Ihrem Leben.”) provide a consistent framework while allowing natural expression, balancing experimental control with ecological validity. Autobiographical narratives like this have demonstrated utility in prior research as a robust and replicable method for capturing clinically relevant vocal markers [15, 43].

The instructions were pre-recorded by a psychologist and played from the tablet, ensuring consistent instructions across both experiments. The responses were recorded using the tablet’s internal microphone. The same procedure was executed in the Oldenburg cohort, all recordings were conducted using a Samsung Galaxy Tab S7. However, participants additionally told a neutral story. At this site, further speech assessments were conducted such as the Speech Biomarker-Cognition (SB-C [44]) consisting of a semantic verbal fluency and a verbal learning test, a diadochokinetic task [45] and a sustained phonation task [46]. Also, participants were asked to describe a picture, the so-called “Cookie Theft” picture from the Boston Diagnostic Aphasia Examination (BDAE [47]). The data presented here is based only on the tasks used in both studies, i.e., the positive and negative storytelling.

### Statistical analyses

To ensure the robustness of the identified biomarkers, we employed a dual-track analytic strategy. Group-level comparisons and correlations were performed on the pooled sample using Site as a covariate to identify site-agnostic features. In contrast, the machine learning classification followed a strict cross-site validation protocol, where models were trained on one cohort and tested on the other, ensuring that predictive performance reflects true generalizability to independent clinical settings.

Speech attributes were categorized into groups, sorted distinctly by negative and positive story. Categories consisted of features pertaining to energy, frequency, lexical richness, sentiment, spectral attributes, syntactic complexity, temporal properties, and word types (refer to Table 1 for groupings and corresponding attributes). The selection of the speech features was based on those previously associated with symptoms of depression, such as length and number of pauses, jitter, shimmer, pitch, loudness, and others [15, 16, 48–52]. In our previous work [15], we identified a set of acoustic and linguistic features that significantly differed between healthy controls and MDD participants and were associated with BDI-II scores, such as lexical richness and speech sentiment, among others. In the current analysis, we aimed to examine whether similar patterns are observed in an independent cohort from Oldenburg. To this end, we employed linear models including group (Aachen vs. Oldenburg), diagnosis (HC vs. MDD) as predictors, while controlling for age, education, and sex. The dependent variable in each model was the individual feature score. To specifically assess cohort effects, we conducted two additional analyses. First, we extended the original group comparison model by including an interaction between diagnosis (MDD vs. HC) and location (Aachen vs. Oldenburg), again controlling for age, education, and sex: feature ~ diagnosis + location + age + education + sex + diagnosis*location. We tested the interaction term to identify features where diagnostic effects differed by location, reporting means ± standard deviation (SD) per group and site, along with the t-value and corresponding *p*-value of the interaction term, and ΔR² (change in R² compared to a base model without the interaction). Second, we tested whether location moderated the relationship between speech features and BDI-II scores using: BDI ~ speech_feature + location + speech_feature*location. As above, we reported the interaction’s t-value and corresponding *p*-value, and ΔR². These models tested whether diagnostic or predictive effects varied by sample.

Furthermore, using partial Spearman correlations, we correlated speech features with the BDI-II controlling for cohort, age, and education, resulting in those features significantly correlated regardless of origin (i.e., features were significant across cohorts). To further examine the relationship between depression symptom dimensions and speech, we tested associations between BDI-II factors, derived from several established models summarized by Kim and Lee (2024) [53, 53], and clustered categories of these features as displayed in Table 1. To compute composite scores per category, first all features of the category were z-standardized over the full sample. Then mean scores per category were computed and these mean scores were correlated with the BDI-II factors. This approach allowed us to assess whether specific symptom clusters (e.g., affective, cognitive, somatic) relate more strongly to particular domains aspects of speech. All BDI-II factors tested are listed in Suppl. Table S1.

Additionally, in our previous publication we employed an ML-based approach creating three different models to differentiate between HC and MDD individuals. These included (1) baseline demographic data (age, sex, and years of education as well as results from neuropsychological testing), (2) the BDI-II, and (3) a speech model consisting of features identified by ML. The best performing speech model consisted of 10 features, 7 of which were selected over all leave-one-out cross validation iterations (rate of loudness peaks negative storytelling condition, vocal tremor negative condition, minimum pitch negative condition, average Mel Frequency Cepstral Coefficient (MFCC)1 positive condition, shimmer positive condition, H1-A3 mean harmonic difference positive condition, minimum pitch positive condition). For the work presented here, we used the same approach, whereas the Aachen cohort functioned as a training set and the Oldenburg cohort as a test set. As the clinical assessments differed between cohorts, the newly created (1) baseline model consisted of age, sex, and years of education. Again, we included a model consisting solely of (2) BDI-II scores, as well as (3) speech models where we applied feature selection based on mutual information. Additionally, to account for sample differences in age and education, we added two further models where we regressed out the effects of age and education of the speech features using a linear regression fitted on the train set. These two models consisted in one model using only the residuals of the features and one model using the residuals and the BDI score. To prevent data leakage, this feature selection was performed strictly within the training pipeline on the Aachen cohort. No information from the Oldenburg test set was used to inform feature choice or model tuning. For all sets of predictors (1), (2) and (3), we used differing ML approaches (RF=Random Forest, SVM=Support Vector Machine, DT=Decision Trees, LM=Linear Model, XT=Extra Trees) and selected the model which performed best based on AUC-ROC. All performance metrics are reported with a 95% CI based on a bootstrap distribution over 1000 iterations.

To compute the acoustic and linguistic features, the extraction scripts were implemented in Python 3.12.7, based on ki: elements’ “Sigma” speech processing library. All statistical analyses were conducted using scipy v1.13.1 and pingouin v0.5.5. The ML was implemented using sklearn v1.5.1. The extraction code is available upon reasonable request. The feature extraction process analyzes two distinct dimensions of the signal: its acoustic properties and its lexical-semantic content. For the acoustic analysis, Silero VAD (Voice Activity Detection) [54] is first applied to each file to isolate active speech segments from silence and background noise, enabling the precise computation of acoustic markers such as pitch and intensity exclusively from voiced regions. Simultaneously, the audio is processed via Google’s Automatic Speech Recognition (ASR) to generate a transcript that serves as the foundation for linguistic analysis. By leveraging high-performance language models, specifically Stanza [55] for structural parsing and lemmatization and Flair [56] for advanced word embeddings and entity recognition, the system extracts deep syntactic and semantic features from the spoken text.

The significance level (α) was set at 0.05. All *p*-values reported were adjusted for multiple hypothesis testing using the Benjamini-Hochberg procedure [57]. As in our previous work [15], features were grouped into categories as presented in Table 1, and p-values were adjusted separately for each category to account for multiple comparisons.

## Results

### Demographics

A total of 135 participants (*n* = 71 HC, *n* = 64 MDD) were included across both sites. Participants in Aachen were significantly younger than in Oldenburg for both HC and MDD groups. Also, individuals with MDD differed between sites regarding years of education and BDI-II scores (see Table 2 for detailed overview). Due to these differences, all analyses were corrected for age and years of education.

**Table 2:** Demographic and clinical scores

	Aachen HC	Oldenburg HC	Aachen MDD	Oldenburg MDD	*p* value Aachen HC vs. Aachen MDD	*p* value Oldenburg HC vs. Oldenburg MDD	*p* value Aachen HC vs. Oldenburg HC	*p* value Aachen MDD vs. Oldenburg MDD
***n***	52	19	44	20				
**Sex**	F: 63.5%	F: 42.1%	F: 56.8%	F: 40.0%	0.84	1.0	0.18	0.43
**Age**	26.17 (7.08)	42.11 (11.24)	26.14 (6.19)	37.53 (13.85)	0.97	0.28	**< 0.001**	**0.002**
**Years of education**	14.06 (2.24)	13.12 (2.06)	12.39 (2.0)	11.06 (1.63)	**0.002**	**< 0.02**	0.24	**0.04**
**BDI-II**	3.94 (3.13)	2.72 (2.52)	26.37 (10.39)	16.47 (14.89)	**< 0.001**	**0.001**	0.17	**0.002**

The values are given as the means ± standard deviations in brackets. Gender shares are given as percentages. The nonparametric Kruskal-Wallis H test was used to determine group differences, for sex differences a Χ^2^ test of independence was conducted. BDI-II=Beck Depression Scale. Bold values indicate statistically significant results (p < 0.05)

### Group differences in speech features between HCs and MDD patients (combined cohorts)

Several speech features showed significant differences between HC and individuals with MDD (see Table 3). The most prominent effects were observed in temporal features, where MDD participants consistently showed higher values than healthy controls. For instance, the total duration of utterances was markedly longer in the MDD group for both positive (HC: 10.20s; MDD: 20.79s; adj. *p* = 0.002) and negative stories (HC: 11.84s; MDD: 30.88s; adj. *p* = 0.002). The total pause duration in the negative story (HC: 17.17s; MDD: 46.96s; adj. *p* = 0.002) and the positive story (HC: 14.36s; MDD: 30.74s; adj. *p* = 0.003) was similarly increased in the MDD group. Furthermore, the number of pauses was significantly higher in the MDD groups for both negative (HC: 79.68; MDD: 187.97; adj. *p* = 0.002) and positive story (HC: 67.68; MDD: 124.83; adj. *p* = 0.003).

**Table 3:** Group differences as assessed by linear models controlling for age, education, sex and sample effects between healthy controls and depressed patients for both cohorts

Speech feature	HC mean ± SD	MDD mean ± SD	t-Value	*p*	β	adj. *p*
**average_mfccs_1_pos**	90.507 ± 13.698	101.896 ± 12.369	4.628	0	0.367	0.001
**type_token_ratio_neg**	0.754 ± 0.129	0.65 ± 0.177	-4.357	0	-0.382	0.002
**duration_neg**	30.85 ± 19.955	80.618 ± 104.188	4.174	0	0.374	0.002
**utterance_durations_sum_neg**	11.837 ± 8.204	30.884 ± 37.509	4.194	0	0.373	0.002
**utterance_durations_sum_pos**	10.203 ± 11.137	20.788 ± 21.591	3.924	0	0.33	0.003
**duration_pos**	26.06 ± 22.528	53.538 ± 59.593	4.012	0	0.347	0.003
**number_of_pauses_neg**	79.676 ± 52.335	187.969 ± 241.663	3.929	0	0.355	0.003
**word_count_neg**	72.211 ± 51.774	175.297 ± 231.757	4.012	0	0.361	0.003
**number_of_pauses_pos**	67.676 ± 54.912	124.828 ± 129.681	3.883	0	0.339	0.003
**pause_durations_sum_neg**	17.166 ± 12.951	46.958 ± 67.061	3.995	0	0.361	0.003
**pause_durations_sum_pos**	14.363 ± 12.544	30.742 ± 39.002	3.773	0	0.333	0.004
**word_count_pos**	63.817 ± 60.588	116.781 ± 135.506	3.566	0.001	0.313	0.006
**average_mfccs_1_neg**	90.723 ± 13.921	99.479 ± 11.582	3.597	0	0.292	0.006
**average_mfccs_3_neg**	18.33 ± 6.707	23.58 ± 7.896	3.524	0.001	0.271	0.007
**average_mfccs_3_pos**	17.498 ± 7.27	22.085 ± 6.558	3.331	0.001	0.255	0.011
**type_token_ratio_pos**	0.774 ± 0.132	0.703 ± 0.16	-3.35	0.001	-0.292	0.011
**f2_relative_energy_sd_pos**	9.598 ± 1.228	8.847 ± 1.059	-3.08	0.003	-0.261	0.024
**utterance_durations_mean_pos**	0.141 ± 0.04	0.17 ± 0.049	2.971	0.004	0.212	0.032
**average_mfccs_4_neg**	3.467 ± 6.619	8.00 ± 9.071	2.87	0.005	0.201	0.039
**utterance_durations_mean_neg**	0.145 ± 0.041	0.169 ± 0.045	2.871	0.005	0.199	0.039

Variables are listed in descending order for adjusted *p* value. pos=positive story; neg=negative story. T = t-value of predictor in linear model; β = normalized beta coefficient; Adj. *p* = *p* value adjusted for multiple hypothesis testing according to the Benjamini-Hochberg procedure. For brevity, only features with adj. *p* < 0.05 are reported here. The full list can be found in Suppl. Table S2

In the spectral domain of speech features, significant differences were observed in several MFCCs, with higher values in the MDD group. The MFCC1 was elevated in MDD participants for both positive (HC: 90.51; MDD: 101.90; adj. *p* = 0.002) and negative stories (HC: 90.72; MDD: 99.48; adj. *p* = 0.011), as was the MFCC3 in positive (HC: 17.50; MDD: 22.09; adj. *p* = 0.016) and negative stories (HC: 18.33; MDD: 23.58; adj. *p* = 0.008).

For lexical richness features, MDD participants produced a lower type-token ratio in both the negative (HC: 0.75; MDD: 0.65; adj. *p* = 0.002) and positive story (HC: 0.77; MDD: 0.70; adj. *p* = 0.011) indicating reduced lexical diversity. The number of words used was also higher in both the negative (HC: 72.21; MDD: 175.30; adj. *p* = 0.002) and the positive story (HC: 63.82; MDD: 116.78; adj. *p* = 0.007) for MDD participants.

Several speech features showed significant interactions between groups (MDD vs. HC) and location (Aachen vs. Oldenburg). Shimmer features (the cycle-to-cycle variation in vocal amplitude perceived as voice crackling or instability) from the negative storytelling condition (*t*-values between − 4.59 and − 3.72, adj. *p* < 0.002 for all) showed significant interactions, with MDD patients exhibiting higher shimmer values in Aachen compared to HC, and the opposite pattern in Oldenburg. Several measures of vocal quality exhibited significant site-dependent effects. Specifically, significant interaction effects were observed for shimmer in the positive condition (t = -3.05, adj. *p* < 0.05) and various jitter metrics in both storytelling conditions (all adj. *p* < 0.05). In these cases, the Aachen cohort showed higher perturbation values in MDD patients relative to HC, whereas the Oldenburg cohort exhibited the reverse pattern. Similarly, significant interactions were found for the Harmonics-to-Noise Ratio (HNR SD) and MFCC1 across both emotional conditions (all adj. *p* < 0.05), where the direction of the association with MDD shifted between the two study sites Significant results are displayed in Table 4, a comprehensive list with all results can be found in Supplementary Table S3.

**Table 4:** Results of moderation analysis assessing the interaction between diagnosis (MDD vs. HC) and location (Aachen vs. Oldenburg) in relation to speech features

	Mean HC Aachen± SD	Mean MDD Aachen ± SD	Mean HC Oldenburg± SD	Mean MDD Oldenburg ± SD	t-Value	ΔR²	adj. *p*
**local_dB_shimmer_neg**	121.34 ± 17.705	135.223 ± 18.814	150.691 ± 12.964	136.358 ± 19.5	-4.326	0.098	0.001
**apq5_shimmer_neg**	7.368 ± 1.805	9.286 ± 2.355	10.709 ± 1.536	9.352 ± 1.975	-4.401	0.099	0.001
**apq3_shimmer_neg**	5.504 ± 1.228	6.766 ± 1.71	7.611 ± 1.113	6.475 ± 1.371	-4.588	0.121	0.001
**local_shimmer_neg**	12.704 ± 2.397	14.657 ± 2.691	16.89 ± 1.949	14.693 ± 2.633	-4.585	0.107	0.001
**average_mfccs_1_pos**	85.245 ± 10.606	101.797 ± 13.635	104.906 ± 10.59	102.115 ± 9.298	-4.316	0.086	0.001
**dda_shimmer_neg**	16.513 ± 3.684	20.297 ± 5.131	22.834 ± 3.338	19.424 ± 4.112	-4.588	0.121	0.001
**hnr_sd_neg**	6.333 ± 0.704	5.741 ± 0.898	5.33 ± 0.507	5.934 ± 1.148	4.031	0.088	0.002
**apq11_shimmer_neg**	12.001 ± 4.03	14.969 ± 3.645	17.58 ± 3.501	15.266 ± 2.813	-3.716	0.077	0.005
**hnr_sd_pos**	6.451 ± 0.772	5.741 ± 0.72	5.513 ± 0.872	5.814 ± 0.879	3.7	0.07	0.005
**average_mfccs_1_neg**	86.286 ± 12.044	99.388 ± 13.039	102.869 ± 11.403	99.682 ± 7.743	-3.743	0.07	0.005
**ppq5_jitter_neg**	1.156 ± 0.321	1.404 ± 0.306	1.745 ± 0.428	1.56 ± 0.379	-3.347	0.054	0.016
**ddp_jitter_neg**	3.109 ± 0.891	3.871 ± 1.018	4.591 ± 1.129	4.177 ± 1.079	-3.092	0.052	0.031
**rap_jitter_neg**	1.036 ± 0.297	1.29 ± 0.339	1.53 ± 0.376	1.392 ± 0.36	-3.092	0.052	0.031
**local_dB_shimmer_pos**	122.215 ± 14.258	134.412 ± 19.876	148.427 ± 12.297	141.869 ± 15.068	-3.054	0.05	0.032

The model was built with the formula: feature ~ diagnosis + location + age + education + sex + diagnosis*location, with age, sex, and education included as covariates to control for their effects. The table reports the mean ± SD for each diagnosis and location group, along with the *t*-value and corresponding p-value for the interaction term. The change in R-squared (ΔR²) between the interaction model and a model without the interaction term is also provided to estimate the effect size of the interaction. For brevity, only features with adj. *p* < 0.05 are reported here. The full list can be found in Suppl. Table S3

To ensure identified markers were not artifacts of medication-induced xerostomia, which can alter oral acoustic impedance and spectral characteristics, we conducted subgroup analyses within both cohorts; no significant differences in speech features were found between medicated and unmedicated patients (*p* > 0.05 for all features).

### Correlation of speech features with BDI-II scores (combined cohorts)

**Fig. 1:**
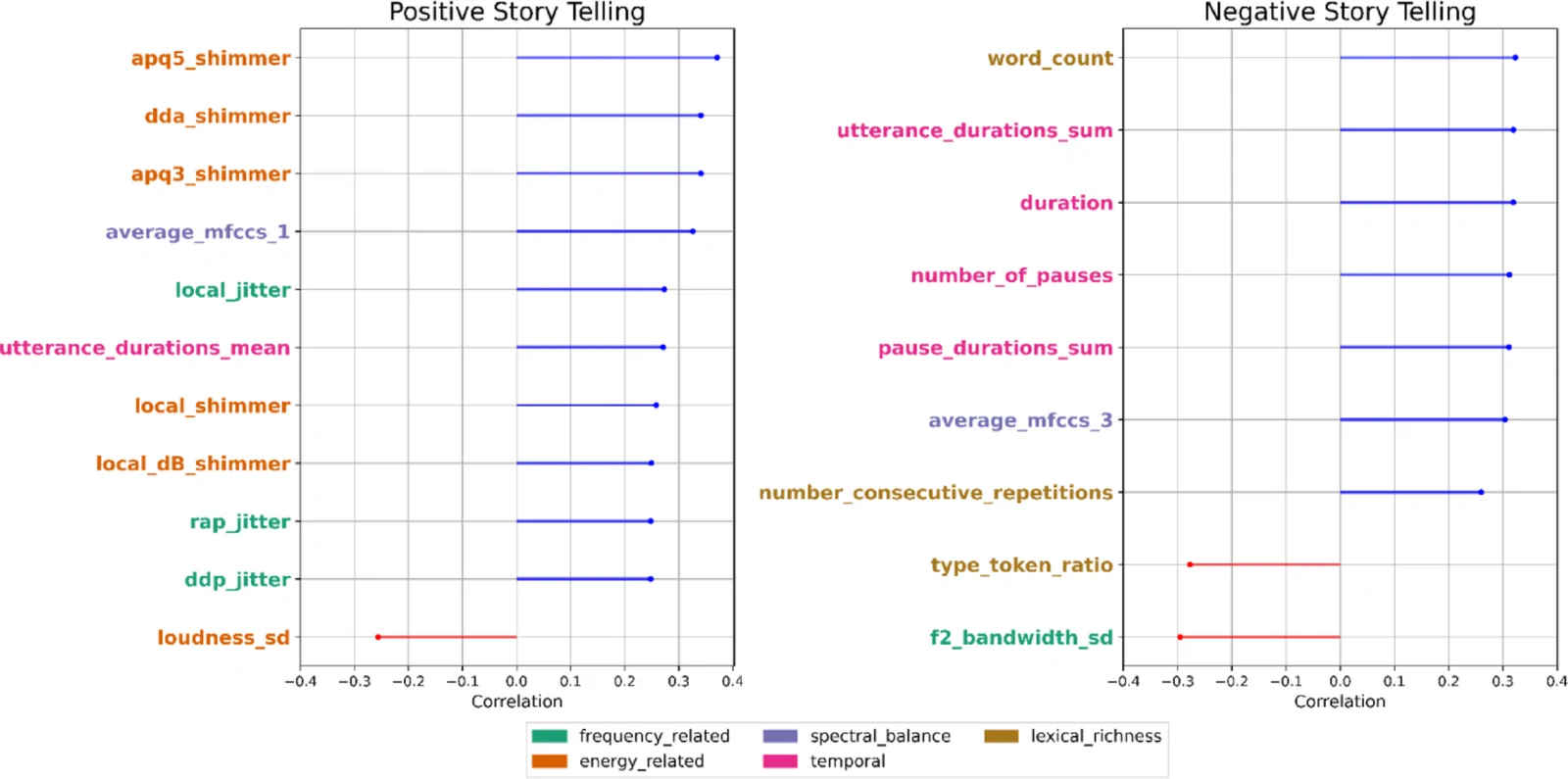
Lollipop plots depicting results of partial correlations (*r*) between speech features and the BDI-II, controlling for groups (Aachen vs. Oldenburg), indicating meaningful features in both cohorts. Variables are colour-coded according to the captions, based on the categories defined in Table 1. For brevity, only features with adj. *p* < 0.05 are depicted here. All results including effect sizes and *p* values can be found in Supp. Table S4

We computed partial correlations between speech features and depression severity, as measured by the BDI-II, while controlling for sites (Aachen vs. Oldenburg), indicating meaningful features in both cohorts (Fig. 1; Table 5). Several features showed significant associations with BDI-II scores after correction for multiple comparisons, spanning multiple speech feature categories.

**Table 5:** Partial correlations (*r)* between speech features and the BDI-II

Speech feature	*r*	adj. *p*
**utterance_durations_sum_neg**	0.32	< 0.001
**duration_neg**	0.32	< 0.001
**word_count_neg**	0.32	< 0.001
**average_mfccs_1_pos**	0.33	< 0.001
**dda_shimmer_pos**	0.34	< 0.001
**apq3_shimmer_pos**	0.34	< 0.001
**apq5_shimmer_pos**	0.37	< 0.001
**f2_bandwidth_sd_neg**	-0.30	0.015
**average_mfccs_3_neg**	0.30	0.015
**pause_durations_sum_neg**	0.31	0.015
**number_of_pauses_neg**	0.31	0.015
**type_token_ratio_neg**	-0.28	0.027
**utterance_durations_mean_pos**	0.27	0.035
**local_jitter_pos**	0.27	0.035
**local_shimmer_pos**	0.26	0.041
**number_consecutive_repetitions_neg**	0.26	0.041
**loudness_sd_pos**	-0.26	0.048
**rap_jitter_pos**	0.25	0.049
**ddp_jitter_pos**	0.25	0.049
**local_dB_shimmer_pos**	0.25	0.049

Pos=positive story; neg=negative story; *r*=correlation coefficient; adj. *p* = *p* value adjusted for multiple hypothesis testing according to the Benjamini-Hochberg procedure. For brevity, only features with adj. *p* < 0.05 are reported here. The full list can be found in Suppl. Table S4

In the temporal domain, longer utterances in the negative (*r* = 0.32, adj. *p* < 0.001) and positive storytelling task (*r* = 0.22, adj. *p* < 0.05) were positively associated with higher BDI-II scores. Additionally, longer pauses (*r* = 0.31, adj. *p* = 0.015) and the number of pauses (*r* = 0.31, adj. *p* = 0.015) in the negative story were significantly correlated with higher depression scores.

Spectral features showed robust associations, with the MFCC1 (*r* = 0.33, adj. *p* < 0.001) and MFCC3 (*r* = 0.30, adj. *p* < 0.02) correlating positively with BDI-II scores.

In the energy-related features, shimmer measures were significantly associated (r between 0.34 and 0.37, *p* < 0.001, respectively) with BDI-II scores. Furthermore, jitter features showed significant associations (r between 0.25 and 0.27, adj. *p* < 0.05, respectively), suggesting altered voice quality characteristics in individuals with higher depression severity. A higher BDI-II score was significantly associated with a lower loudness variability in the positive story (*r* = -0.26, adj. *p* < 0.05).

Within the frequency domain, a significant negative association was observed for the F2 bandwidth SD in the negative condition (*r* = -0.30, adj. *p* < 0.02), pointing to reduced formant bandwidth variability in the speech of participants with higher depression scores.

Lastly, lexical features showed significant effects. Higher word counts in the negative story correlated with increased BDI-II scores (*r* = 0.32, adj. *p* < 0.001), while the type token ratio (*r* = -0.28, adj. *p* < 0.03) decreased with increasing BDI scores, indicating lower lexical richness in participants reporting more severe depressive symptoms. Moreover, a higher number of consecutive repetitions was positively associated with increased BDI-II scores (*r* = 0.26, adj. *p* < 0.05).

When examining whether location moderated the relationship between speech features and BDI-II scores, significant interactions were found for several features, particularly from the negative storytelling condition. Shimmer features showed negative correlations in Oldenburg (ranging from − 0.47 to -0.03, adj. *p* < 0.05) and positive correlations in Aachen (ranging from 0.29 to 0.37, *p* < 0.05). Jitter features from Oldenburg showed lower and positive correlation coefficients (*r* = -0.19, adj. *p* < 0.05) compared to Aachen (*r* = 0.37, adj. *p* < 0.05). Utterance duration SD from the negative storytelling condition had a significant interaction, with Aachen participants showing a stronger positive correlation (*r* = 0.41) and Oldenburg a negative correlation (*r* = -0.31, adj. *p* = 0.001) with the BDI-II. Similarly, utterance duration SD from the positive storytelling condition showed opposite patterns between Aachen and Oldenburg (adj. *p* < 0.05). Mean HNR from the negative storytelling condition showed a significant interaction, with Aachen individuals showing a positive correlation (*r* = 0.46, adj. *p* < 0.05) and Oldenburg a negative correlation (*r* = -0.22, adj. *p* < 0.05). Word frequency mean from the negative storytelling condition also showed a significant interaction, with Aachen participants showing a positive correlation (*r* = 0.131, adj. *p* < 0.05) and Oldenburg a weak negative correlation (*r* = -0.002, adj. *p* < 0.05). Finally, rate of loudness peaks from the positive storytelling condition showed opposite patterns between Aachen (*r* = -0.26, adj. *p* < 0.05) and Oldenburg (*r* = 0.17, adj. *p* < 0.05). Significant interactions are displayed in Table 6, a comprehensive list with all results can be found in Supplementary Table S5.

**Table 6:** Results from the moderation analysis assessing location as a moderator in the relationship between speech features and BDI-II scores

Speech feature	*r* Oldenburg	*r* Aachen	t-value	ΔR²	adj. *p*
**utterance_durations_sd_pos**	-0.20	0.50	-5.04	0.16	< 0.001
**utterance_durations_sd_neg**	-0.31	0.41	-4.73	0.15	0.001
**dda_shimmer_neg**	-0.43	0.30	-3.73	0.10	0.01
**apq11_shimmer_pos**	-0.03	0.33	-3.74	0.09	0.01
**local_dB_shimmer_neg**	-0.41	0.30	-3.69	0.10	0.01
**apq3_shimmer_neg**	-0.43	0.30	-3.73	0.10	0.01
**local_shimmer_neg**	-0.47	0.29	-3.57	0.09	< 0.02
**hnr_mean_neg**	0.46	-0.22	3.37	0.08	< 0.05
**apq5_shimmer_neg**	-0.39	0.37	-3.25	0.07	< 0.05
**ddp_jitter_neg**	-0.19	0.37	-3.09	0.07	< 0.05
**utterance_durations_mean_neg**	-0.23	0.43	-3.18	0.07	< 0.05
**rap_jitter_neg**	-0.19	0.37	-3.09	0.07	< 0.05
**word_frequency_mean_neg**	0.00	0.13	-3.12	0.07	< 0.05
**rate_loudness_peaks_pos**	0.17	-0.26	3.12	0.07	< 0.05

The model tested the interaction between each speech feature and location (Aachen vs. Oldenburg) using the formula: BDI ~ Speech_feature + location + speech_feature*location. The table reports the t-value and corresponding p-value for the interaction term, as well as the change in R-squared (ΔR²) between the interaction model and a model without the interaction term to estimate the effect size of the interaction. For brevity, only features with adj. *p* < 0.05 are reported here. The full list can be found in Suppl. Table S5

Correlations between BDI-II factors and speech domains resulted in significant correlations between the “affective” factor (comprising BDI-II items 4, 10, 12, and 13, as proposed by [58] and temporal speech features derived from negative (*r* = 0.30, adj. *p* < 0.01) and positive (*r* = 0.26, adj. *p* < 0.05) storytelling. Other speech domains correlated with the affective factor were below the significance threshold (adj. *p* > 0.30). Similarly, no significant correlations were observed between other BDI-II factors from various models (up to three dimensions; see Suppl. Table S1) and speech domains. Notably, temporal speech domains, followed by energy-related features, consistently showed the highest correlations with *p*-values closest to statistical significance. Full correlation results for all BDI-II factors with speech domains are provided in Suppl. Table S1.

### Machine learning model performance

**Fig. 2:**
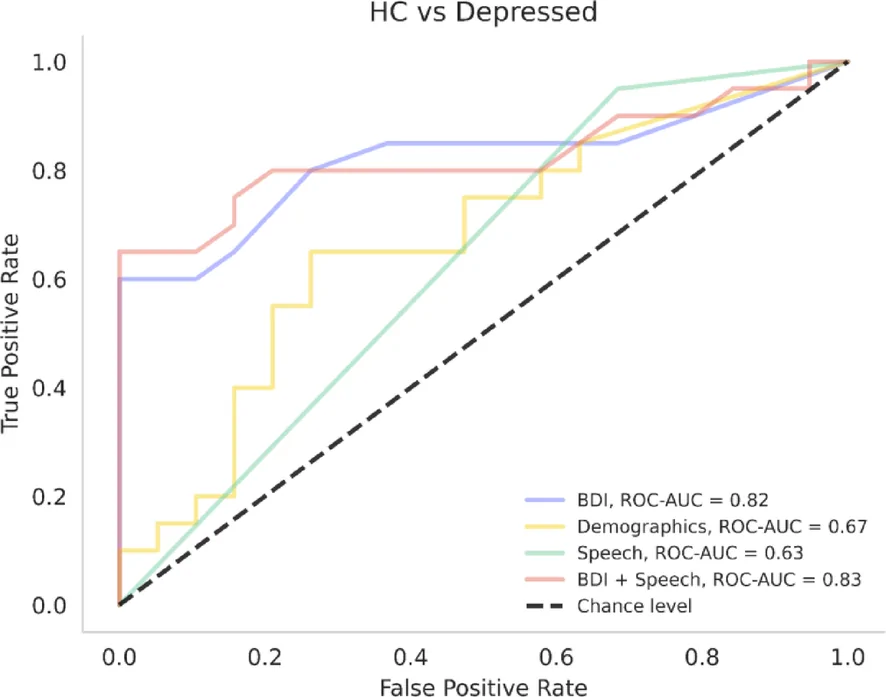
Receiver operating characteristic curves and areas under the curve (ROC-AUC) for healthy controls (HC) vs. Major Depressive Disorder (MDD) patients classification models. Training was executed on the Aachen cohort, testing on the Oldenburg cohort. Legend: BDI=Support Vector Machine (SVM) model with Beck Depression Inventory-II (BDI-II) as the only variable; Demographics=linear model consisting of demographic and clinical data; HC=healthy controls; Speech=Decision Tree (DT) speech model consisting of 20 features

To evaluate the generalizability of our speech-based markers, we applied a cross-site validation framework training on the Aachen cohort and testing on the independent Oldenburg dataset (*n* = 39, Fig. 2). We report 95% Confidence Intervals (CIs) for all metrics via bootstrapping (1,000 iterations); full details are provided in Table 7.

**Table 7:** Performance metrics for different classification models

Comparison	Model	k features selected	BA(95% CI)	ROC-AUC (95% CI)	Sensitivity (95% CI)	Specificity (95% CI)
Baseline model (age, sex, years of education)	Extra Trees (XT)	3	0.693 (0.544–0.845)	0.672 (0.500–0.843)	0.650 (0.438–0.842)	0.737 (0.533–0.933)
BDI-II model	Linear model (LM)	1	0.775 (0.667–0.882)	0.820 (0.668–0.940)	0.550 (0.333–0.765)	1.000 (1.000–1.000)
Speech model	Decision Trees (DT)	20	0.633 (0.519–0.750)	0.633 (0.519–0.750)	0.950 (0.842–1.000)	0.316 (0.118–0.526)
Speech model, features corrected for age/education	Decision Trees (DT)	20	0.588 (0.433–0.742)	0.588 (0.433–0.742)	0.650 (0.417–0.850)	0.526 (0.278–0.750)
BDI-II & speech model	Random Forests (RF)	20	0.771 (0.629–0.897)	0.825 (0.667–0.954)	0.700 (0.476–0.889)	0.842 (0.667–1.000)
BDI-II & speech model, features corrected for age/education	Linear Model (20)	20	0.642 (0.485–0.795)	0.762 (0.598–0.910)	0.600 (0.385–0.818)	0.684 (0.481–0.882)

BA=Balanced Accuracy; DT=Decision Trees; LM=Linear Model; RF=Random Forests; ROC-AUC=Receiver Operating Characteristic - Area under the Curve; XT=Extra Trees. This table lists the respective best performing models, the full list can be found in Suppl. Table S7

The BDI-II model (ROC-AUC = 0.82 [95% CI: 0.67–0.94]) and the combined model of BDI-II and uncorrected speech features (ROC-AUC = 0.83 [95% CI: 0.67–0.95]) showed the highest predictive performance. Notably, while the BDI-II model alone was highly specific (1.00) but lacked sensitivity (0.55), the addition of speech features in the combined model provided a more balanced diagnostic profile (sensitivity = 0.70; specificity = 0.84).

Although regressing out age and education effects, which altered the feature selection as shown in Supplementary Table S6 (Column 3), led to a performance decrease in the corrected speech-only model (ROC-AUC = 0.59 [95% CI: 0.43–0.74]), the corrected combined model maintained a robust ROC-AUC of 0.76 [95% CI: 0.60–0.91], which is lower than the BDI-II-only model (ROC-AUC = 0.82 [95% CI: 0.67–0.94]). However, the BDI-II-only model demonstrated high specificity (1.000) but low sensitivity (0.550), whereas the corrected combined model yielded a more balanced diagnostic profile with a sensitivity of 0.600 and a specificity of 0.684. This indicates that while the overall discriminative power (ROC-AUC) is not superior to the clinical questionnaire alone, integrating speech markers offers a different clinical trade-off by improving sensitivity at the selected operating point, even after conservative demographic correction.

## Discussion

Our findings demonstrate that a set of distinct speech features can distinguish MDD patients from healthy controls in two independent clinical cohorts. Despite demographic and setting differences between the Aachen and Oldenburg samples, many of the same acoustic markers of depression emerged in both sites, while others demonstrated opposite directions. Notably, a machine learning model trained on Aachen data achieved above-chance performance (AUC = 0.63) when tested on the independent Oldenburg dataset. This cross-site validation suggests that the identified speech markers retain predictive value across different populations, highlighting their potential generalizability as well as their limitations.

Among the most salient speech markers for depressive symptoms replicated in both cohorts were voice quality measures, specifically shimmer (millisecond-by-millisecond fluctuations in vocal loudness that create a sense of hoarseness or crackling) and jitter (rapid fluctuations in the timing or pitch of the voice that are often perceived as a ‘shaky’ or breathy quality) derived from the positive storytelling task. Both were moderately correlated with the BDI-II during the positive narrative, suggesting greater vocal instability among participants reporting more depressive symptoms. At the same time, interaction analyses showed that not all features behaved uniformly across sites: some were consistent, whereas others exhibited opposite trends (e.g., shimmer in negative storytelling; jitter across narratives). Possible explanations include environmental and data-collection differences (recording equipment, background noise) and participant characteristics (cultural background, social factors, symptom severity). Prior work associated increased shimmer and jitter with depression [16, 59–61], our results suggest these findings may not be easily generalizable, which has been noted before across languages [16], and can even exhibit opposite directionalities depending on the site. Physiologically, a biological mechanism, such as reduced laryngeal control, can only account for a unidirectional increase in vocal perturbation, not the site-dependent inversion observed here. Importantly, jitter and shimmer are considered most valid when derived from sustained phonation tasks and are generally less reliable in connected speech, where natural pitch and loudness variation can inflate perturbation estimates [62]. When combined with site-specific recording factors, such as different tablet hardware, room acoustics, and unstandardized mouth-to-microphone distances, these non-biological variables can easily overpower the subtle voice signatures of MDD, leading to the observed technical artifacts and rendering traditional perturbation metrics unreliable for cross-site validation.

In addition, our results demonstrated articulatory and prosodic changes in depressed speech. Across both cohorts, patients with MDD exhibited distinct changes in speech dynamics. Specifically, we observed reduced F2 bandwidth variability in the negative task. While F2 frequency variability reflects tongue body displacement, F2 bandwidth is determined by acoustic damping and losses within the vocal tract. Consequently, a reduction in its bandwidth variability does not index tongue movement directly, but rather points to a rigid, unvarying modulation of vocal tract resonance, likely reflecting sustained tension in the articulatory muscles. This finding aligns in part with recent evidence showing that decreasing standard deviation in F2 bandwidth is a significant marker of depression severity, capturing a loss of dynamic transitions in resonance properties. This effect, however, was language-dependent [16]. Other studies reported a reduced F2 [63] and F2 range [64] in depressed speech, both of which point toward ‘articulatory undershoot’ or vowel centralization. We propose that this restriction in tongue mobility is a direct acoustic manifestation of psychomotor retardation, changing a speaker’s ability to dynamically modulate articulatory precision and vocal tract flexibility [34]. Our finding of more and longer pauses in both conditions are consistent with previous findings [55, 57].

Across both task valences, depressed individuals exhibited significantly higher values for MFCC1 and MFCC3, in line with previous evidence of MFCCs associated with depressive symptomatology [66–69]. MFCCs capture the broad spectral envelope of the voice. Higher values in these coefficients suggest a shift in the distribution of spectral energy, likely reflecting changes in vocal fold adduction and articulation. The elevation of MFCC1 and MFCC3 across both task valences suggests that these markers reflect the physiological state of the person rather than the emotional content of their words used. Specifically, the shift in MFCC1 aligns with a reduction in vocal tension and energy, leading to a breathy voice quality. Meanwhile, the change in MFCC3 points to less precise tongue movements. This matches our other findings (like the correlation of F2 bandwidth variability with BDI-II), providing strong evidence for the physical ‘articulatory undershoot’ or psychomotor retardation common in depression.

The lexical patterns observed in our cohorts offer a complementary window into the cognitive style associated with MDD. Despite having a significantly higher word count in both storytelling tasks, depressed participants showed a significantly lower Type-Token Ratio (TTR) which has been reported before [70]. This combination of high verbal output and low vocabulary diversity may be indicative of a pattern of ‘lexical poverty’ or redundancy. Clinically, this could be interpreted as an objective linguistic marker of rumination; while the patients spoke more at length, they remained trapped within a limited set of words and concepts, suggesting a lack of cognitive flexibility [71] and a repetitive focus during narrative construction.

An interesting aspect of our findings is that certain features, notably shimmer, jitter, and loudness variability, were significantly correlated with BDI-II scores only in the positive storytelling task and not in the negative storytelling. This suggests that the positive narrative may serve as a more effective clinical ‘stress test’; while negative contexts align with the patient’s baseline mood, the demand to express positive affect in the face of anhedonia may more consistently reveal vocal and articulatory deficits. The resulting ‘affective mismatch’ shows subtle instabilities in vocal fold vibration (shimmer/jitter) and a constriction in vocal energy dynamics (reduced loudness variability) that tracks closely with the depth of the depressive state. These markers appear particularly effective at capturing the prosodic ‘dampening’ that occurs when the required emotional modulation exceeds the patient’s current affective range.

However, we also observed significant interaction effects between study cohorts for some of the same voice quality markers. Unlike the temporal features which remained robust, the inconsistent directionality of shimmer and jitter across sites suggests they are highly sensitive to non-biological factors, such as varying recording environments and mouth-to-microphone distances. The significant interaction between diagnosis and site highlights the sensitivity of these amplitude- and frequency-based features to environmental and procedural variances. Site-specific baseline differences, potentially due to microphone distance or room acoustics, may have masked the subtle physiological signatures of MDD. This underscores a current limitation: while task-valence (positive vs. negative) impacts the manifestation of depressive speech, voice quality features may lack the robustness required for cross-site application without strict hardware standardization. Future studies should consider employing more robust measures of vocal stability, such as Cepstral Peak Prominence (CPPS), which has shown greater resilience to varying recording conditions and background noise than traditional perturbation measures [72].

While our study was not designed to directly compare positive vs. negative task performance, the pattern of results suggests that task content may moderate acoustic markers. Prior work has shown that different speech tasks or emotional prompts produce different acoustic profiles in depression [43]. For example, spontaneous autobiographical speech often provokes greater vocal changes than reading aloud, especially if the content is personally relevant [73]. Our results suggest that core features, such as temporal slowing and increased pausing, remain consistent across tasks, while other markers, like shimmer and jitter, demonstrate different patterns depending on the emotional context, likely due to their proneness to cohort-specific influences, such as site-specific recording environments or demographic variability and other contextual or task-related factors that contributed to the observed interaction effects. Thus, using both a positive and a negative storytelling task may provide complementary views of the speech disturbance in MDD.

Analyzing correlations between BDI-II factors and speech domains, the “affective” factor comprising BDI-II items 4, 10, 12, and 13 showed significant correlations with temporal speech features derived from negative and positive storytelling. Features such as utterance duration, pause length, or pause frequency were significantly associated with the combined items of loss of pleasure, crying, loss of interest, and indecisiveness. These findings align with prior research demonstrating the connection between temporal features and MDD [52, 65, 74]. Interestingly, other speech domains did not show significant correlations with the other BDI-II factors (e.g., cognitive or somatic dimensions), suggesting that depression-related speech changes might be more directly tied to affective states than to other symptom domains such as difficulties concentrating, appetite or sleep disturbances or suicidality. The lack of significant correlations between speech domains and other BDI-II factors might also reflect the specificity of the speech features we measured: temporal features may be more sensitive to emotional expression than other speech characteristics, such as voice quality or prosody, which may reflect broader or more generalized deficits in depression, but not necessarily in ways that align with other symptom dimensions.

Our automated machine learning (ML) speech-based models performed significantly better than chance, although they demonstrated modest overall discriminative ability. The unadjusted speech model achieved an AUC of 0.63 (sensitivity = 0.95; specificity = 0.32), which, while lower than the performance observed in our previous single-site study [15] (AUC = 0.93), demonstrates a degree of generalizability across independent clinical sites. A possible explanation of the high sensitivity and the low specificity of the speech model might be that the model overfits on the training distribution which is different to the test distribution with regards to demographic factors and depression severity as reflected in the BDI scores. When accounting for demographic influences by regressing out age and education, the speech-only model’s performance decreased (AUC = 0.59), suggesting that a portion of the acoustic signal in our sample is shared with demographic variance. In the current cross-site validation, the speech model did not outperform clinical classification based on the BDI-II (AUC = 0.82). Notably, the 95% Confidence Intervals for the speech-only, BDI-II, and combined models show substantial overlap, which reflects the statistical uncertainty inherent in our small validation cohort (*n* = 39) and suggests that differences in overall AUC between these modalities should be interpreted with caution.

In this context, we must distinguish between overall discriminative power (ROC-AUC) and the classification trade-offs at specific operating points. The BDI-II alone yields a high overall ROC-AUC (0.82) but suffers from low sensitivity (0.55), failing to detect nearly half of the depressed patients. Integrating speech features stabilizes this threshold: the uncorrected combined model minimally improves classification performance (AUC = 0.83) and offers a more balanced profile (sensitivity = 0.70; specificity = 0.84). Even after conservative demographic correction, where the combined model’s overall ROC-AUC expectedly decreases to 0.76, it still maintains a more balanced operating point (sensitivity = 0.60; specificity = 0.68) than the clinical questionnaire alone. These findings suggest that incorporating speech features may improve a model’s ability to correctly identify cases of MDD while maintaining relatively high accuracy in excluding healthy controls. This balance could be particularly useful in remote monitoring or digital phenotyping, where speech-based assessments can help detect early signs of depression and flag individuals in need of follow-up. It may also support longitudinal monitoring during treatment, providing clinicians with an additional tool to track subtle changes in symptoms over time. Moreover, the model could be valuable in complementing clinical assessments in complex or ambiguous cases, where speech features add another layer of diagnostic information to help guide clinical decisions.

To our knowledge, only a limited number of studies have tested the cross-cohort generalizability of speech-based depression models. Alghowinem et al. [75] demonstrated a substantial drop in classification accuracy when models trained on acoustic speech data from one country were tested on another, highlighting the impact of cultural and paralinguistic variation, such as differences in prosody, emotional expressivity, and speaking style, on model performance, even when linguistic content is not directly analyzed. Similarly, Mitra et al. [76] observed reduced predictive performance when applying models trained on German acoustic speech features to English clinical interviews, suggesting that while some acoustic features may be transferable across languages, generalizability is constrained not only by data mismatch but also by inherent differences in acoustic patterns and sound properties between languages. These linguistic variations affect prosody, phoneme distribution, and speech rhythm, which can impact the reliability of cross-linguistic model performance. Huang et al. [77] further highlighted how models trained on lab-quality speech recordings often degrade in real-world conditions, though they demonstrated that domain adaptation techniques can partially recover performance. To overcome such environmental sensitivities in future clinical implementations, data augmentation strategies could be leveraged during model training. Artificially incorporating background noise or reverberation into the training set may enhance a model’s robustness against the varying recording conditions and hardware-specific artifacts encountered in independent samples. While not implemented in the present study, such techniques, alongside domain adaptation, represent critical steps toward stabilizing performance in ecologically valid settings.

In contrast, some studies have reported better results. Kiss and Vicsi [78] trained regression models on speech in one language (Hungarian) and tested on others (German, Italian), achieving a mean absolute error of approximately 7 points when predicting BDI-II scores. More recently, Guo et al. [79] proposed a mixture-of-experts model that achieved stable depression classification across both Chinese and English datasets by combining speaker-related and emotional speech features in a domain-adaptive framework.

Collectively, these findings reinforce a critical aspect also reflected in our results: models trained and validated within a single dataset tend to perform optimistically, while cross-dataset testing, especially across languages, sites, or acoustic conditions, presents a more stringent and clinically meaningful challenge.

Our study contributes to this body of work by evaluating out-of-sample generalization across two independently collected cohorts, highlighting both the promise and the current limitations of speech-based ML models for depression detection in ecologically valid settings. By leveraging two independent cohorts, our study contributes to the reproducibility of digital speech phenotyping in depression. Lack of replication has been a significant concern in past research as models trained on one dataset often perform poorly on others, due to differences in recording conditions, population, or feature extraction methods. We addressed this by using one cohort purely for validation of findings from the other, effectively performing an external replication. While we observed some consistency in feature effects and a degree of preserved classification performance across sites, the presence of notable interaction effects suggests that certain markers may be variable across cohorts rather than fully generalizable.

There are limitations to our study. The use of internal tablet microphones enhances ecological validity but introduces variability in mouth-to-microphone distance and ambient noise. These factors particularly affect micro-perturbation measures like shimmer and jitter, which are sensitive to signal-to-noise ratios. This technical variability likely produced the observed opposite directionalities in our cohorts, which represent non-biological artifacts of differing recording conditions rather than a true pathophysiological signal. Consequently, because these site-sensitive features carried strong predictive power within the Aachen training cohort, they were selected by our algorithm. Since our feature selection (Suppl. Table S6, column 2) was restricted to the training site to prevent data leakage, the pipeline was blind to their cross-site instability. The demographically corrected models, trained on residuals after regressing out age and education, selected an overlapping but slightly adjusted subset of features (Suppl. Table S6, column 3), which still included these fragile voice quality metrics. The inclusion of these fragile, site-sensitive technical artifacts most likely degraded the model’s cross-site performance, highlighting the need for site-invariant feature selection in future clinical ML applications. Furthermore, lifestyle and clinical factors such as smoking status, BMI, and comorbid anxiety, which have been shown to influence various acoustic properties of the voice [80–82], were not systematically controlled. Additionally, a demographic mismatch between cohorts resulted in some misclassifications within the test set, as the model struggled with participants whose profiles fell outside the training distribution. The samples were also restricted to German-speaking participants, which may not account for regional dialects or socio-cultural nuances in expression. Finally, the small size of the Oldenburg cohort and differences in symptom severity between sites may have limited our power to detect smaller effects. Despite these caveats, our cross-cohort approach remains a notable strength, answering calls for greater standardization and replication in the field [83, 84].

## Conclusions

Our findings indicate that while speech-based markers are promising ‘digital vitals,’ their clinical utility depends on their resilience to environmental variability. Temporal features and spectral markers like MFCCs emerged as the most robust indicators of MDD, making them ideal candidates for longitudinal health tracking - analogous to home blood pressure monitoring. In contrast, the site-dependency of shimmer and jitter highlights a critical need for hardware standardization before voice quality features can be reliably deployed. Furthermore, the low specificity in our validation cohort suggests that future models must be trained on the full clinical spectrum of depression to improve real-world generalizability. Ultimately, these non-invasive tools serve as valuable, scalable complements to standard psychiatric assessments in remote mental health monitoring and relapse prevention.

## Supplementary Information

Below is the link to the electronic supplementary material.

Supplementary Material 1

## Data Availability

The datasets generated and/or analysed during the current study are not publicly available due to the highly sensitive nature of the material, which consists of personal statements from patients with depression and healthy participants.De-identified, numerical feature-level data (extracted acoustic and linguistic markers) and the underlying code for the analysis can be made available to qualified researchers upon reasonable request to the corresponding author (FM).

## Abbreviations

AI: Artificial Intelligence
AUC: Area under the curve
BA: Balanced accuracy
BDAE: Boston Diagnostic Aphasia Examination
BDI-II: Beck Depression Inventory - II
DSM-V: Diagnostic and Statistical Manual of Mental Disorders (5th edition)
DT: Decision Trees
EEG: Electroencephalography
HC: Healthy Controls
HDRS: Hamilton Depression Rating Scale
HNR: Harmonics-to-noise ratio
LM: Linear Model
MADRS: Montgomery‑Åsberg Depression Rating Scale
MFCC: Mel-frequency cepstral coefficient
MDD: Major Depressive Disorder
ML: Machine Learning
NaSSA: Noradrenergic and specific serotonergic antidepressants
RF: Random Forest
ROC: Receiver Operating Characteristic
RWTH: Rheinisch-Westfälische Technische Hochschule
SB-C: Speech Biomarker Cognition
SD: Standard Deviation
SNRI: Serotonin-norepinephrine reuptake inhibitor
SSRI: Selective serotonin reuptake inhibitors
SVM: Support Vector Machine
XT: Extra Trees
