# Supplementary material for: Validating objective and scalable speech markers of depression across two independent psychiatric cohorts
Source: Ann Gen Psychiatry. 2026 Aug 20;25:71. doi: 10.1186/s12991-026-00691-0 (PMC13495444; doi:10.1186/s12991-026-00691-0)
Supplement: Supplementary file 1 [file 12991_2026_691_MOESM1_ESM.docx]

**Supp. Table S1:** Partial Spearman correlations between speech feature domains and BDI-II factors, controlling for cohort, age, and education. *p*=adjusted statistical significance after controlling for multiple comparisons, r=Spearman correlation coefficient.

| **Model** | **Factors** | **Items** | **Energy** | | | | **Frequency** | | | | **Lexical Richness** | | | | **Sentiment** | | | | **Spectral Balance** | | | | **Syntactic Complexity** | | | | **Temporal** | | | | **Word Types** | | | |
| --- | --- | --- | --- | --- | --- | --- | --- | --- | --- | --- | --- | --- | --- | --- | --- | --- | --- | --- | --- | --- | --- | --- | --- | --- | --- | --- | --- | --- | --- | --- | --- | --- | --- | --- |
|  |  |  | **Negative Story** | | **Positive Story** | | **Negative Story** | | **Positive Story** | | **Negative Story** | | **Positive Story** | | **Negative Story** | | **Positive Story** | | **Negative Story** | | **Positive Story** | | **Negative Story** | | **Positive Story** | | **Negative Story** | | **Positive Story** | | **Negative Story** | | **Positive Story** | |
|  |  |  | **r** | ***p*** | **r** | ***p*** | **r** | ***p*** | **r** | ***p*** | **r** | ***p*** | **r** | ***p*** | **r** | ***p*** | **r** | ***p*** | **r** | ***p*** | **r** | ***p*** | **r** | ***p*** | **r** | ***p*** | **r** | ***p*** | **r** | ***p*** | **r** | ***p*** | **r** | ***p*** |
| Model 1¹ | General depression | 1-21 | 0.10 | 0.50 | 0.15 | 0.40 | 0.07 | 0.58 | 0.12 | 0.50 | 0.06 | 0.61 | 0.09 | 0.50 | 0.17 | 0.33 | 0.07 | 0.58 | 0.10 | 0.50 | -0.02 | 0.87 | -0.12 | 0.50 | 0.00 | 0.96 | 0.22 | 0.19 | 0.18 | 0.33 | -0.03 | 0.82 | -0.09 | 0.50 |
| Model 2² | Somatic-affective | 4, 10-13, 15-21 | 0.13 | 0.35 | 0.17 | 0.25 | 0.12 | 0.38 | 0.17 | 0.25 | 0.06 | 0.67 | 0.09 | 0.46 | 0.15 | 0.32 | 0.03 | 0.81 | 0.11 | 0.42 | -0.02 | 0.91 | -0.12 | 0.38 | 0.01 | 0.96 | 0.25 | 0.07 | 0.22 | 0.10 | -0.05 | 0.75 | -0.10 | 0.44 |
|  | Cognitive | 1-3, 5-9, 14 | 0.06 | 0.73 | 0.11 | 0.65 | 0.01 | 0.96 | 0.03 | 0.96 | 0.08 | 0.68 | 0.09 | 0.68 | 0.18 | 0.65 | 0.11 | 0.65 | 0.07 | 0.68 | -0.01 | 0.96 | -0.09 | 0.68 | -0.02 | 0.96 | 0.15 | 0.65 | 0.11 | 0.65 | -0.02 | 0.96 | -0.10 | 0.65 |
| Model 3³ | Cognitive | 1-3, 5-9, 14 | 0.06 | 0.73 | 0.11 | 0.65 | 0.01 | 0.96 | 0.03 | 0.96 | 0.08 | 0.68 | 0.09 | 0.68 | 0.18 | 0.65 | 0.11 | 0.65 | 0.07 | 0.68 | -0.01 | 0.96 | -0.09 | 0.68 | -0.02 | 0.96 | 0.15 | 0.65 | 0.11 | 0.65 | -0.02 | 0.96 | -0.10 | 0.65 |
|  | Affective | 4, 10, 12, 13 | 0.11 | 0.45 | 0.17 | 0.32 | 0.09 | 0.45 | 0.13 | 0.45 | 0.10 | 0.45 | 0.09 | 0.45 | 0.12 | 0.45 | 0.07 | 0.53 | 0.09 | 0.45 | -0.05 | 0.70 | -0.10 | 0.45 | 0.01 | 0.95 | 0.30 | **0.01** | 0.26 | **0.02** | -0.03 | 0.01 | -0.09 | 0.45 |
|  | Somatic | 11, 15-21 | 0.15 | 0.22 | 0.18 | 0.20 | 0.12 | 0.36 | 0.17 | 0.20 | 0.05 | 0.76 | 0.10 | 0.42 | 0.16 | 0.22 | 0.00 | 0.99 | 0.10 | 0.42 | 0.00 | 0.99 | -0.13 | 0.35 | -0.01 | 0.99 | 0.22 | 0.19 | 0.20 | 0.19 | -0.05 | 0.74 | -0.10 | 0.42 |
| Model 4⁴ | Negative Attitude | 1-3, 5-10, 14 | 0.05 | 0.86 | 0.10 | 0.66 | 0.00 | 0.97 | 0.02 | 0.97 | 0.07 | 0.80 | 0.08 | 0.75 | 0.17 | 0.48 | 0.11 | 0.66 | 0.05 | 0.86 | -0.03 | 0.97 | -0.08 | 0.75 | -0.01 | 0.97 | 0.17 | 0.48 | 0.13 | 0.66 | -0.01 | 0.97 | -0.10 | 0.66 |
|  | Performance difficulty | 4, 11-13, 17, 19 | 0.12 | 0.43 | 0.13 | 0.43 | 0.10 | 0.43 | 0.11 | 0.43 | 0.08 | 0.51 | 0.09 | 0.43 | 0.12 | 0.43 | 0.07 | 0.57 | 0.10 | 0.43 | -0.02 | 0.88 | -0.10 | 0.43 | 0.01 | 0.88 | 0.25 | 0.07 | 0.21 | 0.14 | -0.02 | 0.88 | -0.09 | 0.43 |
|  | Somatic elements | 15, 16, 18, 20, 21 | 0.16 | 0.16 | 0.21 | 0.08 | 0.14 | 0.23 | 0.20 | 0.08 | 0.05 | 0.69 | 0.12 | 0.35 | 0.20 | 0.08 | -0.04 | 0.77 | 0.10 | 0.43 | -0.01 | 0.89 | -0.16 | 0.16 | -0.02 | 0.86 | 0.25 | 0.07 | 0.23 | 0.07 | -0.08 | 0.54 | -0.09 | 0.44 |
| Model 5⁵ | General depression | 1-21 | 0.10 | 0.50 | 0.15 | 0.40 | 0.07 | 0.58 | 0.12 | 0.50 | 0.06 | 0.61 | 0.09 | 0.50 | 0.17 | 0.33 | 0.07 | 0.58 | 0.10 | 0.50 | -0.02 | 0.87 | -0.12 | 0.50 | 0.00 | 0.96 | 0.22 | 0.19 | 0.18 | 0.33 | -0.03 | 0.82 | -0.09 | 0.50 |
|  | Cognitive-affective | 1-10, 12, 14 | 0.06 | 0.70 | 0.12 | 0.69 | 0.02 | 0.93 | 0.06 | 0.70 | 0.06 | 0.70 | 0.08 | 0.69 | 0.15 | 0.50 | 0.10 | 0.69 | 0.09 | 0.69 | -0.01 | 0.93 | -0.08 | 0.69 | 0.01 | 0.93 | 0.20 | 0.36 | 0.16 | 0.50 | -0.02 | 0.93 | -0.10 | 0.69 |
|  | Somatic-affective | 11, 13, 17, 19 | 0.12 | 0.59 | 0.10 | 0.59 | 0.09 | 0.59 | 0.08 | 0.59 | 0.08 | 0.59 | 0.09 | 0.59 | 0.11 | 0.59 | 0.04 | 0.78 | 0.04 | 0.78 | -0.05 | 0.78 | -0.07 | 0.61 | 0.00 | 0.97 | 0.23 | 0.14 | 0.20 | 0.19 | -0.01 | 0.97 | -0.12 | 0.59 |
| Model 6⁶ | General depression | 1-21 | 0.10 | 0.50 | 0.15 | 0.40 | 0.07 | 0.58 | 0.12 | 0.50 | 0.06 | 0.61 | 0.09 | 0.50 | 0.17 | 0.33 | 0.07 | 0.58 | 0.10 | 0.50 | -0.02 | 0.87 | -0.12 | 0.50 | 0.00 | 0.96 | 0.22 | 0.19 | 0.18 | 0.33 | -0.03 | 0.82 | -0.09 | 0.50 |
|  | Cognitive | 3, 5-8, 13, 14 | 0.08 | 0.68 | 0.09 | 0.68 | 0.02 | 0.86 | 0.02 | 0.86 | 0.07 | 0.68 | 0.10 | 0.68 | 0.20 | 0.30 | 0.09 | 0.68 | 0.03 | 0.86 | -0.03 | 0.86 | -0.10 | 0.68 | 0.00 | 0.96 | 0.18 | 0.30 | 0.14 | 0.57 | -0.03 | 0.86 | -0.08 | 0.68 |
|  | Affective | 1, 2, 4, 9, 12 | 0.10 | 0.44 | 0.16 | 0.34 | 0.09 | 0.44 | 0.11 | 0.44 | 0.07 | 0.52 | 0.09 | 0.44 | 0.13 | 0.37 | 0.10 | 0.44 | 0.15 | 0.34 | 0.00 | 0.98 | -0.09 | 0.44 | -0.03 | 0.89 | 0.24 | 0.10 | 0.21 | 0.16 | 0.00 | 0.98 | -0.13 | 0.37 |
|  | Somatic | 10, 11, 15-21 | 0.14 | 0.29 | 0.18 | 0.25 | 0.12 | 0.38 | 0.16 | 0.26 | 0.05 | 0.82 | 0.09 | 0.50 | 0.15 | 0.29 | 0.01 | 0.96 | 0.09 | 0.50 | -0.02 | 0.96 | -0.12 | 0.38 | -0.01 | 0.96 | 0.23 | 0.17 | 0.20 | 0.17 | -0.04 | 0.83 | -0.11 | 0.40 |

¹Singular BDI-II items on their own; ²Beck, A. T., Steer, R. A., & Brown, G. (1996). Beck Depression Inventory–II (BDI-II) [Dataset]. <https://doi.org/10.1037/t00742-000>; ³Buckley, T. C., Parker, J. D., & Heggie, J. (2001). A psychometric evaluation of the BDI-II in treatment-seeking substance abusers. Journal of Substance Abuse Treatment, 20(3), 197–204.<https://doi.org/10.1016/S0740-5472(00)00169-0>; ⁴Wu, P.-C., & Huang, T.-W. (2014). Gender-related invariance of the Beck Depression Inventory II for Taiwanese adolescent samples. Assessment, 21(2), 218–226. <https://doi.org/10.1177/1073191112441243>; ⁵Faro, A., & Pereira, C. R. (2020). Factor structure and gender invariance of the Beck Depression Inventory – second edition (BDI-II) in a community-dwelling sample of adults. Health Psychology and Behavioral Medicine, 8(1), 16–31. <https://doi.org/10.1080/21642850.2020.1715222>; ⁶García-Batista, Z. E., Guerra-Peña, K., Cano-Vindel, A., Herrera-Martínez, S. X., & Medrano, L. A. (2018). Validity and reliability of the Beck Depression Inventory (BDI-II) in general and hospital population of Dominican Republic. PLOS ONE, 13(6), e0199750. <https://doi.org/10.1371/journal.pone.0199750>

**Supp. Table S2**: Group differences as assessed by linear models controlling for age, education, sex, and sample effects between healthy controls and depressed patients for both cohorts. Variables are listed in descending order for adjusted p value. pos=positive story; neg=negative story. T=t-value of predictor in linear model; β=normalized beta coefficient; Adj. *p*=*p* value adjusted for multiple hypothesis testing according to the Benjamini-Hochberg procedure.

| **Speech feature** | **HC mean** | **HC SD** | **MDD mean** | **MDD SD** | ***t*-Value** | ***p*** | **β** | **adj. *p*** |
| --- | --- | --- | --- | --- | --- | --- | --- | --- |
| **average_mfccs_1_pos** | 90.507 | 13.698 | 101.896 | 12.369 | 4.628 | 0 | 0.367 | 0.001 |
| **type_token_ratio_neg** | 0.754 | 0.129 | 0.65 | 0.177 | -4.357 | 0 | -0.382 | 0.002 |
| **duration_neg** | 30.85 | 19.955 | 80.618 | 104.188 | 4.174 | 0 | 0.374 | 0.002 |
| **utterance_durations_sum_neg** | 11.837 | 8.204 | 30.884 | 37.509 | 4.194 | 0 | 0.373 | 0.002 |
| **utterance_durations_sum_pos** | 10.203 | 11.137 | 20.788 | 21.591 | 3.924 | 0 | 0.33 | 0.003 |
| **duration_pos** | 26.06 | 22.528 | 53.538 | 59.593 | 4.012 | 0 | 0.347 | 0.003 |
| **number_of_pauses_neg** | 79.676 | 52.335 | 187.969 | 241.663 | 3.929 | 0 | 0.355 | 0.003 |
| **word_count_neg** | 72.211 | 51.774 | 175.297 | 231.757 | 4.012 | 0 | 0.361 | 0.003 |
| **number_of_pauses_pos** | 67.676 | 54.912 | 124.828 | 129.681 | 3.883 | 0 | 0.339 | 0.003 |
| **pause_durations_sum_neg** | 17.166 | 12.951 | 46.958 | 67.061 | 3.995 | 0 | 0.361 | 0.003 |
| **pause_durations_sum_pos** | 14.363 | 12.544 | 30.742 | 39.002 | 3.773 | 0 | 0.333 | 0.004 |
| **word_count_pos** | 63.817 | 60.588 | 116.781 | 135.506 | 3.566 | 0.001 | 0.313 | 0.006 |
| **average_mfccs_1_neg** | 90.723 | 13.921 | 99.479 | 11.582 | 3.597 | 0 | 0.292 | 0.006 |
| **average_mfccs_3_neg** | 18.33 | 6.707 | 23.58 | 7.896 | 3.524 | 0.001 | 0.271 | 0.007 |
| **average_mfccs_3_pos** | 17.498 | 7.27 | 22.085 | 6.558 | 3.331 | 0.001 | 0.255 | 0.011 |
| **type_token_ratio_pos** | 0.774 | 0.132 | 0.703 | 0.16 | -3.35 | 0.001 | -0.292 | 0.011 |
| **f2_relative_energy_sd_pos** | 9.598 | 1.228 | 8.847 | 1.059 | -3.08 | 0.003 | -0.261 | 0.024 |
| **utterance_durations_mean_pos** | 0.141 | 0.04 | 0.17 | 0.049 | 2.971 | 0.004 | 0.212 | 0.032 |
| **average_mfccs_4_neg** | 3.467 | 6.619 | 8.00 | 9.071 | 2.87 | 0.005 | 0.201 | 0.039 |
| **utterance_durations_mean_neg** | 0.145 | 0.041 | 0.169 | 0.045 | 2.871 | 0.005 | 0.199 | 0.039 |
| **hnr_sd_pos** | 6.2 | 0.897 | 5.764 | 0.767 | -2.764 | 0.007 | -0.226 | 0.051 |
| **dda_shimmer_pos** | 17.863 | 3.94 | 20.501 | 5.538 | 2.662 | 0.009 | 0.233 | 0.059 |
| **apq3_shimmer_pos** | 5.954 | 1.313 | 6.834 | 1.846 | 2.662 | 0.009 | 0.233 | 0.059 |
| **number_consecutive_repetitions_neg** | 0.113 | 0.433 | 0.453 | 1.112 | 2.667 | 0.009 | 0.244 | 0.059 |
| **honore_stat_neg** | 2783.805 | 1336.795 | 2223.999 | 898.921 | -2.644 | 0.009 | -0.238 | 0.06 |
| **mean_number_subordinate_clauses_pos** | 1.199 | 1.534 | 1.781 | 1.908 | 2.474 | 0.015 | 0.228 | 0.092 |
| **loudness_mean_pos** | -37.491 | 4.2 | -36.105 | 4.844 | 2.417 | 0.017 | 0.22 | 0.103 |
| **perceived_loudness_mean_pos** | -38.048 | 4.187 | -36.784 | 4.93 | 2.277 | 0.024 | 0.208 | 0.136 |
| **apq5_shimmer_pos** | 8.229 | 1.967 | 9.399 | 2.662 | 2.256 | 0.026 | 0.188 | 0.136 |
| **pitch_std_pos** | 36.393 | 16.011 | 29.372 | 13.114 | -2.273 | 0.025 | -0.184 | 0.136 |
| **noun_rate_pos** | 0.137 | 0.054 | 0.116 | 0.056 | -2.265 | 0.025 | -0.212 | 0.136 |
| **conjunction_rate_pos** | 0.064 | 0.037 | 0.069 | 0.036 | 2.168 | 0.032 | 0.196 | 0.149 |
| **f2_bandwidth_sd_neg** | 365.679 | 54.552 | 342.872 | 57.958 | -2.205 | 0.029 | -0.207 | 0.149 |
| **spectral_slope_0_500_sd_neg** | 2.376 | 0.329 | 2.288 | 0.295 | -2.192 | 0.03 | -0.179 | 0.149 |
| **number_consecutive_repetitions_pos** | 0.169 | 0.447 | 0.344 | 0.718 | 2.171 | 0.032 | 0.203 | 0.149 |
| **h1_a3_harmonic_difference_mean_pos** | -19.304 | 3.573 | -21.526 | 3.782 | -2.127 | 0.035 | -0.188 | 0.16 |
| **loudness_mean_neg** | -38.036 | 4.127 | -36.683 | 4.732 | 2.085 | 0.039 | 0.19 | 0.163 |
| **h1_h2_harmonic_difference_sd_neg** | 8.035 | 1.215 | 7.354 | 1.351 | -2.094 | 0.038 | -0.178 | 0.163 |
| **determiner_rate_pos** | 0.096 | 0.046 | 0.08 | 0.031 | -2.093 | 0.038 | -0.192 | 0.163 |
| **pronoun_rate_neg** | 0.122 | 0.044 | 0.136 | 0.045 | 2.067 | 0.041 | 0.194 | 0.166 |
| **average_mfccs_4_pos** | 3.029 | 6.379 | 6.61 | 9.437 | 2.038 | 0.044 | 0.145 | 0.173 |
| **verb_rate_neg** | 0.112 | 0.035 | 0.102 | 0.027 | -1.978 | 0.05 | -0.181 | 0.176 |
| **apq5_shimmer_neg** | 8.262 | 2.281 | 9.307 | 2.228 | 1.998 | 0.048 | 0.167 | 0.176 |
| **perceived_loudness_mean_neg** | -38.62 | 4.126 | -37.368 | 4.828 | 1.975 | 0.05 | 0.181 | 0.176 |
| **local_dB_shimmer_pos** | 129.229 | 17.989 | 136.742 | 18.715 | 2.019 | 0.046 | 0.166 | 0.176 |
| **hammarberg_index_mean_pos** | 24.092 | 3.24 | 25.618 | 3.324 | 2.005 | 0.047 | 0.179 | 0.176 |
| **f3_frequency_mean_pos** | 2744.917 | 127.375 | 2720.329 | 127.528 | -1.971 | 0.051 | -0.176 | 0.176 |
| **local_shimmer_pos** | 13.873 | 2.417 | 14.89 | 2.811 | 1.956 | 0.053 | 0.163 | 0.179 |
| **f2_bandwidth_sd_pos** | 373.646 | 58.867 | 354.615 | 56.01 | -1.933 | 0.055 | -0.183 | 0.184 |
| **adverb_rate_pos** | 0.158 | 0.071 | 0.177 | 0.071 | 1.849 | 0.067 | 0.173 | 0.202 |
| **h1_a3_harmonic_difference_mean_neg** | -19.556 | 3.499 | -21.457 | 3.761 | -1.875 | 0.063 | -0.169 | 0.202 |
| **ddp_jitter_pos** | 3.523 | 1.008 | 3.904 | 1.028 | 1.862 | 0.065 | 0.151 | 0.202 |
| **apq11_shimmer_neg** | 13.494 | 4.601 | 15.062 | 3.387 | 1.848 | 0.067 | 0.158 | 0.202 |
| **rap_jitter_pos** | 1.174 | 0.336 | 1.301 | 0.343 | 1.862 | 0.065 | 0.151 | 0.202 |
| **speech_ratio_pos** | 0.367 | 0.097 | 0.414 | 0.108 | 1.807 | 0.073 | 0.158 | 0.212 |
| **ddp_jitter_neg** | 3.505 | 1.159 | 3.967 | 1.039 | 1.801 | 0.074 | 0.149 | 0.212 |
| **rap_jitter_neg** | 1.168 | 0.386 | 1.322 | 0.346 | 1.801 | 0.074 | 0.149 | 0.212 |
| **mean_number_subordinate_clauses_neg** | 1.68 | 1.794 | 1.102 | 1.345 | -1.684 | 0.095 | -0.156 | 0.253 |
| **hammarberg_index_mean_neg** | 24.152 | 2.996 | 25.341 | 2.946 | 1.686 | 0.094 | 0.154 | 0.253 |
| **f2_bandwidth_mean_pos** | 515.368 | 96.399 | 499.141 | 98.244 | -1.701 | 0.091 | -0.157 | 0.253 |
| **proportion_verb_phrase_with_subjects_pos** | 0.788 | 0.199 | 0.811 | 0.179 | 1.7 | 0.091 | 0.156 | 0.253 |
| **utterance_durations_sd_pos** | 0.13 | 0.07 | 0.159 | 0.07 | 1.642 | 0.103 | 0.125 | 0.271 |
| **h1_h2_harmonic_difference_sd_pos** | 8.211 | 1.543 | 7.427 | 1.44 | -1.618 | 0.108 | -0.131 | 0.28 |
| **loudness_sd_pos** | 4.92 | 0.736 | 4.725 | 0.689 | -1.604 | 0.111 | -0.148 | 0.281 |
| **adjective_rate_pos** | 0.067 | 0.045 | 0.055 | 0.037 | -1.6 | 0.112 | -0.152 | 0.281 |
| **f1_frequency_mean_pos** | 587.575 | 60.336 | 571.837 | 55.019 | -1.575 | 0.118 | -0.14 | 0.291 |
| **ppq5_jitter_neg** | 1.314 | 0.437 | 1.452 | 0.336 | 1.558 | 0.122 | 0.122 | 0.296 |
| **hnr_sd_neg** | 6.064 | 0.791 | 5.802 | 0.978 | -1.536 | 0.127 | -0.131 | 0.304 |
| **local_jitter_neg** | 2.268 | 0.676 | 2.502 | 0.58 | 1.522 | 0.13 | 0.126 | 0.308 |
| **local_jitter_pos** | 2.289 | 0.585 | 2.497 | 0.68 | 1.437 | 0.153 | 0.116 | 0.343 |
| **f3_frequency_sd_neg** | 286.609 | 59.535 | 312.577 | 74.426 | 1.431 | 0.155 | 0.114 | 0.343 |
| **ppq5_jitter_pos** | 1.337 | 0.372 | 1.444 | 0.344 | 1.427 | 0.156 | 0.111 | 0.343 |
| **f3_relative_energy_sd_pos** | 9.239 | 1.293 | 8.834 | 1.245 | -1.433 | 0.154 | -0.131 | 0.343 |
| **dda_shimmer_neg** | 18.205 | 4.549 | 20.024 | 4.821 | 1.42 | 0.158 | 0.126 | 0.343 |
| **apq3_shimmer_neg** | 6.068 | 1.516 | 6.675 | 1.607 | 1.42 | 0.158 | 0.126 | 0.343 |
| **h1_a3_harmonic_difference_sd_pos** | 8.647 | 1.23 | 8.424 | 1.17 | -1.41 | 0.161 | -0.134 | 0.345 |
| **f3_relative_energy_mean_neg** | -21.353 | 5.117 | -23.175 | 4.388 | -1.398 | 0.165 | -0.116 | 0.348 |
| **pitch_mean_pos** | 166.546 | 44.39 | 153.141 | 38.552 | -1.386 | 0.168 | -0.064 | 0.351 |
| **utterance_durations_sd_neg** | 0.137 | 0.059 | 0.16 | 0.066 | 1.368 | 0.174 | 0.094 | 0.358 |
| **alpha_ratio_mean_neg** | 7.373 | 2.432 | 8.271 | 2.507 | 1.352 | 0.179 | 0.125 | 0.364 |
| **f1_relative_energy_sd_pos** | 7.415 | 1.172 | 7.065 | 1.046 | -1.322 | 0.189 | -0.11 | 0.378 |
| **f1_bandwidth_sd_pos** | 237.072 | 47.983 | 234.551 | 48.167 | -1.317 | 0.19 | -0.116 | 0.378 |
| **f3_frequency_sd_pos** | 286.369 | 66.35 | 311.957 | 63.728 | 1.305 | 0.194 | 0.102 | 0.381 |
| **local_dB_shimmer_neg** | 129.195 | 21.044 | 135.577 | 18.882 | 1.295 | 0.197 | 0.109 | 0.383 |
| **perceived_loudness_sd_pos** | 4.638 | 0.618 | 4.486 | 0.568 | -1.28 | 0.203 | -0.118 | 0.389 |
| **f3_relative_energy_mean_pos** | -21.511 | 5.618 | -23.303 | 4.728 | -1.234 | 0.219 | -0.104 | 0.411 |
| **rate_loudness_peaks_neg** | 6.483 | 0.99 | 6.031 | 1.05 | -1.235 | 0.219 | -0.106 | 0.411 |
| **hammarberg_index_sd_pos** | 7.895 | 1.624 | 7.852 | 1.479 | -1.14 | 0.257 | -0.105 | 0.455 |
| **f2_bandwidth_mean_neg** | 500.867 | 88.141 | 492.032 | 101.786 | -1.149 | 0.253 | -0.108 | 0.455 |
| **spectral_slope_0_500_sd_pos** | 2.383 | 0.363 | 2.347 | 0.352 | -1.142 | 0.255 | -0.091 | 0.455 |
| **local_shimmer_neg** | 13.824 | 2.941 | 14.669 | 2.652 | 1.158 | 0.249 | 0.097 | 0.455 |
| **noun_rate_neg** | 0.12 | 0.039 | 0.11 | 0.05 | -1.159 | 0.248 | -0.109 | 0.455 |
| **f1_bandwidth_mean_pos** | 309.748 | 74.999 | 313.804 | 76.644 | -1.114 | 0.267 | -0.095 | 0.468 |
| **alpha_ratio_sd_neg** | 6.064 | 2.285 | 6.681 | 2.324 | 1.109 | 0.27 | 0.104 | 0.468 |
| **f2_relative_energy_mean_neg** | -13.665 | 5.103 | -15.436 | 4.524 | -1.097 | 0.275 | -0.091 | 0.471 |
| **rate_loudness_peaks_pos** | 6.574 | 1.162 | 6.055 | 1.249 | -1.059 | 0.292 | -0.091 | 0.49 |
| **pause_rate_pos** | 0.541 | 0.105 | 0.505 | 0.141 | -1.059 | 0.292 | -0.1 | 0.49 |
| **proper_noun_rate_neg** | 0.01 | 0.031 | 0.005 | 0.014 | -1.038 | 0.301 | -0.098 | 0.496 |
| **proportion_verb_phrase_with_objects_pos** | 0.324 | 0.172 | 0.288 | 0.113 | -1.041 | 0.3 | -0.098 | 0.496 |
| **local_absolute_jitter_pos** | 0.015 | 0.007 | 0.017 | 0.007 | 1.018 | 0.31 | 0.065 | 0.501 |
| **f3_frequency_mean_neg** | 2742.632 | 132.946 | 2737.932 | 122.939 | -1.023 | 0.308 | -0.091 | 0.501 |
| **local_absolute_jitter_neg** | 0.016 | 0.008 | 0.018 | 0.007 | 0.983 | 0.328 | 0.061 | 0.518 |
| **f1_relative_energy_sd_neg** | 7.445 | 1.162 | 7.107 | 1.078 | -0.986 | 0.326 | -0.087 | 0.518 |
| **f3_bandwidth_sd_pos** | 359.951 | 60.678 | 365.763 | 77.731 | 0.96 | 0.339 | 0.087 | 0.526 |
| **spectral_slope_500_1500_sd_neg** | 5.489 | 0.717 | 5.334 | 0.932 | -0.961 | 0.338 | -0.09 | 0.526 |
| **f2_frequency_mean_pos** | 1635.729 | 95.075 | 1626.647 | 102.574 | -0.934 | 0.352 | -0.088 | 0.542 |
| **f2_relative_energy_sd_neg** | 9.409 | 1.432 | 8.871 | 1.463 | -0.89 | 0.375 | -0.076 | 0.566 |
| **f1_frequency_mean_neg** | 580.424 | 53.331 | 570.329 | 63.933 | -0.891 | 0.375 | -0.082 | 0.566 |
| **pronoun_rate_pos** | 0.124 | 0.054 | 0.129 | 0.044 | 0.872 | 0.385 | 0.083 | 0.575 |
| **h1_h2_harmonic_difference_mean_pos** | 4.224 | 3.699 | 5.295 | 3.143 | 0.861 | 0.391 | 0.08 | 0.579 |
| **h1_h2_harmonic_difference_mean_neg** | 4.271 | 3.461 | 5.478 | 3.108 | 0.846 | 0.399 | 0.077 | 0.586 |
| **proportion_verb_phrase_with_subjects_neg** | 0.805 | 0.165 | 0.816 | 0.19 | 0.823 | 0.412 | 0.077 | 0.6 |
| **verb_phrase_with_aux_and_vp_rate_pos** | 0.046 | 0.143 | 0.035 | 0.077 | -0.809 | 0.42 | -0.075 | 0.606 |
| **honore_stat_pos** | 2709.491 | 1734.178 | 2616.053 | 1595.807 | -0.802 | 0.424 | -0.075 | 0.607 |
| **pitch_range_neg** | 248.254 | 118.613 | 245.519 | 144.299 | 0.765 | 0.446 | 0.07 | 0.632 |
| **f1_relative_energy_mean_neg** | 0.594 | 4.177 | -0.801 | 3.94 | -0.756 | 0.451 | -0.065 | 0.634 |
| **pitch_std_neg** | 31.075 | 11.098 | 27.878 | 11.634 | -0.741 | 0.46 | -0.057 | 0.641 |
| **proper_noun_rate_pos** | 0.013 | 0.025 | 0.012 | 0.021 | -0.707 | 0.481 | -0.065 | 0.65 |
| **f1_frequency_sd_neg** | 223.801 | 68.737 | 247.444 | 84.051 | 0.707 | 0.481 | 0.061 | 0.65 |
| **alpha_ratio_sd_pos** | 6.532 | 2.625 | 6.551 | 2.346 | -0.71 | 0.479 | -0.066 | 0.65 |
| **hnr_mean_pos** | 10.833 | 2.382 | 10.322 | 2.177 | -0.704 | 0.483 | -0.056 | 0.65 |
| **verb_phrase_with_aux_rate_neg** | 0.507 | 0.325 | 0.492 | 0.21 | -0.688 | 0.493 | -0.063 | 0.658 |
| **alpha_ratio_mean_pos** | 7.539 | 2.53 | 8.227 | 2.835 | 0.654 | 0.514 | 0.06 | 0.681 |
| **adposition_rate_neg** | 0.058 | 0.036 | 0.059 | 0.033 | 0.645 | 0.52 | 0.061 | 0.684 |
| **f2_relative_energy_mean_pos** | -13.868 | 5.314 | -15.106 | 4.76 | -0.636 | 0.526 | -0.054 | 0.685 |
| **inflected_verb_rate_pos** | 0.702 | 0.263 | 0.698 | 0.281 | 0.631 | 0.529 | 0.059 | 0.685 |
| **proportion_verb_phrase_with_objects_neg** | 0.282 | 0.182 | 0.271 | 0.109 | -0.593 | 0.554 | -0.057 | 0.711 |
| **verb_rate_pos** | 0.099 | 0.038 | 0.101 | 0.041 | 0.568 | 0.571 | 0.054 | 0.724 |
| **pause_rate_neg** | 0.536 | 0.104 | 0.522 | 0.125 | 0.565 | 0.573 | 0.051 | 0.724 |
| **adverb_rate_neg** | 0.176 | 0.072 | 0.185 | 0.064 | 0.553 | 0.581 | 0.052 | 0.728 |
| **determiner_rate_neg** | 0.086 | 0.035 | 0.08 | 0.033 | -0.513 | 0.609 | -0.049 | 0.757 |
| **conjunction_rate_neg** | 0.078 | 0.036 | 0.078 | 0.029 | 0.46 | 0.646 | 0.042 | 0.79 |
| **speech_ratio_neg** | 0.378 | 0.093 | 0.403 | 0.093 | 0.456 | 0.649 | 0.039 | 0.79 |
| **f3_bandwidth_mean_pos** | 568.863 | 108.291 | 586.094 | 98.649 | 0.458 | 0.648 | 0.042 | 0.79 |
| **f2_frequency_sd_neg** | 347.368 | 51.9 | 359.66 | 78.494 | 0.413 | 0.68 | 0.038 | 0.809 |
| **f2_frequency_sd_pos** | 343.669 | 49.919 | 361.312 | 77.016 | 0.419 | 0.676 | 0.037 | 0.809 |
| **f1_frequency_sd_pos** | 226.57 | 65.27 | 246.984 | 83.264 | 0.417 | 0.677 | 0.036 | 0.809 |
| **spectral_slope_500_1500_mean_neg** | -10.555 | 1.925 | -10.12 | 2.381 | 0.406 | 0.685 | 0.036 | 0.81 |
| **pitch_range_pos** | 274.444 | 135.974 | 248.872 | 142.793 | -0.396 | 0.693 | -0.036 | 0.813 |
| **spectral_slope_500_1500_sd_pos** | 5.461 | 0.752 | 5.408 | 0.917 | -0.384 | 0.702 | -0.036 | 0.817 |
| **f1_relative_energy_mean_pos** | 0.48 | 4.394 | -0.486 | 4.128 | -0.366 | 0.715 | -0.033 | 0.827 |
| **f3_relative_energy_sd_neg** | 8.93 | 1.361 | 8.885 | 1.574 | 0.349 | 0.728 | 0.032 | 0.835 |
| **verb_phrase_with_aux_and_vp_rate_neg** | 0.029 | 0.076 | 0.027 | 0.05 | 0.335 | 0.738 | 0.031 | 0.841 |
| **pitch_mean_neg** | 157.645 | 39.367 | 149.447 | 37.494 | -0.313 | 0.755 | -0.014 | 0.849 |
| **spectral_slope_0_500_mean_pos** | 2.736 | 1.826 | 2.111 | 2.228 | -0.316 | 0.753 | -0.027 | 0.849 |
| **inflected_verb_rate_neg** | 0.78 | 0.221 | 0.767 | 0.201 | -0.284 | 0.777 | -0.027 | 0.867 |
| **average_mfccs_2_pos** | 8.86 | 10.6 | 10.809 | 10.904 | -0.241 | 0.81 | -0.022 | 0.893 |
| **f3_bandwidth_mean_neg** | 560.243 | 97.585 | 571.445 | 99.674 | 0.24 | 0.811 | 0.022 | 0.893 |
| **f1_bandwidth_sd_neg** | 229.784 | 51.762 | 237.568 | 47.041 | -0.181 | 0.857 | -0.016 | 0.937 |
| **spectral_slope_0_500_mean_neg** | 2.502 | 1.657 | 1.938 | 2.148 | -0.166 | 0.868 | -0.014 | 0.942 |
| **adposition_rate_pos** | 0.066 | 0.037 | 0.068 | 0.039 | 0.161 | 0.872 | 0.015 | 0.942 |
| **hammarberg_index_sd_neg** | 7.723 | 1.437 | 7.907 | 1.55 | -0.151 | 0.88 | -0.014 | 0.944 |
| **f3_bandwidth_sd_neg** | 357.2 | 56.994 | 357.482 | 64.688 | 0.134 | 0.894 | 0.012 | 0.952 |
| **spectral_slope_500_1500_mean_pos** | -10.21 | 2.077 | -9.969 | 2.427 | 0.083 | 0.934 | 0.008 | 0.97 |
| **adjective_rate_neg** | 0.047 | 0.034 | 0.044 | 0.023 | -0.089 | 0.929 | -0.008 | 0.97 |
| **f1_bandwidth_mean_neg** | 298.375 | 65.407 | 314.91 | 75.78 | 0.084 | 0.933 | 0.007 | 0.97 |
| **f2_frequency_mean_neg** | 1635.068 | 106.459 | 1651.189 | 108.681 | 0.075 | 0.94 | 0.007 | 0.97 |
| **h1_a3_harmonic_difference_sd_neg** | 8.36 | 1.171 | 8.439 | 1.384 | -0.093 | 0.926 | -0.009 | 0.97 |
| **perceived_loudness_sd_neg** | 4.527 | 0.533 | 4.468 | 0.596 | -0.067 | 0.947 | -0.006 | 0.971 |
| **verb_phrase_with_aux_rate_pos** | 0.512 | 0.336 | 0.534 | 0.335 | -0.04 | 0.968 | -0.004 | 0.98 |
| **loudness_sd_neg** | 4.768 | 0.597 | 4.734 | 0.707 | -0.045 | 0.964 | -0.004 | 0.98 |
| **hnr_mean_neg** | 10.621 | 2.483 | 10.276 | 2.383 | 0.028 | 0.978 | 0.002 | 0.984 |
| **average_mfccs_2_neg** | 11.119 | 10.575 | 12.746 | 10.479 | 0.001 | 1 | 0 | 1 |

**Supp. Table S3:** Results of moderation analysis assessing the interaction between diagnosis (MDD vs. HC) and location (Aachen vs. Oldenburg) in relation to speech features. The model was built with the formula: feature ~ diagnosis + location + age + education + sex + diagnosis*location, with age, sex, and education included as covariates to control for their effects. The table reports the mean ± SD for each diagnosis and location group, along with the t-value and corresponding p-value for the interaction term. The change in R-squared (ΔR²) between the interaction model and a model without the interaction term is also provided to estimate the effect size of the interaction.

|  | **Mean HC Aachen** | **SD HC Aachen** | **Mean MDD Aachen** | **SD MDD Aachen** | **Mean HC Oldenburg** | **SD HC Oldenburg** | **Mean MDD Oldenburg** | **SD MDD Oldenburg** | ***t*-Value** | **ΔR²** | **adj. *p*** |
| --- | --- | --- | --- | --- | --- | --- | --- | --- | --- | --- | --- |
| **local_dB_shimmer_neg** | 121.34 | 17.705 | 135.223 | 18.814 | 150.691 | 12.964 | 136.358 | 19.5 | -4.326 | 0.098 | 0.001 |
| **apq5_shimmer_neg** | 7.368 | 1.805 | 9.286 | 2.355 | 10.709 | 1.536 | 9.352 | 1.975 | -4.401 | 0.099 | 0.001 |
| **apq3_shimmer_neg** | 5.504 | 1.228 | 6.766 | 1.71 | 7.611 | 1.113 | 6.475 | 1.371 | -4.588 | 0.121 | 0.001 |
| **local_shimmer_neg** | 12.704 | 2.397 | 14.657 | 2.691 | 16.89 | 1.949 | 14.693 | 2.633 | -4.585 | 0.107 | 0.001 |
| **average_mfccs_1_pos** | 85.245 | 10.606 | 101.797 | 13.635 | 104.906 | 10.59 | 102.115 | 9.298 | -4.316 | 0.086 | 0.001 |
| **dda_shimmer_neg** | 16.513 | 3.684 | 20.297 | 5.131 | 22.834 | 3.338 | 19.424 | 4.112 | -4.588 | 0.121 | 0.001 |
| **hnr_sd_neg** | 6.333 | 0.704 | 5.741 | 0.898 | 5.33 | 0.507 | 5.934 | 1.148 | 4.031 | 0.088 | 0.002 |
| **apq11_shimmer_neg** | 12.001 | 4.03 | 14.969 | 3.645 | 17.58 | 3.501 | 15.266 | 2.813 | -3.716 | 0.077 | 0.005 |
| **hnr_sd_pos** | 6.451 | 0.772 | 5.741 | 0.72 | 5.513 | 0.872 | 5.814 | 0.879 | 3.7 | 0.07 | 0.005 |
| **average_mfccs_1_neg** | 86.286 | 12.044 | 99.388 | 13.039 | 102.869 | 11.403 | 99.682 | 7.743 | -3.743 | 0.07 | 0.005 |
| **ppq5_jitter_neg** | 1.156 | 0.321 | 1.404 | 0.306 | 1.745 | 0.428 | 1.56 | 0.379 | -3.347 | 0.054 | 0.016 |
| **ddp_jitter_neg** | 3.109 | 0.891 | 3.871 | 1.018 | 4.591 | 1.129 | 4.177 | 1.079 | -3.092 | 0.052 | 0.031 |
| **rap_jitter_neg** | 1.036 | 0.297 | 1.29 | 0.339 | 1.53 | 0.376 | 1.392 | 0.36 | -3.092 | 0.052 | 0.031 |
| **local_dB_shimmer_pos** | 122.215 | 14.258 | 134.412 | 19.876 | 148.427 | 12.297 | 141.869 | 15.068 | -3.054 | 0.05 | 0.032 |
| **ppq5_jitter_pos** | 1.182 | 0.238 | 1.362 | 0.349 | 1.761 | 0.345 | 1.625 | 0.26 | -2.726 | 0.036 | 0.052 |
| **ddp_jitter_pos** | 3.109 | 0.66 | 3.706 | 1.08 | 4.656 | 0.929 | 4.34 | 0.76 | -2.719 | 0.039 | 0.052 |
| **loudness_sd_neg** | 4.919 | 0.583 | 4.71 | 0.655 | 4.354 | 0.421 | 4.786 | 0.826 | 2.708 | 0.052 | 0.052 |
| **utterance_durations_sd_pos** | 0.099 | 0.026 | 0.143 | 0.059 | 0.213 | 0.084 | 0.196 | 0.08 | -2.785 | 0.036 | 0.052 |
| **speech_ratio_pos** | 0.335 | 0.077 | 0.406 | 0.113 | 0.455 | 0.095 | 0.433 | 0.096 | -2.808 | 0.048 | 0.052 |
| **hnr_mean_neg** | 11.325 | 2.378 | 10.394 | 2.34 | 8.693 | 1.611 | 10.017 | 2.516 | 2.738 | 0.041 | 0.052 |
| **loudness_sd_pos** | 5.078 | 0.774 | 4.695 | 0.703 | 4.489 | 0.379 | 4.79 | 0.668 | 2.763 | 0.052 | 0.052 |
| **rap_jitter_pos** | 1.036 | 0.22 | 1.235 | 0.36 | 1.552 | 0.31 | 1.447 | 0.253 | -2.719 | 0.039 | 0.052 |
| **local_jitter_neg** | 2.052 | 0.541 | 2.442 | 0.572 | 2.857 | 0.668 | 2.634 | 0.59 | -2.734 | 0.041 | 0.052 |
| **average_mfccs_4_pos** | 4.459 | 5.473 | 10.43 | 7.741 | -0.885 | 7.162 | -1.795 | 7.177 | -2.836 | 0.033 | 0.052 |
| **perceived_loudness_sd_pos** | 4.77 | 0.644 | 4.469 | 0.567 | 4.278 | 0.348 | 4.524 | 0.583 | 2.683 | 0.049 | 0.054 |
| **dda_shimmer_pos** | 16.424 | 3.021 | 20.227 | 5.917 | 21.803 | 3.487 | 21.104 | 4.677 | -2.625 | 0.042 | 0.059 |
| **apq3_shimmer_pos** | 5.475 | 1.007 | 6.742 | 1.972 | 7.268 | 1.162 | 7.035 | 1.559 | -2.625 | 0.042 | 0.059 |
| **local_shimmer_pos** | 12.972 | 1.912 | 14.568 | 2.925 | 16.338 | 1.898 | 15.6 | 2.464 | -2.547 | 0.036 | 0.07 |
| **perceived_loudness_sd_neg** | 4.658 | 0.52 | 4.467 | 0.539 | 4.169 | 0.394 | 4.471 | 0.72 | 2.416 | 0.041 | 0.096 |
| **hnr_mean_pos** | 11.445 | 2.299 | 10.471 | 2.198 | 9.159 | 1.747 | 9.995 | 2.151 | 2.33 | 0.028 | 0.116 |
| **spectral_slope_500_1500_mean_neg** | -10.694 | 2.036 | -9.741 | 2.247 | -10.174 | 1.568 | -10.954 | 2.513 | -2.299 | 0.034 | 0.122 |
| **f2_relative_energy_sd_pos** | 9.903 | 1.213 | 8.94 | 1.153 | 8.764 | 0.833 | 8.645 | 0.804 | 2.218 | 0.029 | 0.144 |
| **average_mfccs_4_neg** | 5.13 | 5.126 | 11.66 | 7.448 | -1.085 | 8.127 | -0.052 | 6.933 | -2.184 | 0.019 | 0.152 |
| **f2_bandwidth_mean_neg** | 504.114 | 91.502 | 472.885 | 85.799 | 491.981 | 79.85 | 534.156 | 122.375 | 2.088 | 0.032 | 0.186 |
| **speech_ratio_neg** | 0.347 | 0.079 | 0.386 | 0.087 | 0.461 | 0.076 | 0.44 | 0.096 | -1.935 | 0.022 | 0.257 |
| **apq5_shimmer_pos** | 7.453 | 1.387 | 8.995 | 2.232 | 10.352 | 1.76 | 10.289 | 3.318 | -1.904 | 0.021 | 0.261 |
| **f2_bandwidth_sd_pos** | 378.089 | 56.135 | 346.988 | 43.734 | 361.486 | 65.843 | 371.394 | 75.115 | 1.913 | 0.027 | 0.261 |
| **adverb_rate_neg** | 0.164 | 0.074 | 0.186 | 0.063 | 0.208 | 0.055 | 0.183 | 0.069 | -1.874 | 0.026 | 0.264 |
| **spectral_slope_500_1500_mean_pos** | -10.345 | 2.213 | -9.673 | 2.363 | -9.84 | 1.644 | -10.621 | 2.499 | -1.877 | 0.024 | 0.264 |
| **local_jitter_pos** | 2.052 | 0.379 | 2.353 | 0.629 | 2.937 | 0.563 | 2.816 | 0.695 | -1.858 | 0.019 | 0.267 |
| **verb_phrase_with_aux_rate_pos** | 0.528 | 0.362 | 0.617 | 0.35 | 0.47 | 0.255 | 0.351 | 0.208 | -1.723 | 0.021 | 0.347 |
| **average_mfccs_3_pos** | 14.977 | 5.058 | 20.357 | 6.393 | 24.398 | 8.027 | 25.888 | 5.289 | -1.71 | 0.014 | 0.348 |
| **f1_relative_energy_mean_pos** | 1.112 | 4.675 | -0.435 | 4.371 | -1.249 | 2.975 | -0.599 | 3.639 | 1.648 | 0.018 | 0.386 |
| **utterance_durations_sd_neg** | 0.109 | 0.032 | 0.137 | 0.044 | 0.213 | 0.049 | 0.211 | 0.078 | -1.601 | 0.01 | 0.414 |
| **mean_number_subordinate_clauses_pos** | 0.994 | 1.163 | 1.825 | 1.84 | 1.759 | 2.207 | 1.683 | 2.097 | -1.573 | 0.017 | 0.429 |
| **proportion_verb_phrase_with_subjects_neg** | 0.802 | 0.163 | 0.842 | 0.108 | 0.814 | 0.172 | 0.76 | 0.297 | -1.56 | 0.018 | 0.43 |
| **f2_bandwidth_mean_pos** | 510.301 | 90.169 | 476.276 | 93.772 | 529.237 | 113.247 | 549.443 | 90.712 | 1.543 | 0.017 | 0.435 |
| **f3_relative_energy_sd_pos** | 9.479 | 1.349 | 8.91 | 1.316 | 8.584 | 0.857 | 8.667 | 1.085 | 1.509 | 0.016 | 0.454 |
| **adverb_rate_pos** | 0.147 | 0.074 | 0.179 | 0.068 | 0.186 | 0.053 | 0.174 | 0.079 | -1.459 | 0.016 | 0.469 |
| **proper_noun_rate_neg** | 0.012 | 0.036 | 0.004 | 0.008 | 0.004 | 0.007 | 0.007 | 0.024 | 1.44 | 0.016 | 0.469 |
| **alpha_ratio_mean_neg** | 7.495 | 2.348 | 8.008 | 2.249 | 7.04 | 2.688 | 8.85 | 2.979 | 1.45 | 0.015 | 0.469 |
| **type_token_ratio_neg** | 0.78 | 0.129 | 0.651 | 0.17 | 0.684 | 0.103 | 0.648 | 0.194 | 1.476 | 0.014 | 0.469 |
| **noun_rate_pos** | 0.142 | 0.056 | 0.113 | 0.048 | 0.122 | 0.046 | 0.121 | 0.072 | 1.44 | 0.015 | 0.469 |
| **f3_frequency_mean_neg** | 2751.594 | 143.194 | 2725.263 | 127.255 | 2718.105 | 98.854 | 2765.805 | 110.799 | 1.414 | 0.013 | 0.482 |
| **loudness_mean_neg** | -38.77 | 4.126 | -36.791 | 5.205 | -36.027 | 3.489 | -36.446 | 3.582 | -1.291 | 0.012 | 0.586 |
| **pronoun_rate_pos** | 0.119 | 0.06 | 0.13 | 0.038 | 0.137 | 0.029 | 0.126 | 0.057 | -1.285 | 0.012 | 0.586 |
| **f3_relative_energy_mean_pos** | -21.115 | 6.19 | -23.357 | 5.029 | -22.593 | 3.533 | -23.185 | 4.107 | 1.097 | 0.007 | 0.597 |
| **local_absolute_jitter_pos** | 0.013 | 0.006 | 0.016 | 0.006 | 0.02 | 0.007 | 0.02 | 0.007 | -1.209 | 0.005 | 0.597 |
| **f2_frequency_mean_neg** | 1632.556 | 114.923 | 1635.014 | 94.582 | 1641.943 | 81.156 | 1686.774 | 130.307 | 1.192 | 0.009 | 0.597 |
| **rate_loudness_peaks_pos** | 6.768 | 1.121 | 6.134 | 1.123 | 6.046 | 1.138 | 5.88 | 1.508 | 1.16 | 0.008 | 0.597 |
| **f2_bandwidth_sd_neg** | 370.839 | 57.202 | 341.279 | 42.398 | 351.556 | 44.887 | 346.377 | 83.97 | 1.133 | 0.009 | 0.597 |
| **pronoun_rate_neg** | 0.119 | 0.044 | 0.139 | 0.041 | 0.129 | 0.045 | 0.127 | 0.053 | -1.113 | 0.009 | 0.597 |
| **spectral_slope_0_500_mean_pos** | 2.908 | 1.636 | 2.075 | 2.224 | 2.264 | 2.249 | 2.19 | 2.291 | 1.089 | 0.007 | 0.597 |
| **pause_rate_pos** | 0.556 | 0.104 | 0.507 | 0.13 | 0.502 | 0.099 | 0.502 | 0.168 | 1.092 | 0.009 | 0.597 |
| **alpha_ratio_sd_neg** | 6.252 | 2.275 | 6.581 | 2.146 | 5.549 | 2.293 | 6.899 | 2.724 | 1.091 | 0.009 | 0.597 |
| **hammarberg_index_sd_neg** | 7.906 | 1.549 | 7.893 | 1.443 | 7.219 | 0.93 | 7.939 | 1.804 | 1.241 | 0.011 | 0.597 |
| **alpha_ratio_sd_pos** | 6.67 | 2.739 | 6.404 | 2.061 | 6.155 | 2.309 | 6.873 | 2.91 | 1.083 | 0.009 | 0.597 |
| **pause_rate_neg** | 0.556 | 0.103 | 0.531 | 0.109 | 0.481 | 0.087 | 0.501 | 0.155 | 1.123 | 0.009 | 0.597 |
| **conjunction_rate_neg** | 0.077 | 0.039 | 0.08 | 0.026 | 0.08 | 0.028 | 0.073 | 0.033 | -1.212 | 0.01 | 0.597 |
| **conjunction_rate_pos** | 0.063 | 0.037 | 0.073 | 0.035 | 0.066 | 0.039 | 0.061 | 0.035 | -1.161 | 0.009 | 0.597 |
| **local_absolute_jitter_neg** | 0.014 | 0.007 | 0.017 | 0.006 | 0.02 | 0.008 | 0.02 | 0.008 | -1.187 | 0.005 | 0.597 |
| **f1_relative_energy_sd_pos** | 7.206 | 1.198 | 7.008 | 1.046 | 7.987 | 0.896 | 7.191 | 1.06 | -1.185 | 0.008 | 0.597 |
| **perceived_loudness_mean_neg** | -39.325 | 4.144 | -37.468 | 5.334 | -36.688 | 3.488 | -37.147 | 3.58 | -1.228 | 0.011 | 0.597 |
| **pitch_std_neg** | 31.149 | 11.596 | 28.84 | 12.474 | 30.872 | 9.898 | 25.76 | 9.475 | -1.127 | 0.006 | 0.597 |
| **proportion_verb_phrase_with_objects_neg** | 0.269 | 0.196 | 0.279 | 0.098 | 0.319 | 0.133 | 0.254 | 0.133 | -1.261 | 0.012 | 0.597 |
| **hammarberg_index_sd_pos** | 8.141 | 1.789 | 7.889 | 1.439 | 7.223 | 0.732 | 7.77 | 1.6 | 1.189 | 0.01 | 0.597 |
| **h1_h2_harmonic_difference_sd_pos** | 8.31 | 1.687 | 7.374 | 1.51 | 7.94 | 1.047 | 7.545 | 1.304 | 1.072 | 0.006 | 0.597 |
| **verb_phrase_with_aux_and_vp_rate_pos** | 0.054 | 0.164 | 0.034 | 0.076 | 0.025 | 0.046 | 0.037 | 0.08 | 1.08 | 0.008 | 0.597 |
| **f2_frequency_sd_pos** | 339.401 | 49.704 | 350.572 | 79.612 | 355.348 | 49.943 | 384.94 | 66.848 | 0.989 | 0.006 | 0.669 |
| **adjective_rate_neg** | 0.045 | 0.037 | 0.043 | 0.02 | 0.054 | 0.024 | 0.044 | 0.031 | -0.949 | 0.007 | 0.702 |
| **utterance_durations_mean_neg** | 0.127 | 0.026 | 0.153 | 0.036 | 0.193 | 0.036 | 0.206 | 0.042 | -0.938 | 0.004 | 0.704 |
| **pause_durations_sum_pos** | 11.697 | 9.059 | 24.56 | 28.145 | 21.662 | 17.396 | 44.344 | 54.479 | 0.915 | 0.005 | 0.706 |
| **utterance_durations_mean_pos** | 0.124 | 0.024 | 0.156 | 0.042 | 0.186 | 0.041 | 0.203 | 0.049 | -0.914 | 0.004 | 0.706 |
| **pitch_mean_neg** | 160.681 | 41.3 | 153.062 | 37.931 | 149.335 | 33.082 | 141.495 | 36.172 | -0.911 | 0.001 | 0.706 |
| **f1_relative_energy_mean_neg** | 1.037 | 4.391 | -0.62 | 3.905 | -0.62 | 3.33 | -1.199 | 4.089 | 0.896 | 0.005 | 0.71 |
| **adposition_rate_neg** | 0.059 | 0.037 | 0.063 | 0.033 | 0.055 | 0.034 | 0.049 | 0.03 | -0.883 | 0.006 | 0.71 |
| **f1_bandwidth_sd_pos** | 238.33 | 51.33 | 231.57 | 49.254 | 233.631 | 38.361 | 241.108 | 46.223 | 0.889 | 0.005 | 0.71 |
| **type_token_ratio_pos** | 0.808 | 0.122 | 0.727 | 0.151 | 0.682 | 0.116 | 0.652 | 0.169 | 0.865 | 0.005 | 0.72 |
| **verb_rate_pos** | 0.1 | 0.042 | 0.098 | 0.035 | 0.096 | 0.026 | 0.108 | 0.052 | 0.818 | 0.005 | 0.759 |
| **honore_stat_pos** | 2783.282 | 1883.196 | 2799.704 | 1781.18 | 2507.536 | 1260.293 | 2212.022 | 1006.95 | -0.811 | 0.005 | 0.759 |
| **determiner_rate_neg** | 0.087 | 0.036 | 0.085 | 0.031 | 0.082 | 0.031 | 0.07 | 0.035 | -0.795 | 0.005 | 0.767 |
| **alpha_ratio_mean_pos** | 7.408 | 2.376 | 7.876 | 2.518 | 7.899 | 2.95 | 8.999 | 3.374 | 0.774 | 0.004 | 0.778 |
| **average_mfccs_2_neg** | 13.074 | 10.142 | 14.245 | 11.291 | 5.767 | 10.107 | 9.447 | 7.677 | 0.768 | 0.004 | 0.778 |
| **hammarberg_index_mean_neg** | 24.29 | 3.066 | 25.724 | 3.066 | 23.773 | 2.842 | 24.497 | 2.534 | -0.73 | 0.004 | 0.801 |
| **hammarberg_index_mean_pos** | 23.913 | 3.434 | 25.712 | 3.204 | 24.582 | 2.659 | 25.413 | 3.654 | -0.733 | 0.004 | 0.801 |
| **f2_relative_energy_mean_pos** | -13.124 | 5.598 | -14.529 | 5.109 | -15.907 | 3.878 | -16.376 | 3.683 | 0.712 | 0.003 | 0.811 |
| **f1_frequency_mean_neg** | 586.532 | 56.186 | 582.593 | 50.229 | 563.709 | 41.398 | 543.349 | 82.028 | -0.673 | 0.003 | 0.828 |
| **number_consecutive_repetitions_neg** | 0.038 | 0.194 | 0.455 | 1.19 | 0.316 | 0.749 | 0.45 | 0.945 | -0.672 | 0.003 | 0.828 |
| **loudness_mean_pos** | -38.015 | 4.294 | -36.181 | 5.412 | -36.057 | 3.662 | -35.937 | 3.386 | -0.684 | 0.003 | 0.828 |
| **honore_stat_neg** | 2873.98 | 1484.126 | 2382.261 | 977.431 | 2537.013 | 788.01 | 1875.824 | 575.838 | -0.652 | 0.003 | 0.832 |
| **verb_rate_neg** | 0.117 | 0.035 | 0.106 | 0.02 | 0.098 | 0.032 | 0.095 | 0.037 | 0.654 | 0.003 | 0.832 |
| **pitch_range_pos** | 270.869 | 153.987 | 231.805 | 136.015 | 284.227 | 67.672 | 286.421 | 153.564 | 0.625 | 0.003 | 0.845 |
| **proportion_verb_phrase_with_subjects_pos** | 0.782 | 0.218 | 0.813 | 0.15 | 0.805 | 0.137 | 0.807 | 0.235 | -0.624 | 0.003 | 0.845 |
| **f3_relative_energy_mean_neg** | -21.286 | 5.534 | -23.293 | 4.799 | -21.539 | 3.875 | -22.915 | 3.409 | 0.61 | 0.002 | 0.846 |
| **duration_pos** | 19.962 | 13.032 | 43.158 | 44.167 | 42.747 | 33.032 | 76.374 | 81.006 | 0.607 | 0.002 | 0.846 |
| **f1_frequency_sd_neg** | 225.617 | 72.705 | 256.503 | 86.713 | 218.831 | 57.977 | 227.515 | 76.153 | -0.591 | 0.002 | 0.847 |
| **perceived_loudness_mean_pos** | -38.515 | 4.301 | -36.843 | 5.522 | -36.771 | 3.665 | -36.654 | 3.4 | -0.59 | 0.002 | 0.847 |
| **f1_bandwidth_sd_neg** | 230.853 | 57.431 | 235.936 | 44.796 | 226.858 | 32.586 | 241.158 | 52.688 | 0.545 | 0.002 | 0.853 |
| **number_of_pauses_neg** | 72.808 | 51.78 | 189.773 | 221.073 | 98.474 | 50.452 | 184.0 | 288.121 | -0.548 | 0.002 | 0.853 |
| **f3_bandwidth_sd_pos** | 367.988 | 61.866 | 377.194 | 81.09 | 337.955 | 52.693 | 340.616 | 64.687 | -0.534 | 0.002 | 0.853 |
| **f1_bandwidth_mean_pos** | 304.197 | 78.773 | 311.696 | 80.156 | 324.94 | 62.933 | 318.441 | 70.031 | -0.523 | 0.002 | 0.853 |
| **f3_bandwidth_sd_neg** | 363.853 | 61.178 | 367.418 | 55.1 | 338.991 | 39.369 | 335.624 | 79.2 | -0.554 | 0.002 | 0.853 |
| **spectral_slope_0_500_sd_pos** | 2.326 | 0.296 | 2.278 | 0.327 | 2.54 | 0.477 | 2.5 | 0.366 | 0.56 | 0.002 | 0.853 |
| **h1_h2_harmonic_difference_mean_neg** | 4.306 | 3.823 | 5.25 | 2.933 | 4.174 | 2.272 | 5.979 | 3.489 | 0.577 | 0.002 | 0.853 |
| **pitch_std_pos** | 35.94 | 17.343 | 27.801 | 12.836 | 37.634 | 11.935 | 32.831 | 13.38 | 0.529 | 0.002 | 0.853 |
| **pitch_range_neg** | 247.941 | 128.129 | 250.733 | 157.303 | 249.113 | 90.537 | 234.049 | 113.316 | -0.443 | 0.001 | 0.855 |
| **number_consecutive_repetitions_pos** | 0.115 | 0.323 | 0.318 | 0.74 | 0.316 | 0.671 | 0.4 | 0.681 | -0.448 | 0.001 | 0.855 |
| **utterance_durations_sum_neg** | 9.338 | 6.395 | 29.296 | 35.212 | 18.675 | 8.859 | 34.377 | 42.9 | -0.481 | 0.002 | 0.855 |
| **f3_frequency_sd_pos** | 277.694 | 59.022 | 307.279 | 64.973 | 310.112 | 80.235 | 322.249 | 61.241 | -0.485 | 0.001 | 0.855 |
| **proper_noun_rate_pos** | 0.016 | 0.028 | 0.015 | 0.023 | 0.006 | 0.01 | 0.005 | 0.011 | -0.453 | 0.001 | 0.855 |
| **rate_loudness_peaks_neg** | 6.63 | 1.033 | 6.266 | 1.003 | 6.08 | 0.741 | 5.515 | 0.984 | -0.455 | 0.001 | 0.855 |
| **proportion_verb_phrase_with_objects_pos** | 0.319 | 0.18 | 0.273 | 0.09 | 0.338 | 0.15 | 0.321 | 0.149 | 0.472 | 0.002 | 0.855 |
| **inflected_verb_rate_pos** | 0.7 | 0.287 | 0.705 | 0.294 | 0.708 | 0.187 | 0.682 | 0.256 | -0.459 | 0.002 | 0.855 |
| **word_count_neg** | 61.846 | 47.906 | 171.295 | 201.942 | 100.579 | 52.556 | 184.1 | 292.716 | -0.461 | 0.001 | 0.855 |
| **verb_phrase_with_aux_and_vp_rate_neg** | 0.037 | 0.086 | 0.033 | 0.057 | 0.009 | 0.029 | 0.014 | 0.03 | 0.439 | 0.001 | 0.855 |
| **spectral_slope_500_1500_sd_pos** | 5.447 | 0.799 | 5.473 | 1.008 | 5.501 | 0.625 | 5.264 | 0.675 | -0.489 | 0.002 | 0.855 |
| **adjective_rate_pos** | 0.068 | 0.05 | 0.055 | 0.033 | 0.062 | 0.029 | 0.055 | 0.045 | 0.424 | 0.001 | 0.863 |
| **f3_bandwidth_mean_neg** | 568.165 | 97.658 | 583.416 | 97.403 | 538.561 | 96.611 | 545.111 | 102.024 | -0.408 | 0.001 | 0.867 |
| **h1_a3_harmonic_difference_sd_neg** | 8.421 | 1.289 | 8.453 | 1.244 | 8.194 | 0.765 | 8.407 | 1.689 | 0.391 | 0.001 | 0.867 |
| **f2_frequency_sd_neg** | 345.067 | 53.089 | 354.709 | 65.573 | 353.665 | 49.318 | 370.551 | 102.553 | 0.394 | 0.001 | 0.867 |
| **f1_relative_energy_sd_neg** | 7.47 | 1.318 | 7.22 | 1.132 | 7.378 | 0.567 | 6.857 | 0.928 | -0.402 | 0.001 | 0.867 |
| **f3_bandwidth_mean_pos** | 575.217 | 113.414 | 596.023 | 101.957 | 551.473 | 93.405 | 564.248 | 89.504 | -0.37 | 0.001 | 0.874 |
| **f3_frequency_mean_pos** | 2744.076 | 133.429 | 2707.736 | 134.169 | 2747.219 | 112.449 | 2748.035 | 109.592 | 0.368 | 0.001 | 0.874 |
| **f2_relative_energy_sd_neg** | 9.684 | 1.531 | 9.131 | 1.365 | 8.656 | 0.715 | 8.301 | 1.543 | 0.35 | 0.001 | 0.884 |
| **pitch_mean_pos** | 171.198 | 45.914 | 155.995 | 40.16 | 153.814 | 38.162 | 146.861 | 34.893 | 0.338 | 0.0 | 0.888 |
| **adposition_rate_pos** | 0.071 | 0.04 | 0.074 | 0.042 | 0.054 | 0.025 | 0.056 | 0.029 | -0.298 | 0.001 | 0.897 |
| **duration_neg** | 27.06 | 18.703 | 78.604 | 95.087 | 41.223 | 20.059 | 85.048 | 124.503 | -0.317 | 0.001 | 0.897 |
| **h1_h2_harmonic_difference_mean_pos** | 4.203 | 4.165 | 5.065 | 2.99 | 4.282 | 2.015 | 5.801 | 3.483 | 0.305 | 0.001 | 0.897 |
| **f1_frequency_sd_pos** | 229.222 | 68.59 | 253.291 | 92.649 | 219.31 | 56.228 | 233.108 | 57.154 | -0.292 | 0.001 | 0.897 |
| **f3_frequency_sd_neg** | 281.721 | 58.115 | 310.932 | 67.153 | 299.985 | 62.905 | 316.197 | 90.229 | -0.309 | 0.001 | 0.897 |
| **f2_frequency_mean_pos** | 1636.824 | 96.884 | 1622.083 | 102.559 | 1632.733 | 92.436 | 1636.69 | 104.534 | 0.275 | 0.001 | 0.904 |
| **average_mfccs_2_pos** | 9.555 | 10.703 | 11.961 | 12.156 | 6.955 | 10.351 | 8.274 | 7.067 | -0.263 | 0.0 | 0.904 |
| **h1_h2_harmonic_difference_sd_neg** | 8.068 | 1.321 | 7.327 | 1.397 | 7.946 | 0.885 | 7.411 | 1.28 | 0.268 | 0.0 | 0.904 |
| **number_of_pauses_pos** | 54.865 | 38.677 | 105.932 | 100.685 | 102.737 | 75.606 | 166.4 | 173.705 | 0.247 | 0.0 | 0.912 |
| **spectral_slope_500_1500_sd_neg** | 5.496 | 0.723 | 5.385 | 0.927 | 5.47 | 0.721 | 5.221 | 0.956 | -0.23 | 0.0 | 0.92 |
| **h1_a3_harmonic_difference_sd_pos** | 8.617 | 1.331 | 8.364 | 1.261 | 8.73 | 0.921 | 8.555 | 0.958 | 0.205 | 0.0 | 0.929 |
| **f1_bandwidth_mean_neg** | 295.917 | 69.203 | 313.84 | 74.747 | 305.102 | 54.798 | 317.262 | 79.927 | -0.183 | 0.0 | 0.929 |
| **f2_relative_energy_mean_neg** | -13.289 | 5.499 | -14.818 | 4.725 | -14.694 | 3.753 | -16.794 | 3.808 | -0.199 | 0.0 | 0.929 |
| **noun_rate_neg** | 0.123 | 0.039 | 0.111 | 0.04 | 0.112 | 0.037 | 0.107 | 0.07 | 0.195 | 0.0 | 0.929 |
| **f3_relative_energy_sd_neg** | 9.088 | 1.529 | 9.099 | 1.454 | 8.498 | 0.553 | 8.416 | 1.758 | -0.19 | 0.0 | 0.929 |
| **h1_a3_harmonic_difference_mean_pos** | -19.263 | 3.593 | -21.547 | 3.755 | -19.416 | 3.612 | -21.481 | 3.94 | 0.137 | 0.0 | 0.962 |
| **utterance_durations_sum_pos** | 6.706 | 4.34 | 16.329 | 16.611 | 19.773 | 17.234 | 30.596 | 27.82 | 0.119 | 0.0 | 0.967 |
| **pause_durations_sum_neg** | 15.87 | 12.988 | 45.913 | 59.645 | 20.714 | 12.495 | 49.258 | 82.78 | -0.116 | 0.0 | 0.967 |
| **h1_a3_harmonic_difference_mean_neg** | -19.729 | 3.486 | -21.618 | 3.789 | -19.084 | 3.585 | -21.103 | 3.772 | -0.103 | 0.0 | 0.972 |
| **average_mfccs_3_neg** | 16.165 | 4.759 | 21.259 | 6.849 | 24.257 | 7.755 | 28.686 | 7.789 | -0.071 | 0.0 | 0.973 |
| **spectral_slope_0_500_mean_neg** | 2.537 | 1.531 | 1.948 | 2.041 | 2.407 | 2.006 | 1.916 | 2.422 | 0.073 | 0.0 | 0.973 |
| **inflected_verb_rate_neg** | 0.803 | 0.21 | 0.8 | 0.148 | 0.717 | 0.244 | 0.694 | 0.276 | -0.087 | 0.0 | 0.973 |
| **spectral_slope_0_500_sd_neg** | 2.344 | 0.285 | 2.257 | 0.304 | 2.463 | 0.423 | 2.357 | 0.269 | -0.09 | 0.0 | 0.973 |
| **f1_frequency_mean_pos** | 599.088 | 63.779 | 584.866 | 51.914 | 556.068 | 34.655 | 543.175 | 51.768 | 0.033 | 0.0 | 0.98 |
| **determiner_rate_pos** | 0.1 | 0.051 | 0.086 | 0.031 | 0.084 | 0.024 | 0.068 | 0.028 | -0.053 | 0.0 | 0.98 |
| **verb_phrase_with_aux_rate_neg** | 0.541 | 0.351 | 0.535 | 0.18 | 0.412 | 0.225 | 0.399 | 0.244 | -0.04 | 0.0 | 0.98 |
| **word_count_pos** | 48.231 | 33.184 | 97.5 | 109.999 | 106.474 | 92.377 | 159.2 | 175.345 | 0.045 | 0.0 | 0.98 |
| **mean_number_subordinate_clauses_neg** | 1.763 | 1.77 | 1.208 | 1.368 | 1.452 | 1.888 | 0.868 | 1.297 | 0.003 | 0.0 | 0.998 |

**Supp. Table S4:** Partial correlations *r* between speech features and the BDI-II. pos=positive story; neg=negative story; *p*=unadjusted *p* value; *r*=correlation coefficient; Adj. *p*=*p* value adjusted for multiple hypothesis testing according to the Benjamini-Hochberg procedure.

| **Speech feature** | **r** | ***p*** | **adj. *p*** |
| --- | --- | --- | --- |
| **utterance_durations_sum_neg** | 0.32 | <0.001 | <0.001 |
| **duration_neg** | 0.32 | <0.001 | <0.001 |
| **word_count_neg** | 0.32 | <0.001 | <0.001 |
| **average_mfccs_1_pos** | 0.33 | <0.001 | <0.001 |
| **dda_shimmer_pos** | 0.34 | <0.001 | <0.001 |
| **apq3_shimmer_pos** | 0.34 | <0.001 | <0.001 |
| **apq5_shimmer_pos** | 0.37 | <0.001 | <0.001 |
| **f2_bandwidth_sd_neg** | -0.30 | 0.001 | 0.015 |
| **average_mfccs_3_neg** | 0.30 | 0.001 | 0.015 |
| **pause_durations_sum_neg** | 0.31 | 0.001 | 0.015 |
| **number_of_pauses_neg** | 0.31 | 0.001 | 0.015 |
| **type_token_ratio_neg** | -0.28 | 0.002 | 0.027 |
| **utterance_durations_mean_pos** | 0.27 | 0.003 | 0.035 |
| **local_jitter_pos** | 0.27 | 0.003 | 0.035 |
| **local_shimmer_pos** | 0.26 | 0.004 | 0.041 |
| **number_consecutive_repetitions_neg** | 0.26 | 0.004 | 0.041 |
| **loudness_sd_pos** | -0.26 | 0.005 | 0.048 |
| **rap_jitter_pos** | 0.25 | 0.006 | 0.049 |
| **ddp_jitter_pos** | 0.25 | 0.006 | 0.049 |
| **local_dB_shimmer_pos** | 0.25 | 0.006 | 0.049 |
| **f2_bandwidth_sd_pos** | -0.25 | 0.007 | 0.052 |
| **noun_rate_pos** | -0.24 | 0.007 | 0.052 |
| **verb_rate_neg** | -0.24 | 0.008 | 0.057 |
| **mean_number_subordinate_clauses_neg** | -0.24 | 0.01 | 0.068 |
| **hammarberg_index_mean_pos** | 0.22 | 0.014 | 0.091 |
| **duration_pos** | 0.22 | 0.015 | 0.094 |
| **average_mfccs_1_neg** | 0.22 | 0.017 | 0.103 |
| **perceived_loudness_sd_pos** | -0.22 | 0.018 | 0.105 |
| **f1_bandwidth_sd_pos** | -0.21 | 0.02 | 0.105 |
| **ppq5_jitter_pos** | 0.21 | 0.02 | 0.105 |
| **pause_durations_sum_pos** | 0.21 | 0.02 | 0.105 |
| **number_of_pauses_pos** | 0.20 | 0.026 | 0.132 |
| **word_count_pos** | 0.20 | 0.027 | 0.133 |
| **utterance_durations_sum_pos** | 0.20 | 0.028 | 0.134 |
| **apq11_shimmer_neg** | 0.20 | 0.029 | 0.135 |
| **average_mfccs_3_pos** | 0.20 | 0.033 | 0.149 |
| **hnr_sd_pos** | -0.19 | 0.04 | 0.176 |
| **pause_rate_pos** | -0.19 | 0.041 | 0.176 |
| **apq5_shimmer_neg** | 0.18 | 0.055 | 0.23 |
| **f2_bandwidth_mean_neg** | -0.17 | 0.059 | 0.24 |
| **f2_relative_energy_sd_pos** | -0.17 | 0.063 | 0.25 |
| **speech_ratio_pos** | 0.17 | 0.066 | 0.256 |
| **f3_relative_energy_mean_pos** | -0.17 | 0.07 | 0.259 |
| **spectral_slope_0_500_sd_neg** | -0.17 | 0.07 | 0.259 |
| **proportion_verb_phrase_with_objects_pos** | -0.16 | 0.074 | 0.268 |
| **loudness_mean_neg** | 0.16 | 0.08 | 0.283 |
| **f1_frequency_mean_neg** | -0.16 | 0.082 | 0.284 |
| **f1_bandwidth_sd_neg** | -0.16 | 0.086 | 0.292 |
| **h1_a3_harmonic_difference_mean_pos** | -0.16 | 0.091 | 0.3 |
| **perceived_loudness_mean_neg** | 0.15 | 0.092 | 0.3 |
| **hnr_mean_pos** | -0.15 | 0.098 | 0.313 |
| **hammarberg_index_sd_neg** | -0.15 | 0.102 | 0.32 |
| **adjective_rate_pos** | -0.15 | 0.109 | 0.329 |
| **utterance_durations_mean_neg** | 0.15 | 0.109 | 0.329 |
| **hnr_sd_neg** | -0.14 | 0.12 | 0.343 |
| **hammarberg_index_sd_pos** | -0.14 | 0.118 | 0.343 |
| **type_token_ratio_pos** | -0.14 | 0.122 | 0.343 |
| **f3_bandwidth_mean_pos** | 0.14 | 0.116 | 0.343 |
| **spectral_slope_500_1500_sd_neg** | -0.14 | 0.128 | 0.348 |
| **h1_h2_harmonic_difference_sd_pos** | -0.14 | 0.128 | 0.348 |
| **loudness_sd_neg** | -0.14 | 0.131 | 0.35 |
| **determiner_rate_pos** | -0.13 | 0.147 | 0.381 |
| **dda_shimmer_neg** | 0.13 | 0.152 | 0.381 |
| **apq3_shimmer_neg** | 0.13 | 0.152 | 0.381 |
| **average_mfccs_4_neg** | 0.13 | 0.148 | 0.381 |
| **f2_frequency_mean_pos** | -0.13 | 0.159 | 0.393 |
| **noun_rate_neg** | -0.13 | 0.164 | 0.393 |
| **local_jitter_neg** | 0.13 | 0.163 | 0.393 |
| **spectral_slope_0_500_sd_pos** | -0.13 | 0.17 | 0.402 |
| **loudness_mean_pos** | 0.12 | 0.178 | 0.414 |
| **honore_stat_neg** | -0.12 | 0.188 | 0.43 |
| **verb_phrase_with_aux_rate_neg** | -0.12 | 0.202 | 0.43 |
| **f1_frequency_mean_pos** | -0.12 | 0.202 | 0.43 |
| **f2_bandwidth_mean_pos** | -0.12 | 0.211 | 0.43 |
| **perceived_loudness_sd_neg** | -0.12 | 0.211 | 0.43 |
| **adverb_rate_pos** | 0.12 | 0.21 | 0.43 |
| **ppq5_jitter_neg** | 0.12 | 0.208 | 0.43 |
| **utterance_durations_sd_pos** | 0.12 | 0.202 | 0.43 |
| **ddp_jitter_neg** | 0.12 | 0.204 | 0.43 |
| **rap_jitter_neg** | 0.12 | 0.204 | 0.43 |
| **perceived_loudness_mean_pos** | 0.11 | 0.217 | 0.437 |
| **f1_relative_energy_mean_pos** | -0.11 | 0.229 | 0.455 |
| **rate_loudness_peaks_neg** | -0.11 | 0.247 | 0.485 |
| **local_shimmer_neg** | 0.11 | 0.252 | 0.489 |
| **proper_noun_rate_neg** | -0.10 | 0.257 | 0.491 |
| **h1_a3_harmonic_difference_sd_neg** | -0.10 | 0.265 | 0.491 |
| **h1_a3_harmonic_difference_sd_pos** | -0.10 | 0.266 | 0.491 |
| **local_dB_shimmer_neg** | 0.10 | 0.268 | 0.491 |
| **local_absolute_jitter_pos** | 0.10 | 0.26 | 0.491 |
| **verb_phrase_with_aux_and_vp_rate_pos** | -0.10 | 0.277 | 0.502 |
| **hammarberg_index_mean_neg** | 0.10 | 0.295 | 0.523 |
| **average_mfccs_4_pos** | 0.10 | 0.294 | 0.523 |
| **f3_relative_energy_sd_pos** | -0.09 | 0.307 | 0.532 |
| **f1_relative_energy_sd_pos** | -0.09 | 0.305 | 0.532 |
| **average_mfccs_2_neg** | 0.09 | 0.31 | 0.532 |
| **verb_rate_pos** | -0.09 | 0.32 | 0.543 |
| **alpha_ratio_mean_pos** | 0.09 | 0.324 | 0.544 |
| **f1_frequency_sd_neg** | -0.09 | 0.329 | 0.547 |
| **inflected_verb_rate_pos** | -0.09 | 0.341 | 0.552 |
| **f1_bandwidth_mean_neg** | -0.09 | 0.343 | 0.552 |
| **f2_relative_energy_sd_neg** | -0.09 | 0.344 | 0.552 |
| **f2_relative_energy_mean_pos** | -0.09 | 0.351 | 0.552 |
| **alpha_ratio_sd_pos** | -0.09 | 0.352 | 0.552 |
| **average_mfccs_2_pos** | 0.09 | 0.352 | 0.552 |
| **f2_frequency_sd_neg** | -0.08 | 0.359 | 0.553 |
| **f3_bandwidth_sd_neg** | -0.08 | 0.363 | 0.553 |
| **h1_h2_harmonic_difference_sd_neg** | -0.08 | 0.361 | 0.553 |
| **f3_frequency_sd_pos** | 0.08 | 0.372 | 0.561 |
| **h1_h2_harmonic_difference_mean_neg** | 0.08 | 0.4 | 0.598 |
| **pause_rate_neg** | -0.08 | 0.41 | 0.602 |
| **speech_ratio_neg** | 0.08 | 0.408 | 0.602 |
| **h1_a3_harmonic_difference_mean_neg** | -0.07 | 0.424 | 0.612 |
| **pitch_range_pos** | -0.07 | 0.428 | 0.612 |
| **conjunction_rate_pos** | 0.07 | 0.421 | 0.612 |
| **f3_relative_energy_mean_neg** | -0.07 | 0.449 | 0.631 |
| **pitch_std_pos** | -0.07 | 0.447 | 0.631 |
| **pitch_std_neg** | 0.07 | 0.458 | 0.638 |
| **spectral_slope_0_500_mean_neg** | 0.07 | 0.478 | 0.66 |
| **spectral_slope_0_500_mean_pos** | -0.06 | 0.499 | 0.682 |
| **mean_number_subordinate_clauses_pos** | 0.06 | 0.502 | 0.682 |
| **pitch_mean_neg** | 0.06 | 0.518 | 0.698 |
| **inflected_verb_rate_neg** | -0.06 | 0.536 | 0.716 |
| **alpha_ratio_mean_neg** | 0.06 | 0.545 | 0.722 |
| **number_consecutive_repetitions_pos** | -0.06 | 0.552 | 0.726 |
| **adposition_rate_neg** | 0.05 | 0.561 | 0.732 |
| **f1_frequency_sd_pos** | -0.05 | 0.603 | 0.78 |
| **f2_relative_energy_mean_neg** | -0.05 | 0.617 | 0.792 |
| **f2_frequency_sd_pos** | -0.04 | 0.643 | 0.809 |
| **pitch_range_neg** | 0.04 | 0.64 | 0.809 |
| **h1_h2_harmonic_difference_mean_pos** | 0.04 | 0.645 | 0.809 |
| **pronoun_rate_pos** | -0.04 | 0.664 | 0.82 |
| **f1_relative_energy_mean_neg** | -0.04 | 0.661 | 0.82 |
| **f3_frequency_sd_neg** | -0.04 | 0.688 | 0.843 |
| **utterance_durations_sd_neg** | 0.04 | 0.694 | 0.844 |
| **verb_phrase_with_aux_rate_pos** | 0.04 | 0.699 | 0.844 |
| **f2_frequency_mean_neg** | 0.04 | 0.707 | 0.847 |
| **f3_frequency_mean_neg** | 0.03 | 0.718 | 0.854 |
| **f3_relative_energy_sd_neg** | -0.03 | 0.733 | 0.857 |
| **proper_noun_rate_pos** | -0.03 | 0.74 | 0.857 |
| **verb_phrase_with_aux_and_vp_rate_neg** | -0.03 | 0.741 | 0.857 |
| **pronoun_rate_neg** | 0.03 | 0.738 | 0.857 |
| **conjunction_rate_neg** | 0.03 | 0.751 | 0.862 |
| **determiner_rate_neg** | -0.03 | 0.76 | 0.864 |
| **f1_bandwidth_mean_pos** | -0.03 | 0.763 | 0.864 |
| **f1_relative_energy_sd_neg** | -0.02 | 0.817 | 0.894 |
| **spectral_slope_500_1500_sd_pos** | -0.02 | 0.822 | 0.894 |
| **spectral_slope_500_1500_mean_neg** | -0.02 | 0.828 | 0.894 |
| **adposition_rate_pos** | 0.02 | 0.826 | 0.894 |
| **spectral_slope_500_1500_mean_pos** | 0.02 | 0.817 | 0.894 |
| **f3_bandwidth_mean_neg** | 0.02 | 0.815 | 0.894 |
| **hnr_mean_neg** | 0.02 | 0.801 | 0.894 |
| **adverb_rate_neg** | 0.02 | 0.849 | 0.91 |
| **f3_frequency_mean_pos** | -0.02 | 0.859 | 0.915 |
| **f3_bandwidth_sd_pos** | 0.01 | 0.894 | 0.946 |
| **adjective_rate_neg** | -0.01 | 0.913 | 0.953 |
| **honore_stat_pos** | -0.01 | 0.916 | 0.953 |
| **rate_loudness_peaks_pos** | -0.01 | 0.92 | 0.953 |
| **proportion_verb_phrase_with_subjects_neg** | -0.01 | 0.932 | 0.953 |
| **alpha_ratio_sd_neg** | -0.01 | 0.933 | 0.953 |
| **proportion_verb_phrase_with_subjects_pos** | 0.01 | 0.935 | 0.953 |
| **proportion_verb_phrase_with_objects_neg** | -0.01 | 0.948 | 0.957 |
| **pitch_mean_pos** | -0.01 | 0.951 | 0.957 |
| **local_absolute_jitter_neg** | 0.00 | 0.977 | 0.977 |

**Supp. Table S5:** Results from the moderation analysis assessing location as a moderator in the relationship between speech features and BDI-II scores. The model tested the interaction between each speech feature and location (Aachen vs. Oldenburg) using the formula: BDI ~ Speech_feature + location + speech_feature*location. The table reports the t-value and corresponding p-value for the interaction term, as well as the change in R-squared (ΔR²) between the interaction model and a model without the interaction term to estimate the effect size of the interaction.

|  | **r Oldenburg** | **r Aachen** | ***t*-value** | **ΔR²** | **adj. *p*** |
| --- | --- | --- | --- | --- | --- |
| **utterance_durations_sd_pos** | -0.20 | 0.50 | -5.04 | 0.16 | <0.001 |
| **utterance_durations_sd_neg** | -0.31 | 0.41 | -4.73 | 0.15 | 0.001 |
| **dda_shimmer_neg** | -0.43 | 0.30 | -3.73 | 0.10 | 0.01 |
| **apq11_shimmer_pos** | -0.03 | 0.33 | -3.74 | 0.09 | 0.01 |
| **local_dB_shimmer_neg** | -0.41 | 0.30 | -3.69 | 0.10 | 0.01 |
| **apq3_shimmer_neg** | -0.43 | 0.30 | -3.73 | 0.10 | 0.01 |
| **local_shimmer_neg** | -0.47 | 0.29 | -3.57 | 0.09 | <0.02 |
| **hnr_mean_neg** | 0.46 | -0.22 | 3.37 | 0.08 | <0.05 |
| **apq5_shimmer_neg** | -0.39 | 0.37 | -3.25 | 0.07 | <0.05 |
| **ddp_jitter_neg** | -0.19 | 0.37 | -3.09 | 0.07 | <0.05 |
| **utterance_durations_mean_neg** | -0.23 | 0.43 | -3.18 | 0.07 | <0.05 |
| **rap_jitter_neg** | -0.19 | 0.37 | -3.09 | 0.07 | <0.05 |
| **word_frequency_mean_neg** | 0.00 | 0.13 | -3.12 | 0.07 | <0.05 |
| **rate_loudness_peaks_pos** | 0.17 | -0.26 | 3.12 | 0.07 | <0.05 |
| **word_frequency_mean_pos** | -0.09 | -0.01 | -2.85 | 0.06 | 0.062 |
| **hnr_sd_pos** | 0.32 | -0.34 | 2.65 | 0.05 | 0.098 |
| **ppq5_jitter_neg** | -0.12 | 0.34 | -2.67 | 0.05 | 0.098 |
| **average_mfccs_1_neg** | -0.19 | 0.39 | -2.60 | 0.05 | 0.108 |
| **average_mfccs_4_pos** | -0.19 | 0.33 | -2.52 | 0.05 | 0.119 |
| **f3_frequency_mean_neg** | 0.45 | -0.01 | 2.52 | 0.05 | 0.119 |
| **hnr_sd_neg** | 0.35 | -0.25 | 2.47 | 0.05 | 0.132 |
| **apq11_shimmer_neg** | -0.31 | 0.41 | -2.44 | 0.04 | 0.134 |
| **word_frequency_sd_pos** | -0.14 | 0.05 | -2.43 | 0.04 | 0.134 |
| **local_dB_shimmer_pos** | -0.22 | 0.36 | -2.30 | 0.04 | 0.165 |
| **utterance_durations_mean_pos** | 0.00 | 0.46 | -2.30 | 0.04 | 0.165 |
| **f2_frequency_mean_neg** | 0.37 | -0.03 | 2.33 | 0.04 | 0.165 |
| **f1_relative_energy_sd_pos** | -0.43 | -0.02 | -2.26 | 0.04 | 0.172 |
| **positive_sentence_ratio_pos** | -0.18 | 0.06 | -2.23 | 0.04 | 0.179 |
| **mean_number_subordinate_clauses_pos** | -0.21 | 0.20 | -2.18 | 0.04 | 0.198 |
| **local_jitter_neg** | -0.11 | 0.33 | -2.16 | 0.04 | 0.199 |
| **determiner_rate_pos** | -0.33 | -0.08 | -2.12 | 0.03 | 0.212 |
| **f2_relative_energy_sd_pos** | 0.17 | -0.29 | 2.08 | 0.03 | 0.22 |
| **adverb_rate_pos** | -0.09 | 0.19 | -2.07 | 0.03 | 0.22 |
| **word_frequency_sd_neg** | -0.22 | 0.06 | -2.08 | 0.03 | 0.22 |
| **word_frequency_range_pos** | -0.12 | 0.18 | -2.05 | 0.03 | 0.224 |
| **speech_ratio_pos** | -0.10 | 0.31 | -2.03 | 0.03 | 0.229 |
| **rap_jitter_pos** | -0.05 | 0.42 | -2.00 | 0.03 | 0.23 |
| **ddp_jitter_pos** | -0.05 | 0.42 | -2.00 | 0.03 | 0.23 |
| **mean_sentiment_pos** | -0.19 | 0.09 | -1.95 | 0.03 | 0.25 |
| **brunets_index_neg** | 0.30 | 0.32 | -1.94 | 0.03 | 0.251 |
| **spectral_slope_500_1500_sd_neg** | -0.12 | -0.07 | -1.92 | 0.03 | 0.253 |
| **proportion_verb_phrase_with_subjects_neg** | 0.03 | 0.11 | -1.90 | 0.03 | 0.253 |
| **f1_relative_energy_sd_neg** | -0.27 | 0.00 | -1.91 | 0.03 | 0.253 |
| **adjective_rate_neg** | -0.35 | 0.10 | -1.91 | 0.03 | 0.253 |
| **spectral_slope_500_1500_mean_neg** | -0.24 | 0.20 | -1.87 | 0.03 | 0.255 |
| **f3_frequency_sd_neg** | -0.11 | 0.22 | -1.88 | 0.03 | 0.255 |
| **spectral_slope_500_1500_mean_pos** | -0.19 | 0.21 | -1.84 | 0.03 | 0.266 |
| **f3_bandwidth_sd_pos** | -0.11 | 0.03 | -1.77 | 0.02 | 0.27 |
| **word_frequency_range_neg** | 0.07 | 0.19 | -1.82 | 0.03 | 0.27 |
| **ppq5_jitter_pos** | -0.04 | 0.40 | -1.77 | 0.02 | 0.27 |
| **adverb_rate_neg** | -0.12 | 0.10 | -1.78 | 0.02 | 0.27 |
| **hnr_mean_pos** | 0.15 | -0.30 | 1.79 | 0.02 | 0.27 |
| **brunets_index_pos** | 0.18 | 0.15 | -1.80 | 0.02 | 0.27 |
| **f3_relative_energy_sd_neg** | -0.05 | 0.07 | -1.76 | 0.02 | 0.27 |
| **adposition_rate_neg** | -0.13 | 0.03 | -1.80 | 0.03 | 0.27 |
| **average_mfccs_3_pos** | 0.21 | 0.39 | -1.75 | 0.02 | 0.274 |
| **average_mfccs_1_pos** | -0.05 | 0.48 | -1.65 | 0.02 | 0.329 |
| **f3_bandwidth_mean_neg** | -0.12 | 0.14 | -1.62 | 0.02 | 0.334 |
| **pronoun_rate_pos** | -0.19 | 0.06 | -1.62 | 0.02 | 0.334 |
| **h1_h2_harmonic_difference_mean_neg** | 0.30 | 0.10 | 1.62 | 0.02 | 0.334 |
| **local_shimmer_pos** | -0.19 | 0.32 | -1.59 | 0.02 | 0.348 |
| **noun_rate_neg** | -0.25 | -0.09 | -1.56 | 0.02 | 0.363 |
| **average_mfccs_4_neg** | -0.09 | 0.37 | -1.48 | 0.02 | 0.405 |
| **negative_sentence_ratio_pos** | 0.24 | -0.12 | 1.48 | 0.02 | 0.405 |
| **f2_relative_energy_sd_neg** | -0.14 | -0.06 | -1.42 | 0.02 | 0.428 |
| **f1_bandwidth_sd_neg** | -0.05 | 0.06 | -1.38 | 0.02 | 0.428 |
| **conjunction_rate_neg** | 0.01 | 0.09 | -1.42 | 0.02 | 0.428 |
| **honore_stat_pos** | -0.12 | 0.05 | -1.40 | 0.02 | 0.428 |
| **pronoun_rate_neg** | 0.01 | 0.13 | -1.37 | 0.02 | 0.428 |
| **adjective_rate_pos** | -0.44 | -0.04 | -1.37 | 0.01 | 0.428 |
| **proportion_verb_phrase_with_objects_neg** | -0.09 | 0.10 | -1.38 | 0.02 | 0.428 |
| **inflected_verb_rate_neg** | -0.33 | 0.01 | -1.44 | 0.02 | 0.428 |
| **proportion_verb_phrase_with_subjects_pos** | 0.17 | -0.05 | -1.39 | 0.02 | 0.428 |
| **f2_bandwidth_sd_pos** | 0.18 | -0.31 | 1.37 | 0.01 | 0.428 |
| **local_absolute_jitter_neg** | -0.12 | 0.23 | -1.37 | 0.02 | 0.428 |
| **apq5_shimmer_pos** | -0.14 | 0.40 | -1.35 | 0.01 | 0.431 |
| **honore_stat_neg** | -0.16 | -0.12 | -1.31 | 0.01 | 0.456 |
| **average_mfccs_2_neg** | 0.25 | 0.13 | 1.30 | 0.01 | 0.456 |
| **f1_frequency_mean_neg** | -0.25 | -0.12 | -1.30 | 0.01 | 0.456 |
| **determiner_rate_neg** | -0.13 | -0.06 | -1.27 | 0.01 | 0.465 |
| **f1_frequency_mean_pos** | -0.19 | -0.05 | -1.26 | 0.01 | 0.465 |
| **alpha_ratio_sd_pos** | 0.28 | -0.03 | 1.28 | 0.01 | 0.465 |
| **speech_ratio_neg** | -0.06 | 0.21 | -1.27 | 0.01 | 0.465 |
| **proper_noun_rate_neg** | 0.25 | -0.08 | 1.24 | 0.01 | 0.473 |
| **f2_relative_energy_mean_neg** | -0.39 | -0.07 | -1.24 | 0.01 | 0.473 |
| **h1_h2_harmonic_difference_sd_pos** | -0.11 | -0.25 | -1.22 | 0.01 | 0.481 |
| **pause_durations_mean_neg** | 0.21 | -0.01 | -1.18 | 0.01 | 0.506 |
| **verb_phrase_with_aux_rate_pos** | -0.05 | 0.11 | -1.18 | 0.01 | 0.506 |
| **spectral_slope_500_1500_sd_pos** | -0.15 | -0.01 | -1.16 | 0.01 | 0.516 |
| **f2_frequency_sd_neg** | 0.14 | 0.07 | -1.14 | 0.01 | 0.523 |
| **f1_frequency_sd_neg** | 0.07 | 0.11 | -1.14 | 0.01 | 0.523 |
| **verb_rate_pos** | 0.04 | -0.03 | -1.13 | 0.01 | 0.525 |
| **h1_a3_harmonic_difference_sd_neg** | 0.05 | 0.01 | -1.11 | 0.01 | 0.53 |
| **h1_h2_harmonic_difference_sd_neg** | -0.02 | -0.21 | -1.11 | 0.01 | 0.53 |
| **utterance_durations_sum_neg** | 0.25 | 0.38 | 1.09 | 0.01 | 0.539 |
| **f3_bandwidth_sd_neg** | 0.04 | 0.01 | -1.07 | 0.01 | 0.541 |
| **neutral_sentence_ratio_neg** | 0.11 | 0.21 | -1.06 | 0.01 | 0.541 |
| **f1_frequency_sd_pos** | -0.10 | 0.13 | -1.06 | 0.01 | 0.541 |
| **alpha_ratio_mean_pos** | 0.25 | 0.06 | 1.05 | 0.01 | 0.541 |
| **pitch_range_neg** | -0.12 | -0.07 | -1.06 | 0.01 | 0.541 |
| **inflected_verb_rate_pos** | -0.11 | -0.06 | -1.01 | 0.01 | 0.564 |
| **f2_frequency_mean_pos** | -0.03 | 0.07 | -1.01 | 0.01 | 0.564 |
| **hammarberg_index_mean_neg** | -0.01 | 0.27 | -1.01 | 0.01 | 0.564 |
| **h1_a3_harmonic_difference_sd_pos** | 0.01 | -0.02 | -0.99 | 0.01 | 0.572 |
| **apq3_shimmer_pos** | -0.13 | 0.37 | -0.97 | 0.01 | 0.573 |
| **dda_shimmer_pos** | -0.13 | 0.37 | -0.97 | 0.01 | 0.573 |
| **duration_neg** | 0.26 | 0.33 | 0.98 | 0.01 | 0.573 |
| **spectral_slope_0_500_mean_pos** | 0.03 | -0.19 | 0.94 | 0.01 | 0.579 |
| **f3_relative_energy_mean_pos** | -0.19 | -0.20 | -0.94 | 0.01 | 0.579 |
| **pitch_std_neg** | -0.05 | -0.03 | -0.95 | 0.01 | 0.579 |
| **number_of_pauses_neg** | 0.30 | 0.29 | 0.94 | 0.01 | 0.579 |
| **pause_durations_sum_neg** | 0.29 | 0.26 | 0.93 | 0.01 | 0.58 |
| **local_absolute_jitter_pos** | -0.01 | 0.25 | -0.90 | 0.01 | 0.607 |
| **f3_frequency_sd_pos** | -0.16 | 0.25 | -0.85 | 0.01 | 0.63 |
| **number_consecutive_repetitions_neg** | 0.20 | 0.19 | -0.85 | 0.01 | 0.63 |
| **f3_frequency_mean_pos** | 0.11 | 0.01 | 0.86 | 0.01 | 0.63 |
| **f1_bandwidth_sd_pos** | -0.05 | -0.04 | -0.80 | 0.01 | 0.669 |
| **verb_phrase_with_aux_rate_neg** | -0.04 | -0.08 | -0.74 | 0.00 | 0.708 |
| **perceived_loudness_sd_pos** | 0.07 | -0.27 | 0.75 | 0.00 | 0.708 |
| **hammarberg_index_mean_pos** | 0.15 | 0.21 | 0.74 | 0.00 | 0.708 |
| **average_mfccs_3_neg** | 0.48 | 0.31 | 0.73 | 0.00 | 0.71 |
| **number_consecutive_repetitions_pos** | -0.14 | 0.08 | -0.68 | 0.00 | 0.74 |
| **f3_relative_energy_sd_pos** | 0.02 | -0.10 | 0.69 | 0.00 | 0.74 |
| **proportion_verb_phrase_with_objects_pos** | -0.08 | -0.11 | -0.66 | 0.00 | 0.742 |
| **loudness_sd_pos** | 0.00 | -0.29 | 0.66 | 0.00 | 0.742 |
| **local_jitter_pos** | 0.05 | 0.36 | -0.67 | 0.00 | 0.742 |
| **h1_a3_harmonic_difference_mean_pos** | -0.28 | -0.25 | -0.67 | 0.00 | 0.742 |
| **negative_sentence_ratio_neg** | 0.02 | -0.24 | -0.65 | 0.00 | 0.742 |
| **f2_bandwidth_sd_neg** | 0.08 | -0.29 | 0.64 | 0.00 | 0.744 |
| **f1_bandwidth_mean_neg** | -0.06 | 0.10 | -0.64 | 0.00 | 0.744 |
| **type_token_ratio_neg** | -0.19 | -0.31 | 0.63 | 0.00 | 0.747 |
| **f1_relative_energy_mean_neg** | -0.14 | -0.10 | -0.62 | 0.00 | 0.75 |
| **conjunction_rate_pos** | 0.21 | 0.07 | -0.60 | 0.00 | 0.757 |
| **word_count_neg** | 0.15 | 0.30 | 0.59 | 0.00 | 0.767 |
| **f2_bandwidth_mean_pos** | 0.18 | -0.10 | 0.57 | 0.00 | 0.777 |
| **alpha_ratio_mean_neg** | 0.27 | 0.13 | 0.56 | 0.00 | 0.783 |
| **neutral_sentence_ratio_pos** | 0.04 | 0.14 | -0.51 | 0.00 | 0.816 |
| **pitch_std_pos** | 0.04 | -0.20 | 0.50 | 0.00 | 0.816 |
| **hammarberg_index_sd_pos** | 0.14 | 0.03 | -0.51 | 0.00 | 0.816 |
| **pause_rate_pos** | 0.04 | -0.21 | 0.45 | 0.00 | 0.858 |
| **f1_relative_energy_mean_pos** | -0.04 | -0.22 | 0.43 | 0.00 | 0.871 |
| **rate_loudness_peaks_neg** | -0.10 | -0.21 | 0.39 | 0.00 | 0.882 |
| **h1_a3_harmonic_difference_mean_neg** | -0.14 | -0.29 | 0.40 | 0.00 | 0.882 |
| **positive_sentence_ratio_neg** | 0.12 | 0.23 | -0.39 | 0.00 | 0.882 |
| **utterance_durations_sum_pos** | 0.09 | 0.36 | -0.40 | 0.00 | 0.882 |
| **f3_relative_energy_mean_neg** | -0.29 | -0.15 | -0.40 | 0.00 | 0.882 |
| **adposition_rate_pos** | 0.10 | -0.03 | -0.35 | 0.00 | 0.891 |
| **noun_rate_pos** | -0.17 | -0.30 | 0.36 | 0.00 | 0.891 |
| **verb_rate_neg** | 0.11 | -0.19 | 0.36 | 0.00 | 0.891 |
| **average_mfccs_2_pos** | 0.13 | 0.18 | 0.35 | 0.00 | 0.891 |
| **pause_durations_sd_neg** | 0.25 | 0.13 | -0.34 | 0.00 | 0.893 |
| **verb_phrase_with_aux_and_vp_rate_neg** | 0.04 | 0.08 | 0.34 | 0.00 | 0.893 |
| **pitch_mean_pos** | -0.02 | -0.13 | 0.31 | 0.00 | 0.894 |
| **word_count_pos** | 0.13 | 0.15 | 0.30 | 0.00 | 0.894 |
| **spectral_slope_0_500_mean_neg** | 0.04 | -0.10 | 0.31 | 0.00 | 0.894 |
| **pitch_mean_neg** | 0.09 | -0.09 | 0.32 | 0.00 | 0.894 |
| **type_token_ratio_pos** | -0.12 | -0.16 | 0.31 | 0.00 | 0.894 |
| **f2_frequency_sd_pos** | 0.08 | 0.04 | -0.29 | 0.00 | 0.899 |
| **pitch_range_pos** | -0.09 | -0.15 | -0.27 | 0.00 | 0.91 |
| **pause_durations_sum_pos** | 0.14 | 0.12 | 0.26 | 0.00 | 0.91 |
| **pause_durations_sd_pos** | 0.20 | 0.01 | 0.26 | 0.00 | 0.91 |
| **verb_phrase_with_aux_and_vp_rate_pos** | -0.08 | -0.05 | 0.24 | 0.00 | 0.919 |
| **number_of_pauses_pos** | 0.10 | 0.23 | 0.24 | 0.00 | 0.919 |
| **loudness_sd_neg** | 0.16 | -0.12 | 0.21 | 0.00 | 0.933 |
| **spectral_slope_0_500_sd_pos** | -0.08 | -0.05 | 0.18 | 0.00 | 0.942 |
| **pause_rate_neg** | 0.14 | -0.16 | 0.19 | 0.00 | 0.942 |
| **f2_relative_energy_mean_pos** | -0.17 | -0.14 | -0.19 | 0.00 | 0.942 |
| **f1_bandwidth_mean_pos** | -0.01 | 0.14 | -0.18 | 0.00 | 0.943 |
| **mean_sentiment_neg** | 0.01 | 0.26 | 0.17 | 0.00 | 0.945 |
| **hammarberg_index_sd_neg** | 0.15 | -0.05 | -0.15 | 0.00 | 0.946 |
| **f2_bandwidth_mean_neg** | 0.18 | -0.11 | 0.15 | 0.00 | 0.946 |
| **proper_noun_rate_pos** | 0.22 | 0.03 | 0.14 | 0.00 | 0.951 |
| **pause_durations_mean_pos** | 0.13 | -0.13 | -0.13 | 0.00 | 0.951 |
| **loudness_mean_neg** | 0.12 | 0.09 | -0.10 | 0.00 | 0.962 |
| **perceived_loudness_mean_neg** | 0.10 | 0.07 | -0.10 | 0.00 | 0.962 |
| **spectral_slope_0_500_sd_neg** | -0.04 | -0.12 | -0.10 | 0.00 | 0.962 |
| **f3_bandwidth_mean_pos** | -0.10 | 0.18 | -0.09 | 0.00 | 0.962 |
| **mean_number_subordinate_clauses_neg** | -0.36 | -0.24 | -0.07 | 0.00 | 0.967 |
| **alpha_ratio_sd_neg** | 0.14 | 0.09 | -0.08 | 0.00 | 0.967 |
| **perceived_loudness_mean_pos** | 0.18 | 0.05 | 0.06 | 0.00 | 0.97 |
| **loudness_mean_pos** | 0.19 | 0.06 | 0.05 | 0.00 | 0.974 |
| **perceived_loudness_sd_neg** | 0.12 | -0.10 | 0.04 | 0.00 | 0.977 |
| **h1_h2_harmonic_difference_mean_pos** | 0.21 | 0.13 | 0.03 | 0.00 | 0.981 |
| **duration_pos** | 0.12 | 0.25 | -0.02 | 0.00 | 0.988 |

**Suppl. Table S6 -** Features selected for the best-performing models based on mutual information in the Aachen vs. Oldenburg cross-site validation.

The table contrasts the selected features for the uncorrected speech model (Column 2) and the demographically corrected model trained on residuals after regressing out the linear effects of age and education (Column 3).

| **No.** | **Features for Uncorrected Model (Aachen vs. Oldenburg)** | **Features for Demographically Corrected Model (Residuals)** |
| --- | --- | --- |
| **1** | — | adverb_rate_pos |
| **2** | apq11_shimmer_pos | — |
| **3** | — | apq3_shimmer_pos |
| **4** | apq5_shimmer_neg | — |
| **5** | average_mfccs_1_neg | — |
| **6** | average_mfccs_1_pos | average_mfccs_1_pos |
| **7** | average_mfccs_4_neg | average_mfccs_4_neg |
| **8** | average_mfccs_4_pos | — |
| **9** | — | brunets_index_neg |
| **10** | brunets_index_neg | — |
| **11** | — | dda_shimmer_pos |
| **12** | — | ddp_jitter_pos |
| **13** | duration_neg | duration_neg |
| **14** | f3_frequency_mean_neg | — |
| **15** | f3_relative_energy_sd_pos | f3_relative_energy_sd_pos |
| **16** | — | f2_bandwidth_sd_pos |
| **17** | — | f2_relative_energy_sd_neg |
| **18** | — | local_jitter_pos |
| **19** | — | mean_number_subordinate_clauses_neg |
| **20** | — | mean_tree_nodes_per_word_pos |
| **21** | — | neutral_sentence_ratio_pos |
| **22** | number_of_pauses_neg | — |
| **23** | — | number_of_pauses_pos |
| **24** | — | positive_sentence_ratio_neg |
| **25** | — | rap_jitter_pos |
| **26** | speech_ratio_pos | — |
| **27** | spectral_slope_0_500_mean_neg | — |
| **28** | type_token_ratio_pos | — |
| **29** | utterance_durations_mean_pos | — |
| **30** | utterance_durations_sd_pos | — |
| **31** | utterance_durations_sum_neg | utterance_durations_sum_neg |
| **32** | utterance_durations_sum_pos | utterance_durations_sum_pos |
| **33** | word_count_neg | — |

**Suppl. Table S7 -** Performance metrics for different classification models. BA=Balanced Accuracy; DT=Decision Trees; LM=Linear Model; RF=Random Forest; ROC-AUC=Receiver Operating Characteristic - Area under the Curve; SVM=Support Vector Machine; XT=Extra Trees.

| **Model** | **Model type (besserer Name?)** | **k features selected** | **BA** | **ROC-AUC** | **Sensitivity** | **Specificity** |
| --- | --- | --- | --- | --- | --- | --- |
| Baseline model (age, sex, years of education) | Extra Trees (XT) | 3 | 0.69 | 0.67 | 0.65 | 0.74 |
|  | Linear model (LM) | 3 | 0.45 | 0.61 | 0.75 | 0.16 |
|  | Decision Trees (DT) | 3 | 0.59 | 0.59 | 0.65 | 0.53 |
|  | Support Vector Machine (SVM) | 3 | 0.54 | 0.61 | 0.45 | 0.63 |
|  | Random Forest(RF) | 3 | 0.64 | 0.65 | 0.65 | 0.63 |
| BDI-II model | Extra Trees (XT) | 1 | 0.78 | 0.78 | 0.55 | 1.00 |
|  | Linear model (LM) | 1 | 0.78 | 0.82 | 0.55 | 1.00 |
|  | Decision Trees (DT) | 1 | 0.78 | 0.78 | 0.55 | 1.00 |
|  | Support Vector Machine (SVM) | 1 | 0.80 | 0.80 | 0.60 | 1.00 |
|  | Random Forest(RF) | 1 | 0.78 | 0.81 | 0.55 | 1.00 |
| Speech model | Extra Trees (XT) | 10 | 0.55 | 0.51 | 0.95 | 0.16 |
|  |  | 20 | 0.56 | 0.53 | 0.90 | 0.21 |
|  |  | 30 | 0.50 | 0.50 | 0.90 | 0.11 |
|  |  | 40 | 0.50 | 0.58 | 0.90 | 0.11 |
|  |  | 50 | 0.53 | 0.54 | 0.95 | 0.11 |
|  |  | 60 | 0.53 | 0.54 | 0.95 | 0.11 |
|  |  | 70 | 0.53 | 0.53 | 0.95 | 0.11 |
|  |  | 80 | 0.50 | 0.53 | 0.90 | 0.11 |
|  |  | 90 | 0.48 | 0.54 | 0.85 | 0.11 |
|  |  | 96 | 0.50 | 0.52 | 0.90 | 0.11 |
|  | Linear Model (LM) | 10 | 0.53 | 0.56 | 0.85 | 0.21 |
|  |  | 20 | 0.48 | 0.56 | 0.65 | 0.32 |
|  |  | 30 | 0.56 | 0.63 | 0.90 | 0.21 |
|  |  | 40 | 0.45 | 0.51 | 0.80 | 0.11 |
|  |  | 50 | 0.48 | 0.55 | 0.75 | 0.21 |
|  |  | 60 | 0.53 | 0.57 | 0.90 | 0.16 |
|  |  | 70 | 0.45 | 0.49 | 0.75 | 0.16 |
|  |  | 80 | 0.48 | 0.52 | 0.80 | 0.16 |
|  |  | 90 | 0.50 | 0.50 | 0.90 | 0.11 |
|  |  | 96 | 0.48 | 0.50 | 0.85 | 0.11 |
|  | Decision Tree (DT) | 10 | 0.53 | 0.53 | 0.85 | 0.21 |
|  |  | 20 | 0.56 | 0.56 | 0.85 | 0.26 |
|  |  | 30 | 0.48 | 0.48 | 0.85 | 0.11 |
|  |  | 40 | 0.59 | 0.59 | 0.70 | 0.47 |
|  |  | 50 | 0.57 | 0.57 | 0.45 | 0.68 |
|  |  | 60 | 0.51 | 0.51 | 0.65 | 0.37 |
|  |  | 70 | 0.53 | 0.53 | 0.70 | 0.37 |
|  |  | 80 | 0.64 | 0.64 | 0.55 | 0.74 |
|  |  | 90 | 0.61 | 0.61 | 0.65 | 0.58 |
|  |  | 96 | 0.64 | 0.64 | 0.55 | 0.74 |
|  | Support Vector Machine (SVM) | 10 | 0.53 | 0.55 | 0.90 | 0.16 |
|  |  | 20 | 0.56 | 0.51 | 0.85 | 0.26 |
|  |  | 30 | 0.50 | 0.58 | 0.95 | 0.05 |
|  |  | 40 | 0.53 | 0.52 | 1.00 | 0.05 |
|  |  | 50 | 0.55 | 0.56 | 1.00 | 0.11 |
|  |  | 60 | 0.55 | 0.58 | 1.00 | 0.11 |
|  |  | 70 | 0.55 | 0.54 | 1.00 | 0.11 |
|  |  | 80 | 0.55 | 0.55 | 1.00 | 0.11 |
|  |  | 90 | 0.53 | 0.50 | 0.95 | 0.11 |
|  |  | 96 | 0.50 | 0.51 | 0.90 | 0.11 |
|  | Random Forest (RF) | 10 | 0.56 | 0.54 | 0.90 | 0.21 |
|  |  | 20 | 0.53 | 0.50 | 0.85 | 0.21 |
|  |  | 30 | 0.48 | 0.54 | 0.90 | 0.05 |
|  |  | 40 | 0.50 | 0.54 | 0.90 | 0.11 |
|  |  | 50 | 0.50 | 0.58 | 0.90 | 0.11 |
|  |  | 60 | 0.53 | 0.59 | 0.95 | 0.11 |
|  |  | 70 | 0.50 | 0.61 | 0.90 | 0.11 |
|  |  | 80 | 0.50 | 0.54 | 0.90 | 0.11 |
|  |  | 90 | 0.48 | 0.57 | 0.85 | 0.11 |
|  |  | 96 | 0.53 | 0.52 | 0.90 | 0.16 |
| BDI-II & speech model | Extra Trees (XT) | 10 | 0.74 | 0.79 | 0.85 | 0.63 |
|  |  | 20 | 0.61 | 0.73 | 0.80 | 0.42 |
|  |  | 30 | 0.58 | 0.72 | 0.85 | 0.32 |
|  |  | 40 | 0.56 | 0.70 | 0.90 | 0.21 |
|  |  | 50 | 0.53 | 0.74 | 0.90 | 0.16 |
|  |  | 60 | 0.56 | 0.71 | 0.90 | 0.21 |
|  |  | 70 | 0.50 | 0.67 | 0.90 | 0.11 |
|  |  | 80 | 0.56 | 0.68 | 0.90 | 0.21 |
|  |  | 90 | 0.56 | 0.74 | 0.90 | 0.21 |
|  |  | 96 | 0.50 | 0.58 | 0.90 | 0.11 |
|  | Linear Model (LM) | 10 | 0.74 | 0.79 | 0.70 | 0.79 |
|  |  | 20 | 0.67 | 0.74 | 0.50 | 0.84 |
|  |  | 30 | 0.77 | 0.75 | 0.75 | 0.79 |
|  |  | 40 | 0.72 | 0.75 | 0.75 | 0.68 |
|  |  | 50 | 0.61 | 0.74 | 0.75 | 0.47 |
|  |  | 60 | 0.59 | 0.71 | 0.75 | 0.42 |
|  |  | 70 | 0.61 | 0.67 | 0.75 | 0.47 |
|  |  | 80 | 0.61 | 0.69 | 0.75 | 0.47 |
|  |  | 90 | 0.66 | 0.71 | 0.85 | 0.47 |
|  |  | 96 | 0.69 | 0.71 | 0.90 | 0.47 |
|  | Decision Tree (DT) | 10 | 0.80 | 0.80 | 0.65 | 0.95 |
|  |  | 20 | 0.80 | 0.80 | 0.65 | 0.95 |
|  |  | 30 | 0.80 | 0.80 | 0.65 | 0.95 |
|  |  | 40 | 0.80 | 0.80 | 0.65 | 0.95 |
|  |  | 50 | 0.80 | 0.80 | 0.65 | 0.95 |
|  |  | 60 | 0.80 | 0.80 | 0.65 | 0.95 |
|  |  | 70 | 0.80 | 0.80 | 0.65 | 0.95 |
|  |  | 80 | 0.80 | 0.80 | 0.65 | 0.95 |
|  |  | 90 | 0.80 | 0.80 | 0.65 | 0.95 |
|  |  | 96 | 0.80 | 0.80 | 0.65 | 0.95 |
|  | Support Vector Machine (SVM) | 10 | 0.58 | 0.71 | 0.90 | 0.26 |
|  |  | 20 | 0.63 | 0.70 | 0.90 | 0.37 |
|  |  | 30 | 0.58 | 0.69 | 0.90 | 0.26 |
|  |  | 40 | 0.56 | 0.66 | 0.90 | 0.21 |
|  |  | 50 | 0.63 | 0.65 | 0.95 | 0.32 |
|  |  | 60 | 0.58 | 0.64 | 0.95 | 0.21 |
|  |  | 70 | 0.53 | 0.63 | 0.95 | 0.11 |
|  |  | 80 | 0.53 | 0.58 | 0.95 | 0.11 |
|  |  | 90 | 0.53 | 0.58 | 0.90 | 0.16 |
|  |  | 96 | 0.53 | 0.58 | 0.90 | 0.16 |
|  | Random Forest (RF) | 10 | 0.77 | 0.76 | 0.60 | 0.95 |
|  |  | 20 | 0.77 | 0.83 | 0.70 | 0.84 |
|  |  | 30 | 0.64 | 0.80 | 0.85 | 0.42 |
|  |  | 40 | 0.69 | 0.82 | 0.80 | 0.58 |
|  |  | 50 | 0.69 | 0.81 | 0.80 | 0.58 |
|  |  | 60 | 0.64 | 0.81 | 0.85 | 0.42 |
|  |  | 70 | 0.61 | 0.81 | 0.90 | 0.32 |
|  |  | 80 | 0.58 | 0.77 | 0.85 | 0.32 |
|  |  | 90 | 0.61 | 0.79 | 0.85 | 0.37 |
|  |  | 96 | 0.50 | 0.72 | 0.90 | 0.11 |
